# Robustness and exploration between the interplay of the nonlinear co-dynamics HIV/AIDS and pneumonia model via fractional differential operators and a probabilistic approach

**DOI:** 10.1038/s41598-024-65329-1

**Published:** 2024-07-23

**Authors:** Saima Rashid, Sher Zaman Hamidi, Muhammad Aon Raza, Rafia Shafique, Assayel Sultan Alsubaie, Sayed K. Elagan

**Affiliations:** 1 Department of Mathematics, Government College University, Faisalabad, 38000 Pakistan; 2 Department of Computer Science and Mathematics, Lebanese American University, 11022801 Beirut, Lebanon; 3 Department of Physics, Nangarhar University, Jalalabad, Nangarhar 2601 Afghanistan; 4 Mathematics Program, Department of Science and Technology, Ranyah University College, Taif University, P.O. Box 11099, 21944 Taif, Saudi Arabia; 5 Department of Mathematics and Statistics, College of Science, Taif University, P.O. Box 11099, 21944 Taif, Saudi Arabia

**Keywords:** HIV/AIDS and pneumonia co-dynamics model, Piecewise fractional approach, Probability density functions, Qualitative analysis, Ergodic stationary distribution, Quasi-equilibrium, Biophysics, Immunology, Mathematics and computing

## Abstract

In this article, we considered a nonlinear compartmental mathematical model that assesses the effect of treatment on the dynamics of HIV/AIDS and pneumonia (H/A-P) co-infection in a human population at different infection stages. Understanding the complexities of co-dynamics is now critically necessary as a consequence. The aim of this research is to construct a co-infection model of H/A-P in the context of fractional calculus operators, white noise and probability density functions, employing a rigorous biological investigation. By exhibiting that the system possesses non-negative and bounded global outcomes, it is shown that the approach is both mathematically and biologically practicable. The required conditions are derived, guaranteeing the eradication of the infection. Furthermore, adequate prerequisites are established, and the configuration is tested for the existence of an ergodic stationary distribution. For discovering the system’s long-term behavior, a deterministic-probabilistic technique for modeling is designed and operated in MATLAB. By employing an extensive review, we hope that the previously mentioned approach improves and leads to mitigating the two diseases and their co-infections by examining a variety of behavioral trends, such as transitions to unpredictable procedures. In addition, the piecewise differential strategies are being outlined as having promising potential for scholars in a range of contexts because they empower them to include particular characteristics across multiple time frame phases. Such formulas can be strengthened via classical techniques, power law, exponential decay, generalized Mittag-Leffler kernels, probability density functions and random procedures. Furthermore, we get an accurate description of the probability density function encircling a quasi-equilibrium point if the effect of H/A-P minimizes the propagation of the co-dynamics. Consequently, scholars can obtain better outcomes when analyzing facts using random perturbations by implementing these strategies for challenging issues. Random perturbations in H/A-P co-infection are crucial in controlling the spread of an epidemic whenever the suggested circulation is steady and the amount of infection eliminated is closely correlated with the random perturbation level.

## Introduction

Human immunodeficiency virus (HIV), the causative agent of acquired immunodeficiency syndrome (AIDS), continues to be a major problem for global wellness. A majority of HIV-positive individuals (99.5%) experience the development of immunodeficiency syndrome due to HIV infection^1–3^. The infection is typically identified in intramuscular addicts, sex workers, and males who have casual encounters with men^4^. In formerly unaffected people, the illness presents a wide spectrum of pathogenic ailments and malignancies, such as a respiratory infection, Kaposi’s tumor, tuberculosis-associated pneumonia, and several other illnesses^5–8^. Inadequate lymphocyte mitotic reactions ex vivo and a decrease in T-helper lymphocyte numbers are two signs of a compromised defense against infection seen in individuals with AIDS^5–7^. HIV preferentially attacks phagocytosis and CD4$^{+}$T cells, depleting CD4$^{+}$T cells at the same time when AIDS indications actually start to appear^9,10^. Antiretroviral treatment (ART)-induced inhibition of transmission of HIV quickly boosts systemic vessel CD4$^{+}$T-cell numbers and helps the body’s defenses recover^11,12^. These findings demonstrate that the reduction of CD4$^{+}$T-cell activity and a decline in lymphocyte frequency are the primary mechanisms of insufficiency in uncontrolled patients. This reduces immune responses to microbes, leading to the emergence of abnormalities between 2 and 20 years. In terrestrial species, inoculation with transgenic simian or human antiretroviral infections replicates the impact, resulting in the prompt destruction of CD4$^{+}$T cells, AIDS, and mortality over 1–2 years^13,14^. 

Pneumonia is a respiratory-pulmonary viral illness that can be fatal, although it is curable. It is brought on by microbes, molds, or viruses. Pus builds up in air passageways known as the bronchi as a result of microbial attacks on the respiratory tract. Mucus production narrows circulation in capillary air passageways, making it harder for the afflicted individual to exhale. These infections typically reside in the noses and airways of humans and target the respiratory system when the immune system’s defenses are low. *Streptococcus pneumoniae* is a prevalent bacterial infection responsible for bronchitis by attacking the lung’s alveoli, or air passages^15^. Moisture builds up in the respiratory tract, making it more challenging for the body to acquire and transport oxygen. *Pneumocystis jirovecii* is a widespread parasite that causes bronchitis in its carriers. Pulmonary syncytial infection, or influenza A and B, is an infection responsible for bronchitis^16^. Individual concentration affects the spread of respiratory infections, which is an infection that correlates with concentration. It’s an illness that spreads via the surroundings, mostly from ingesting polluted particles. This happens when the afflicted person spits or sniffles, releasing phlegm into the atmosphere. Anyone who breathes in a contaminated environment is probably affected. Most colonization factors in cases of pneumonia infections in which individuals exhibit no indications are pneumonia viruses. Additional outbreaks and delayed interventions result from this^17^.

 If people suffering from bacteria-related pneumonia fail to receive therapy or do not react to it, the illness may be fatal. A deficiency of oxygen in the bloodstream brought on by bronchitis damages the urinary tract, heart, and nervous system, among other vital systems. Pharmaceuticals, including amoxicillin and gentamycin, that were widely used in 2012 and 2013, have the potential to alleviate sinusitis. According to findings, children in underdeveloped nations are disproportionately affected by a respiratory infection, specifically people who reside in the countryside. The probability of influenza is increased by indoor contaminants from smokers parents, dwelling in cramped quarters, and heat generated using fossil fuels (charcoal and excrement)^18^. In comparison with HIV, malnutrition, and encephalitis collectively, pneumonia is the most prevalent cause of fatalities worldwide for individuals 65 years of age and older, as well as for kids around the tender age of five. According to Gregory et al.^19^, a person with pneumonia experiences significant respiratory problems that ultimately result in mortality. Vaccination can help avoid bronchitis. Doxycycline dispersing doses are advised as a therapy in the event that the vaccination is ineffective, and the pharmaceutical industry has expanded accessibility to vaccines.

Multiple diseases occurring in the same environment are known as co-infections. Perhaps the illnesses that affect a lot of people globally are HIV/AIDS and pneumonia (H/A-P). The prevalence of concurrent infections between H/A-P and its impact on therapy results are discussed in the present study. The purpose is to investigate how medications affect patients who have both AIDS-related pneumonia and concurrent infections via HIV. Influenza can strike individuals who have depleted immunity, particularly people diagnosed with untreated HIV/AIDS. A substantial portion of deaths are caused by the concurrent infections associated with two illnesses, H/A-P as well. Pneumonia can strike those whose CD4$^{+}$T-cell count falls below 200*cells/mm*$^{3}$. Certain signs, such as a sore throat, deficits, and trouble inhaling, are noted in the co-infected individual^20^. An effective immunological mechanism enables the organism to combat respiratory infections. According to Kalipeni et al.^21^, exploitative illnesses are instances that lead to extremely significant illness and, if left untreated, mortality in HIV/AIDS survivors. HIV/AIDS compromises or entirely disables the defensive framework, making individuals more vulnerable to additional transmissible diseases. Pneumonia resulting from the fungal strain known as *Pneumocystis jirovecii* is an especially prevalent infectious condition that impacts people who possess HIV. According to the research of Polaczek et al.^20^, the association can cause a number of indications, such as a persistent cough, diminished appetite, dyspnea, trouble breathing, snoring fever, and complete muscular instability. The first phase occurs when a person has concurrent infections via H/A-P during the immediate and chronic latency phases. H/A-P is involved in the last phase of the concurrent infections of H/A-P.

However, a particularly prevalent illness that affects HIV/AIDS patients is pneumonia. If you address it, you can get well. It is the only prevalent invasive infection that contributes to the higher mortality of HIV/AIDS patients if left untreated. Due to its acute illness-causing potential, pneumonia poses a particularly substantial hazard to individuals of all ages with impaired immunological systems^22,23^. The mathematical framework of mutual infection between H/A-P is developed and analyzed in this investigation. By employing a framework of differential equations (DEs) via state variables and factors, a probabilistic framework can be implemented to provide a deeper comprehension of concurrent infection behavior, examine the closeness and consequences of the factors, estimate the behavior of the challenge over a specific time frame, and demonstrate the connections between assumptions and evaluations. 

During the years, a great deal of mathematical concepts have been developed to help us understand the world in which we live. In order to regulate presentations involving considerable obstacles, powerful artificial intelligence algorithms have been constructed, and the concept of space and time modeling has been put into practice. Some of the algorithmic techniques that are particularly commonly applied in modeling and prediction involve the idea of differentiation. DEs are scientific techniques that have been created using this concept. In the beginning endeavor, researchers suggested a number of algorithms via multifaceted associations. The variation in the compositions could include local (exchange rate, conformable derivative, and fractal derivative)^24–26^; nonlocal/singular kernel (Riemann-Liouville, Liouville-Caputo, and multiple expressions)^27^; local/non-singular kernel (Caputo-Fabrizio operators)^28^; and finally non-local/non-singular (Atangana-Baleanu-Caputo operators)^29^. For a variety of interpretations of differential derivatives or the individuals who structured the foundations, a number of academics suggest numerous novel approaches. Fractional-order (FO) calculus has a connection to realistic endeavors and finds extensive application in multiple domains such as atomic physics, optics, image encryption, nanotechnology, and infectious disease^24–26,30^.

Recently, a subfield of mathematical physics and comprehension known as fractional calculus uses FO derivatives to study how inventions and documentation operate. FO modeling, as opposed to integer-order settings, can employ reminiscence memory of the power, exponential decay, or generalized Mittag-Leffler (GML) formation kernel to capture non-local spatial-temporal interactions. The conceivable benefits of using the fractional approach by Atangana-Baleanu involve all non-localities that are inherent within the explanation, just like in all previous variations. However, the most significant characteristic is that it has a nonsingular and non-local kernel, represented by the GML functionality, which, from an empirical viewpoint, includes the clarification and advancement of competencies delineated by a series of privileges. Kumar et al.^31^ contemplated a new investigation on fractional HBV models through Caputo and Atangana-Baleanu-Caputo derivatives. Mekkaoui et al.^32^ presented the predictor-corrector for non-linear DEs and integral equations with fractional operators. Atangana and Araz^33^ described a successive midpoint method for nonlinear DEs with integer and Caputo-Fabrizio derivatives. On the other hand, it has been shown that the previously mentioned approach precisely conveys the complex compositions of many practical representations^29,33^. The piecewise derivative, which has recently gained prominence^34^, was presented by Atangana and Araz^35^ and distinguishes from every derivative by the fact that it may reprise the interconnected paths that comprise these fractional algorithms in a differentiation technique. Every aspect that happens demonstrates that while the prevalence of the co-dynamics of H/A-P is probabilistic instead of deterministic in nature, knowledgeable research is based on an empirical methodology. A number of academics investigated the real-world growth of viruses and bacteria using the fundamental concept of probabilistic modeling, as reported in^36,37^.

 Certain probabilistic HIV/AIDS individuals via pneumonia concurrent infection outbreak frameworks using the death and replenishment of viremia and target cells have been successfully defined to examine the influence of probabilistic white noise and offer several efficient initiatives for governing infection interactions. These frameworks are founded on the randomly generated linear disruptive methodology, which assumes the biological nature of ambient white noise correlates to the dimension of every compartment. Moreover, it has been demonstrated experimentally that a probabilistic H/A-P system that includes owest-viremia patients and virus controllers can prevent an epidemic of co-infection. Motivated by these findings^38,39^, we also presume that the random perturbation is closely connected to specific populations of the evolution of H/A-P diagnostics. In order to illustrate the significant influence of a probabilistic framework condition mentioned in^40^, we performed this work to create this paper. Additionally, we create a probabilistic mathematical structure utilizing piecewise fractional derivative expressions to analyze the co-infection process, incorporating the average initial CD4$^{+}$T cell level in a chronic HIV infection following the acute phase of infection within predetermined time intervals $[0,\intercal ]$. In order to achieve this, we separate the population into two groups: the incidence and occurrence of exacerbated immune dysregulation and decreased lymphocyte function, along with erroneous variations. The probability that the most recent H/A-P will be engaged is represented by the proportion $\psi \in (0,1)$, whereas the unexplained component $1-\psi$ will not be implicated. In addition, we established the global positive solutions of the co-infection model with a unique ergodic and stationary distribution (ESD) technique to illustrate the biological properties and statistical viability of this structure. We also provide the precise definition of the probabilistic density function (P.D.F) at a quasi-equilibrium point that represents the probabilistic pneumonia approach, which reflects significant spontaneous features in probabilistic relevance. The ESD and P.D.F surrounding the quasi-equilibrium point of the randomized multidimensional co-dynamics framework will be better understood as a result of this investigation. The intention of the investigation is to acquire an improved comprehension of how the infection persists over time in the probabilistic co-dynamics system. In general, fractional operators examine simulations conducted numerically of the proposed system that include crossover structures and white noise.

## Mathematical formulation of H/A-P model and preliminaries

Inspired by the numerical simulation studies conducted in actual scenarios by different researchers^38–40^, we suggested an integer-order co-infection framework for the evolution of H/A-P transmission. The entire individual group taken into consideration in the present investigation at time $\textbf{t}$, denoted by $\mathbb {N}(\textbf{t})$, is divided into nine independently different classes outlined below in order to characterize and create the suggested pairing structure: the variety of persons indicated by $\textbf{S}(\textbf{t})$ who are at risk of contracting pneumonia or HIV/AIDS, the quantity of individuals shielded from contracting pneumonia as indicated by ${\textbf{P}}_{\textbf{m}_{1}}(\textbf{t})$, the figure ${\textbf{H}}_{\textbf{m}_{1}}(\textbf{t})$ represents the number of people who use condoms to prevent HIV infection, the sole variable that indicates the overall number of pneumonia cases is ${\textbf{P}}(\textbf{t})$, the sole indication of the quantity of HIV-positive individuals is ${\textbf{H}}(\textbf{t})$, the total population with AIDS, as shown by ${\textbf{H}}_{\textbf{X}}(\textbf{t})$, ${\textbf{Y}}({\textbf{t}})$ represents the entire population of individuals who have both pneumonia and HIV/AIDS, the percentage of patients receiving treatment for HIV/AIDS is shown by ${\textbf{T}}_{\textbf{H}\textbf{X}}(\textbf{t})$, ${\textbf{P}}_{\textbf{T}}(\textbf{t})$ represents the whole amount of individuals having pneumonia and the overall count of people taken into account for this research is indicated by

1 $$\begin{aligned} \mathbb {N}(\textbf{t})={\textbf{S}}(\textbf{t})+{\textbf{P}}_{\textbf{m}_{1}}(\textbf{t})+{\textbf{H}}_{\textbf{m}_{1}}(\textbf{t})+{\textbf{H}}(\textbf{t})+{\textbf{H}}_{\textbf{X}}(\textbf{t})+{\textbf{P}}(\textbf{t})+{\textbf{Y}}(\textbf{t})+{\textbf{P}}_{\textbf{T}}(\textbf{t})+{\textbf{T}}_{\textbf{H}\textbf{X}}(\textbf{t}). \end{aligned}$$

HIV/AIDS is described as a destructive agent epidemic that infects sensitive individuals:

2 $$\begin{aligned} \chi _{\textbf{H}}(\textbf{t})=\frac{\delta _{1}}{\mathbb {N}}\big ({\textbf{H}}(\textbf{t})+\tilde{\omega _{1}}{\textbf{H}}_{\textbf{X}}(\textbf{t})+{\Im _{2}}{\textbf{Y}}(\textbf{t})\big ), \end{aligned}$$

where $\delta _{1}$ is the degree in which HIV/AIDS propagates and $\tilde{\omega _{1}}\in [1,+\infty )$ and ${\Im _{2}}\in [1,+\infty )$ are the mutation factors that boost a human being’s infectiousness. 

When vulnerable individuals get pneumonia, the level of illness is determined by

3 $$\begin{aligned} \chi _{\textbf{q}}(\textbf{t})=\frac{\delta _{2}}{\mathbb {N}}\big ({\textbf{P}}(\textbf{t})+\omega {\textbf{Y}}(\textbf{t})\big ), \end{aligned}$$

where $\delta _{2}$ represents the level when pneumonia progresses and $\omega \in [1,\infty )$ is the adjustment factor that promotes transmission.

Assumptions for developing the suggested pairing framework for HIV/AIDS and pneumonia are as follows: a certain number of the overall registered individuals $\Lambda$ who are joining the pneumonia-safety cohort ${\textbf{P}}_{\textbf{m}_{1}}(\textbf{t})$ are represented by $\textbf{m}_{1},~\textbf{m}_{2}$ and $1-\textbf{m}_{1}-\textbf{m}_{2},$ respectively, to the vulnerable category ${\textbf{S}}(\textbf{t})$ and the HIV-protected group ${\textbf{H}}_{\textbf{m}_{1}}(\textbf{t}),$ in that order; the entire population is heterogeneous and not pervasive. HIV cannot spread vertically or from person to person, while there is no contemporaneous dual-infection spread of HIV with pneumonia. Pneumonia recovery following therapy is also not everlasting.

**Figure 1:**
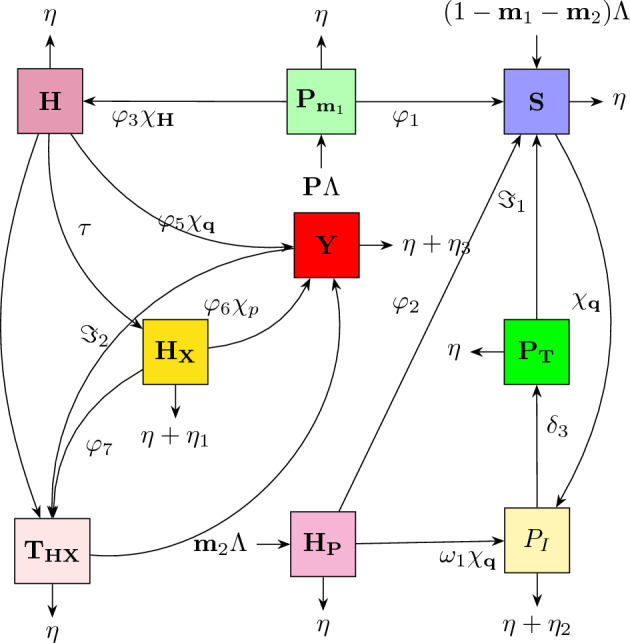
Flow diagram for depicting the co-dynamics process of H/A-P model (4).

 Figure 1 depicts the process diagram for the complexities of HIV/AIDS and pneumonia combination propagation, which depends on Table 1 as well as the framework specifications and hypotheses mentioned earlier.

According to Fig. 1, the framework of nonlinear DEs for the pairing system is derived in the following manner:

4 $$\begin{aligned} {\left\{ \begin{array}{ll} \dot{\textbf{S}}(\textbf{t})=(1-\textbf{m}_{1}-\textbf{m}_{2})\Lambda +\varphi _{1}{\textbf{P}}_{\textbf{m}_{1}}+\varphi _{2}{\textbf{H}}_{\textbf{m}_{1}}+{\Im _{1}}{\textbf{P}}_{\textbf{T}}-(\chi _{\textbf{H}}+\chi _{\textbf{q}}+\eta ){\textbf{S}},\\ \dot{{\textbf{P}}_{\textbf{m}_{1}}}(\textbf{t})=\textbf{m}_{1}\Lambda -(\varphi _{3}\chi _{\textbf{H}}+\varphi _{1}+\eta ){\textbf{P}}_{\textbf{m}_{1}},\\ \dot{{\textbf{H}}_{\textbf{m}_{1}}}(\textbf{t})=\textbf{m}_{2}\Lambda -(\varphi _{2}+\eta +\omega _{1}\chi _{\textbf{q}}){\textbf{H}}_{\textbf{m}_{1}},\\ \dot{{\textbf{H}}_{1}}(\textbf{t})=\chi _{\textbf{H}}{\textbf{S}}+\varphi _{3}\chi _{\textbf{H}}{\textbf{P}}_{\textbf{m}_{1}}-(\eta +\tau +\varphi _{4}+\varphi _{5}\chi _{\textbf{q}}){\textbf{H}},\\ \dot{{\textbf{H}}_{\textbf{X}}}(\textbf{t})=\tau {\textbf{H}}-(\eta +\eta _{1}+\varphi _{7}+\varphi _{6}\chi _{\textbf{q}}){\textbf{H}}_{\textbf{X}},~~~~~~~~0\le \textbf{t}\le \intercal _{1},\\ \dot{\textbf{P}}(\textbf{t})=\chi _{\textbf{q}}{\textbf{S}}+\omega _{1}\chi _{\textbf{q}}{\textbf{H}}_{\textbf{m}_{1}}-(\eta +\eta _{2}+\delta _{3}+\zeta \chi _{\textbf{H}}){\textbf{P}},\\ \dot{{\textbf{Y}}}(\textbf{t})=\zeta \chi _{\textbf{H}}{\textbf{P}}+\varphi _{5}\chi _{\textbf{q}}{\textbf{H}}+\varphi _{6}\chi _{\textbf{q}}{\textbf{H}}_{\textbf{X}}+\sigma \chi _{\textbf{q}}{\textbf{T}}_{\textbf{H}\textbf{X}}-(\eta +\eta _{3}+{\Im _{2}}){\textbf{Y}},\\ \dot{{\textbf{P}}_{\textbf{T}}}=\delta _{3}{\textbf{P}}-(\eta +{\Im _{1}}){\textbf{P}}_{\textbf{T}},\\ \dot{{\textbf{T}}_{\textbf{H}\textbf{X}}}(\textbf{t})=\varphi _{4}\textbf{H}+\varphi _{7}{\textbf{H}}_{\textbf{X}}+{\Im _{2}}{\textbf{Y}}-(\eta +\omega _{1}\chi _{\textbf{q}}){\textbf{T}}_{\textbf{H}\textbf{X}}, \end{array}\right. } \end{aligned}$$

containing positive initial conditions (ICs) ${\textbf{S}}(0)\ge 0,~{\textbf{P}}_{\textbf{m}_{1}}(0)\ge 0,~{\textbf{H}}_{\textbf{m}_{1}}(0)\ge 0,~{\textbf{H}}_{1}(0)\ge 0,~{\textbf{H}}_{\textbf{X}}(0)\ge 0,~{\textbf{P}}(0)\ge 0,~{\textbf{Y}}(0)\ge 0,~~{\textbf{P}}_{\textbf{T}}(0)\ge 0,~{\textbf{T}}_{\textbf{H}\textbf{X}}(0)\ge 0.$

**Table 1:** Components of the pairing approach.

Symbols	Description
${\textbf{S}}$	Predisposed persons
${\textbf{P}}_{\textbf{m}_{1}}$	Individuals that are immune to pneumonia
${\textbf{H}}_{\textbf{m}_{1}}$	Individuals that are immune to HIV/AIDS
${\textbf{H}}$	HIV-positive people
${\textbf{H}}_{\textbf{X}}$	AIDS afflicted
${\textbf{P}}$	Individuals suffering from pneumonia
${\textbf{Y}}$	Individuals who also have pneumonia and HIV/AIDS
${\textbf{P}}_{\textbf{T}}$	Individuals receiving treatment for pneumonia
${\textbf{T}}_{\textbf{H}\textbf{X}}$	Those receiving treatment for HIV/AIDS
$\eta$	Rate for spontaneous death
$\Lambda$	Recruiting rate of individuals
$\varphi _{1}$	Failure incidence from pneumonia prevention
$\varphi _{2}$	HIV prevention reduction percentage
${\Im _{2}}$	Intervention percentage for individuals contaminated with HIV/AIDS and pneumonia
$\varphi _{5},\varphi _{6}$	Adjustment factors
$\zeta$	Adjustment factors
$\eta _{1}$	Mortality toll from AIDS
$\eta _{2}$	Mortality toll from pneumonia
$\eta _{3}$	Mortality rate from co-dynamics
$\delta _{3}$	Intervention percentage for pneumonia
$\varphi _{4}$	Prevalence of HIV-related therapy
$\varphi _{7}$	Therapy frequency for AIDS patients
$\delta _{1}$	Rate prevalence of HIV/AIDS
$\delta _{2}$	Rate prevalence of pneumonia
$\textbf{m}_{1}$	Percentage of the recruiting trend
$\textbf{m}_{2}$	Percentage of the recruiting trend
${\Im _{1}}$	Rate of susceptibility response to pneumonia
$\omega$	Adjustment factors

To help readers that are acquainted with fractional calculus, we provide the related summary herein (see^27–29^).

$$\begin{aligned} \,_{0}^{c}\textbf{D}_{\textbf{t}}^{\beta } \mathcal {G}(\textbf{t})=\frac{1}{\Gamma (1-\beta )}\int \limits _{0}^{\textbf{t}}\mathcal {G}^{\prime }(\textbf{q})(\textbf{t}-\textbf{q})^{\beta }d\textbf{q},~~\beta \in (0,1]. \end{aligned}$$

The index kernel is involved in the Caputo fractional derivative (CFD). Whenever experimenting with a particular integral transform, such as the Laplace transform^41,42^, the CFD accommodates regular ICs.

$$\begin{aligned} \,_{0}^{CF}\textbf{D}_{\textbf{t}}^{\beta } \mathcal {G}(\textbf{t})=\frac{\bar{\mathcal {M}}(\beta )}{1-\beta }\int \limits _{0}^{\textbf{t}}\mathcal {G}^{\prime }(\textbf{q})\exp \bigg [-\frac{\beta }{1-\beta }(\textbf{t}-\textbf{q})\bigg ]d\textbf{q},~~\beta \in (0,1], \end{aligned}$$

where $\bar{\mathcal {M}}(\beta )$ indicates the normalization function $\bar{\mathcal {M}}(0)=\bar{\mathcal {M}}(1)=1.$

The non-singular kernel of the Caputo-Fabrizio fractional derivative (CFFD) operator has drawn the attention of numerous researchers. Furthermore, representing an assortment of prevalent issues that obey the exponential decay memory is best suited to utilize the CFFD operator^43^. With the passage of time, developing a mathematical model using the CFFD became a remarkable field of research. In recent times, several mathematicians have been busy with the development and simulation of CFFD DEs^44^. 

The ABC fractional derivative operator is described as follows:

$$\begin{aligned} \,_{0}^{ABC}\textbf{D}_{\textbf{t}}^{\beta } \mathcal {G}(\textbf{t})=\frac{ABC(\beta )}{1-\beta }\int \limits _{0}^{\textbf{t}}\mathcal {G}^{\prime }(\textbf{q})E_{\beta }\bigg [-\frac{\beta }{1-\beta }(\textbf{t}-\textbf{q})^{\beta }\bigg ]d\textbf{q},~~\beta \in (0,1], \end{aligned}$$

where $ABC(\beta )=1-\beta +\frac{\beta }{\Gamma (\beta )}$ represents the normalization function. 

The memory utilized in Atangana-Baleanu-Caputo fractional derivative (ABCFD) can be found intuitively within the index-law analogous for an extended period as well as exponential decay in a number of scientific concerns^45,46^. The broad scope of the connection and the non-power-law nature of the underlying tendency are the driving forces behind the selection of this version. The impact of the kernel, considered crucial in the dynamic Baggs–Freedman framework, was fully produced by the GML function^47^. 

To far better perceive the propagation of H/A-P, we indicate a dynamic mechanism (4) that includes the co-infection within the context of CFD, CFFD and ABCFD, respectively. This is because FO algorithms possess inherited properties that characterize the local/non-local and singular/non-singular dynamics of natural phenomena, presented as follows:

5 $$\begin{aligned}{} & {} {\left\{ \begin{array}{ll} \,^{c}\textbf{D}_{\textbf{t}}^{\beta }{\textbf{S}}=(1-\textbf{m}_{1}-\textbf{m}_{2})\Lambda +\varphi _{1}{\textbf{P}}_{\textbf{m}_{1}}+\varphi _{2}{\textbf{H}}_{\textbf{m}_{1}}+{\Im _{1}}{\textbf{P}}_{\textbf{T}}-(\chi _{\textbf{H}}+\chi _{\textbf{q}}+\eta ){\textbf{S}},\\ \,^{c}\textbf{D}_{\textbf{t}}^{\beta }{{\textbf{P}}_{\textbf{m}_{1}}}=\textbf{m}_{1}\Lambda -(\varphi _{3}\chi _{\textbf{H}}+\varphi _{1}+\eta ){\textbf{P}}_{\textbf{m}_{1}},\\ \,^{c}\textbf{D}_{\textbf{t}}^{\beta }{{\textbf{H}}_{\textbf{m}_{1}}}=\textbf{m}_{2}\Lambda -(\varphi _{2}+\eta +\omega _{1}\chi _{\textbf{q}}){\textbf{H}}_{\textbf{m}_{1}},\\ \,^{c}\textbf{D}_{\textbf{t}}^{\beta }{{\textbf{H}}_{1}}=\chi _{\textbf{H}}{\textbf{S}}+\varphi _{3}\chi _{\textbf{H}}{\textbf{P}}_{\textbf{m}_{1}}-(\eta +\tau +\varphi _{4}+\varphi _{5}\chi _{\textbf{q}}){\textbf{H}},\\ \,^{c}\textbf{D}_{\textbf{t}}^{\beta }{{\textbf{H}}_{\textbf{X}}}=\tau {\textbf{H}}-(\eta +\eta _{1}+\varphi _{7}+\varphi _{6}\chi _{\textbf{q}}){\textbf{H}}_{\textbf{X}},~~~~~~~~~~~~~~~~\intercal _{1}\le \textbf{t}\le \intercal _{2},\\ \,^{c}\textbf{D}_{\textbf{t}}^{\beta }{\textbf{P}}=\chi _{\textbf{q}}{\textbf{S}}+\omega _{1}\chi _{\textbf{q}}{\textbf{H}}_{\textbf{m}_{1}}-(\eta +\eta _{2}+\delta _{3}+\zeta \chi _{\textbf{H}}){\textbf{P}},\\ \,^{c}\textbf{D}_{\textbf{t}}^{\beta }{{\textbf{Y}}}=\zeta \chi _{\textbf{H}}{\textbf{P}}+\varphi _{5}\chi _{\textbf{q}}{\textbf{H}}+\varphi _{6}\chi _{\textbf{q}}{\textbf{H}}_{\textbf{X}}+\sigma \chi _{\textbf{q}}{\textbf{T}}_{\textbf{H}\textbf{X}}-(\eta +\eta _{3}+{\Im _{2}}){\textbf{Y}},\\ \,^{c}\textbf{D}_{\textbf{t}}^{\beta }{{\textbf{P}}_{\textbf{T}}}=\delta _{3}{\textbf{P}}-(\eta +{\Im _{1}}){\textbf{P}}_{\textbf{T}},\\ \,^{c}\textbf{D}_{\textbf{t}}^{\beta }{{\textbf{T}}_{\textbf{H}\textbf{X}}}=\varphi _{4}\textbf{H}+\varphi _{7}{\textbf{H}}_{\textbf{X}}+{\Im _{2}}{\textbf{Y}}-(\eta +\omega _{1}\chi _{\textbf{q}}){\textbf{T}}_{\textbf{H}\textbf{X}}, \end{array}\right. } \end{aligned}$$

6 $$\begin{aligned}{} & {} {\left\{ \begin{array}{ll} \,^{CF}\textbf{D}_{\textbf{t}}^{\beta }{\textbf{S}}=(1-\textbf{m}_{1}-\textbf{m}_{2})\Lambda +\varphi _{1}{\textbf{P}}_{\textbf{m}_{1}}+\varphi _{2}{\textbf{H}}_{\textbf{m}_{1}}+{\Im _{1}}{\textbf{P}}_{\textbf{T}}-(\chi _{\textbf{H}}+\chi _{\textbf{q}}+\eta ){\textbf{S}},\\ \,^{CF}\textbf{D}_{\textbf{t}}^{\beta }{{\textbf{P}}_{\textbf{m}_{1}}}=\textbf{m}_{1}\Lambda -(\varphi _{3}\chi _{\textbf{H}}+\varphi _{1}+\eta ){\textbf{P}}_{\textbf{m}_{1}},\\ \,^{CF}\textbf{D}_{\textbf{t}}^{\beta }{{\textbf{H}}_{\textbf{m}_{1}}}=\textbf{m}_{2}\Lambda -(\varphi _{2}+\eta +\omega _{1}\chi _{\textbf{q}}){\textbf{H}}_{\textbf{m}_{1}},\\ \,^{CF}\textbf{D}_{\textbf{t}}^{\beta }{{\textbf{H}}_{1}}=\chi _{\textbf{H}}{\textbf{S}}+\varphi _{3}\chi _{\textbf{H}}{\textbf{P}}_{\textbf{m}_{1}}-(\eta +\tau +\varphi _{4}+\varphi _{5}\chi _{\textbf{q}}){\textbf{H}},\\ \,^{CF}\textbf{D}_{\textbf{t}}^{\beta }{{\textbf{H}}_{\textbf{X}}}=\tau {\textbf{H}}-(\eta +\eta _{1}+\varphi _{7}+\varphi _{6}\chi _{\textbf{q}}){\textbf{H}}_{\textbf{X}},~~~~~~~~~~~~~~~~\intercal _{1}\le \textbf{t}\le \intercal _{2},\\ \,^{CF}\textbf{D}_{\textbf{t}}^{\beta }{\textbf{P}}=\chi _{\textbf{q}}{\textbf{S}}+\omega _{1}\chi _{\textbf{q}}{\textbf{H}}_{\textbf{m}_{1}}-(\eta +\eta _{2}+\delta _{3}+\zeta \chi _{\textbf{H}}){\textbf{P}},\\ \,^{CF}\textbf{D}_{\textbf{t}}^{\beta }{{\textbf{Y}}}=\zeta \chi _{\textbf{H}}{\textbf{P}}+\varphi _{5}\chi _{\textbf{q}}{\textbf{H}}+\varphi _{6}\chi _{\textbf{q}}{\textbf{H}}_{\textbf{X}}+\sigma \chi _{\textbf{q}}{\textbf{T}}_{\textbf{H}\textbf{X}}-(\eta +\eta _{3}+{\Im _{2}}){\textbf{Y}},\\ \,^{CF}\textbf{D}_{\textbf{t}}^{\beta }{{\textbf{P}}_{\textbf{T}}}=\delta _{3}{\textbf{P}}-(\eta +{\Im _{1}}){\textbf{P}}_{\textbf{T}},\\ \,^{CF}\textbf{D}_{\textbf{t}}^{\beta }{{\textbf{T}}_{\textbf{H}\textbf{X}}}=\varphi _{4}\textbf{H}+\varphi _{7}{\textbf{H}}_{\textbf{X}}+{\Im _{2}}{\textbf{Y}}-(\eta +\omega _{1}\chi _{\textbf{q}}){\textbf{T}}_{\textbf{H}\textbf{X}}, \end{array}\right. } \end{aligned}$$

7 $$\begin{aligned}{} & {} {\left\{ \begin{array}{ll} \,^{ABC}\textbf{D}_{\textbf{t}}^{\beta }{\textbf{S}}=(1-\textbf{m}_{1}-\textbf{m}_{2})\Lambda +\varphi _{1}{\textbf{P}}_{\textbf{m}_{1}}+\varphi _{2}{\textbf{H}}_{\textbf{m}_{1}}+{\Im _{1}}{\textbf{P}}_{\textbf{T}}-(\chi _{\textbf{H}}+\chi _{\textbf{q}}+\eta ){\textbf{S}},\\ \,^{ABC}\textbf{D}_{\textbf{t}}^{\beta }{{\textbf{P}}_{\textbf{m}_{1}}}=\textbf{m}_{1}\Lambda -(\varphi _{3}\chi _{\textbf{H}}+\varphi _{1}+\eta ){\textbf{P}}_{\textbf{m}_{1}},\\ \,^{ABC}\textbf{D}_{\textbf{t}}^{\beta }{{\textbf{H}}_{\textbf{m}_{1}}}=\textbf{m}_{2}\Lambda -(\varphi _{2}+\eta +\omega _{1}\chi _{\textbf{q}}){\textbf{H}}_{\textbf{m}_{1}},\\ \,^{ABC}\textbf{D}_{\textbf{t}}^{\beta }{{\textbf{H}}_{1}}=\chi _{\textbf{H}}{\textbf{S}}+\varphi _{3}\chi _{\textbf{H}}{\textbf{P}}_{\textbf{m}_{1}}-(\eta +\tau +\varphi _{4}+\varphi _{5}\chi _{\textbf{q}}){\textbf{H}},\\ \,^{ABC}\textbf{D}_{\textbf{t}}^{\beta }{{\textbf{H}}_{\textbf{X}}}=\tau {\textbf{H}}-(\eta +\eta _{1}+\varphi _{7}+\varphi _{6}\chi _{\textbf{q}}){\textbf{H}}_{\textbf{X}},~~~~~~~~~~~~~~~~\intercal _{1}\le \textbf{t}\le \intercal _{2},\\ \,^{ABC}\textbf{D}_{\textbf{t}}^{\beta }{\textbf{P}}=\chi _{\textbf{q}}{\textbf{S}}+\omega _{1}\chi _{\textbf{q}}{\textbf{H}}_{\textbf{m}_{1}}-(\eta +\eta _{2}+\delta _{3}+\zeta \chi _{\textbf{H}}){\textbf{P}},\\ \,^{ABC}\textbf{D}_{\textbf{t}}^{\beta }{{\textbf{Y}}}=\zeta \chi _{\textbf{H}}{\textbf{P}}+\varphi _{5}\chi _{\textbf{q}}{\textbf{H}}+\varphi _{6}\chi _{\textbf{q}}{\textbf{H}}_{\textbf{X}}+\sigma \chi _{\textbf{q}}{\textbf{T}}_{\textbf{H}\textbf{X}}-(\eta +\eta _{3}+{\Im _{2}}){\textbf{Y}},\\ \,^{ABC}\textbf{D}_{\textbf{t}}^{\beta }{{\textbf{P}}_{\textbf{T}}}=\delta _{3}{\textbf{P}}-(\eta +{\Im _{1}}){\textbf{P}}_{\textbf{T}},\\ \,^{ABC}\textbf{D}_{\textbf{t}}^{\beta }{{\textbf{T}}_{\textbf{H}\textbf{X}}}=\varphi _{4}\textbf{H}+\varphi _{7}{\textbf{H}}_{\textbf{X}}+{\Im _{2}}{\textbf{Y}}-(\eta +\omega _{1}\chi _{\textbf{q}}){\textbf{T}}_{\textbf{H}\textbf{X}}. \end{array}\right. } \end{aligned}$$

The arrangement of this article is as follows: In “Mathematical formulation of H/A-P model and preliminaries”, explanations for fractional calculus, along with several key notions and model (4) details, are provided. Moreover, a detailed analysis of the FO co-infection system’s (5) equilibrium stability is presented in In “Mathematical formulation of H/A-P model and preliminaries”. In “Stochastic configuration of co-dynamics of H/A-P model”, a probabilistic form of the H/A-P models’ (16) co-dynamics is proposed and a detailed description of the unique global positive solution for each positive initial requirement is presented. The dynamical characteristics of the mechanism’s appropriate conditions for the presence of the distinctive stationary distribution are provided. The P.D.F enclosing a quasi-stable equilibrium of the probabilistic pneumonia framework is presented in “Analysis of pneumonia”. Numerous numerical simulations in view of piecewise fractional derivative operators are presented in “Numerical procedure for H/A-P model using random perturbations” to validate the diagnostic findings we obtained in “Stochastic configuration of co-dynamics of H/A-P model” and in “Analysis of pneumonia”. In conclusion, we conceal our findings to conclude this study.

### Positivity and boundedness

Since we interact with living communities, each approach ought to be constructive and centred on a workable area. We utilized the subsequent hypothesis that guarantees these.

#### Theorem 2.1

*Assume that the set* $\tilde{\Xi }:=\Big ({\textbf{S}},{\textbf{P}}_{\textbf{m}_{1}},{\textbf{H}}_{\textbf{m}_{1}},{\textbf{H}},{\textbf{H}}_{\textbf{X}},{\textbf{P}},{\textbf{Y}},{\textbf{P}}_{\textbf{T}},{\textbf{T}}_{\textbf{H}\textbf{X}}\Big )$ *is a positive invariant set for the suggested FO model* (5).

#### Proof

In order to demonstrate whether the solution to a set of equations (5) is positive, then (5) yields

8 $$\begin{aligned} {\left\{ \begin{array}{ll} \,^{c}\textbf{D}_{\textbf{t}}^{\beta }{\textbf{S}}\big \vert _{\textbf{S}=0}=(1-\textbf{m}_{1}-\textbf{m}_{2})\Lambda \ge 0,\\ \,^{c}\textbf{D}_{\textbf{t}}^{\beta }{{\textbf{P}}_{\textbf{m}_{1}}}\big \vert _{{\textbf{P}}_{\textbf{m}_{1}}=0}=\textbf{m}_{1}\Lambda \ge 0,\\ \,^{c}\textbf{D}_{\textbf{t}}^{\beta }{{\textbf{H}}_{\textbf{m}_{1}}}\big \vert _{{\textbf{H}}_{\textbf{m}_{1}}=0}=\textbf{m}_{2}\Lambda \ge 0,\\ \,^{c}\textbf{D}_{\textbf{t}}^{\beta }{{\textbf{H}}_{1}}\big \vert _{{\textbf{H}}=0}=\chi _{\textbf{H}}{\textbf{S}}+\varphi _{3}\chi _{\textbf{H}}{\textbf{P}}_{\textbf{m}_{1}}\ge 0,\\ \,^{c}\textbf{D}_{\textbf{t}}^{\beta }{{\textbf{H}}_{\textbf{X}}}\big \vert _{{\textbf{H}}_{\textbf{X}}=0}=\tau {\textbf{H}}\ge 0,\\ \,^{c}\textbf{D}_{\textbf{t}}^{\beta }{\textbf{P}}\big \vert _{{\textbf{P}}=0}=\chi _{\textbf{q}}{\textbf{S}}+\omega _{1}\chi _{\textbf{q}}{\textbf{H}}_{\textbf{m}_{1}}\ge 0,\\ \,^{c}\textbf{D}_{\textbf{t}}^{\beta }{{\textbf{Y}}}\big \vert _{{\textbf{Y}}=0}=\zeta \chi _{\textbf{H}}{\textbf{P}}+\varphi _{5}\chi _{\textbf{q}}{\textbf{H}}+\varphi _{6}\chi _{\textbf{q}}{\textbf{H}}_{\textbf{X}}\ge 0,\\ \,^{c}\textbf{D}_{\textbf{t}}^{\beta }{{\textbf{P}}_{\textbf{T}}}\big \vert _{{\textbf{P}}_{\textbf{T}}=0}=\delta _{3}{\textbf{P}}\ge 0,\\ \,^{c}\textbf{D}_{\textbf{t}}^{\beta }{{\textbf{T}}_{\textbf{H}\textbf{X}}}\big \vert _{{\textbf{T}}_{\textbf{H}\textbf{X}}=0}=\varphi _{4}\textbf{H}+\varphi _{7}{\textbf{H}}_{\textbf{X}}+{\Im _{2}}{\textbf{Y}}\ge 0, \end{array}\right. } \end{aligned}$$

Therefore, the outcomes related to the FO model (5) are positive. Finally, the variation in the entire community is described by

$$\begin{aligned} \,_{0}^{c}\textbf{D}_{\textbf{t}}^{\beta }\tilde{\Xi }{} & {} \le \Lambda -\eta \mathbb {N}-\big (\eta _{1}{\textbf{H}}_{\textbf{X}}+\eta _{2}{\textbf{P}}+\eta _{3}{\textbf{Y}}\big )\nonumber \\ {}{} & {} \Lambda -\eta \mathbb {N}. \end{aligned}$$

Addressing the variant previously mentioned, we get

$$\begin{aligned} \tilde{\Xi }(\tau )\le \big (\tilde{\Xi }(0)-\frac{\Lambda }{\eta }\big )E_{\beta }(-\eta {\textbf{t}}^{\beta })+\frac{\Lambda }{\eta }. \end{aligned}$$

Consequently, we derive the GML function’s asymptotic operation^48^ as

$$\begin{aligned} \tilde{\Xi }(\textbf{t})\le \frac{\Lambda }{\eta }. \end{aligned}$$

Adopting the same procedure for other systems of equations in the model (5), which indicates that the closed set $\tilde{\Xi }$ is a positive invariant domain for the FO system (5). $\square$

- Assuming that every requirement is non-negative throughout time $\textbf{t}$, we exhibit that the outcomes remain non-negative and bounded in the proposed region, $\Pi$. We’ll look at the co-infection model (5) $\tilde{\Xi }:=\Big ({\textbf{S}},{\textbf{P}}_{\textbf{m}_{1}},{\textbf{H}}_{\textbf{m}_{1}},{\textbf{H}},{\textbf{H}}_{\textbf{X}},{\textbf{P}},{\textbf{Y}},{\textbf{P}}_{\textbf{T}},{\textbf{T}}_{\textbf{H}\textbf{X}}\Big )$ spreads in the domain, which is described as $\Pi :=\Big \{\tilde{\Xi }\in \Re _{+}^{9}:0\le \textbf{N}\le \frac{\Lambda }{\eta }\Big \}.$
- According to the afflicted categories in co-infection model (5), disease-free equilibrium (DFE) and endemic equilibrium (EE) are the biologically significant steady states of FO model (5). We establish the fractional derivative to get the immune-to-infection steady state as $\,_{0}^{c}\textbf{D}_{\textbf{t}}^{\beta }{\textbf{S}},~\,_{0}^{c}\textbf{D}_{\textbf{t}}^{\beta }{{\textbf{P}}}_{\textbf{m}_{1}},~\,_{0}^{c}\textbf{D}_{\textbf{t}}^{\beta }{{\textbf{H}}}_{\textbf{m}_{1}},\,_{0}^{c}\textbf{D}_{\textbf{t}}^{\beta }{\textbf{H}},~\,_{0}^{c}\textbf{D}_{\textbf{t}}^{\beta }{{\textbf{H}}_{\textbf{X}}}, \,_{0}^{c}\textbf{D}_{\textbf{t}}^{\beta }{{\textbf{P}}},~\,_{0}^{c}\textbf{D}_{\textbf{t}}^{\beta }{{\textbf{Y}}},~\,_{0}^{c}\textbf{D}_{\textbf{t}}^{\beta }{{\textbf{P}}_{\textbf{T}}},~\,_{0}^{c}\textbf{D}_{\textbf{t}}^{\beta }{{\textbf{T}}_{\textbf{H}\textbf{X}}}$ to zero of the FO model (5) have no infection, and get
  $$\begin{aligned} \mathcal {E}_{\textbf{H}\textbf{P}}^{0}{} & {} =\Big (\textbf{S}^{0},{{\textbf{P}}}_{\textbf{m}_{1}}^{0},{{\textbf{H}}}_{\textbf{m}_{1}}^{0},{\textbf{H}}^{0},{{\textbf{H}}_{\textbf{X}}}^{0},{{\textbf{P}}}^{0},{{\textbf{Y}}}^{0},{{\textbf{P}}_{\textbf{T}}}^{0},{{\textbf{T}}_{\textbf{H}\textbf{X}}}^{0}\Big )\nonumber \\ {}{} & {} =\Big (\frac{(1-\textbf{m}_{1}-\textbf{m}_{2})\Lambda (\eta +\varphi _{1})(\eta +\varphi _{2})+\varphi _{1}\textbf{m}_{1}\Lambda +\varphi _{2}\textbf{m}_{2}\Lambda (\eta +\varphi _{1})}{\eta (\eta +\varphi _{1})(\eta +\varphi _{2})},\frac{\varphi _{1}\Lambda }{\eta +\varphi _{1}},\frac{\textbf{m}_{2}\Lambda }{\eta +\varphi _{2}},0,0,0,0,0,0\Big ) \end{aligned}$$
- The dominating eigenvalue of the matrix $\textbf{F}\textbf{G}^{-1}$ correlates with the basic reproductive quantity $\mathbb {R}_{\textbf{H}\textbf{P}}^{0}$ of structure (5), in accordance with the next generation matrix approach^49^. Thus, we find
  $$\begin{aligned} \mathcal {F}\mathcal {V}^{-1}= \begin{pmatrix} \frac{\delta _{1}(\tilde{\omega _{1}}+\eta (1-\textbf{m}_{2}))}{(\eta +\varphi _{2})(\eta +\varphi _{4}+\tau )}+\frac{\delta _{1}\eta \tilde{\omega _{1}}(1-\textbf{m}_{2})+\delta _{1}\varphi _{2}\tilde{\omega _{1}}}{(\eta +\varphi _{2})(\eta +\eta _{1}+\varphi _{7})}&{}0&{}0&{}0&{}0&{}0\\ 0&{}\frac{\delta _{2}(\varphi _{1}+\eta (1-\textbf{m}_{1}))}{(\eta +\varphi _{1})(\eta +\eta _{2}+\delta _{3})}&{}0&{}0&{}0\\ 0&{}0&{}0&{}0&{}0\\ 0&{}0&{}0&{}0&{}0\\ 0&{}0&{}0&{}0&{}0\\ 0&{}0&{}0&{}0&{}0 \end{pmatrix}. \end{aligned}$$
  The next generation matrix $\mathcal {F}\mathcal {V}^{-1}$ is determined by the dominating eigenvalue in intensity of the pairing model (4) fundamental reproductive quantity, which is provided by
  9 $$\begin{aligned} \mathbb {R}_{\textbf{H}\textbf{P}}^{0}{} & {} =\max \big \{\mathbb {R}_{\textbf{H}}^{0},\mathbb {R}_{\textbf{P}}^{0}\big \}\nonumber \\ {}{} & {} =\max \bigg \{ \frac{\delta _{1}(\tilde{\omega _{1}}+\eta (1-\textbf{m}_{2}))}{(\eta +\varphi _{2})(\eta +\varphi _{4}+\tau )}+\frac{\delta _{1}\eta \tilde{\omega _{1}}(1-\textbf{m}_{2})+\delta _{1}\varphi _{2}\tilde{\omega _{1}}}{(\eta +\varphi _{2})(\eta +\eta _{1}+\varphi _{7})},\frac{\delta _{2}(\varphi _{1}+\eta (1-\textbf{m}_{1}))}{(\eta +\varphi _{1})(\eta +\eta _{2}+\delta _{3})}\bigg \}, \end{aligned}$$
  where $\mathbb {R}_{\textbf{P}}^{0}$ stands for pneumonia simply, $\mathbb {R}_{\textbf{H}}^{0}$ for HIV/AIDS-only, and $\mathbb {R}_{\textbf{H}\textbf{P}}^{0}$ for pairing fundamental reproducing numbers, respectively.
  Here, the anticipated amount of additional transmissible illnesses generated by a single contaminated person throughout the whole duration of transmission in the entirety of the vulnerable populace, as well as the DFEP provided by, are the fundamental reproduction numbers of H/A-P, in the same manner as the unattached diseases $\mathcal {E}_{\textbf{H}\textbf{P}}^{0}$ is locally asymptotically stable iff $\mathbb {R}_{\textbf{H}\textbf{P}}^{0}<1$ and unstable if $\mathbb {R}_{\textbf{H}\textbf{P}}^{0}>1.$
- By assigning the values for each DE to zero, we are able to estimate the endemic equilibrium points (EEPs) of the complete pairing system (5), and the conclusion that follows is provided by
  10 $$\begin{aligned}{} & {} {\textbf{S}}^{*}=\frac{(1-\textbf{m}_{1}-\textbf{m}_{2})\Lambda +\varphi _{1}{\textbf{P}}_{\textbf{m}_{1}}^{*}+\varphi _{2}{\textbf{H}}_{\textbf{m}_{1}}^{*}+{\Im _{1}}{\textbf{P}}_{\textbf{T}}^{*}}{\chi _{\textbf{H}}^{*}+\chi _{\textbf{q}}^{*}+\eta },~~~{\textbf{P}}_{\textbf{m}_{1}}^{*}=\frac{\textbf{m}_{1}\Lambda }{(\varphi _{3}\chi _{\textbf{H}}^{*}+\varphi _{1}+\eta )},~~~{\textbf{H}}_{\textbf{m}_{1}}^{*}=\frac{\textbf{m}_{2}\Lambda }{(\varphi _{3}\varphi _{2}+\eta +\omega _{1}\chi _{\textbf{q}}^{*})},\nonumber \\ {}{} & {} {\textbf{H}}^{*}=\frac{\chi _{\textbf{H}}^{*}{\textbf{S}}^{*}+\varphi _{3}\chi _{\textbf{H}}^{*}{\textbf{P}}_{\textbf{m}_{1}}^{*}}{(\eta +\varphi _{4}+\tau +\varphi _{5}\chi _{\textbf{q}}^{*})},~~~~{\textbf{H}}_{\textbf{X}}^{*}=\frac{\tau {\textbf{H}}^{*}}{(\eta +\eta _{1}+\varphi _{7}+\varphi _{6}\chi _{\textbf{q}}^{*})},~~~~{\textbf{P}}^{*}=\frac{\chi _{\textbf{q}}^{*}{\textbf{S}}^{*}+\varphi _{3}\chi _{\textbf{q}}^{*}{\textbf{H}}_{\textbf{m}_{1}}^{*}}{(\delta _{3}+\eta +\eta _{2}+\zeta \chi _{\textbf{H}}^{*})},\nonumber \\ {}{} & {} {\textbf{Y}}^{*}=\frac{\zeta \chi _{\textbf{H}}^{*}{\textbf{P}}^{*}+\varphi _{5}\chi _{\textbf{q}}^{*}{\textbf{H}}^{*}+\varphi _{6}\chi _{\textbf{q}}^{*}{\textbf{H}}_{\textbf{X}}^{*}+\sigma \chi _{\textbf{q}}^{*}{\textbf{T}}_{\textbf{H}\textbf{X}}^{*}}{(\eta +\eta _{3}+{\Im _{2}})},~~~~{\textbf{P}}_{\textbf{T}}^{*}=\frac{\delta _{3}{\textbf{P}}^{*}}{(\eta +{\Im _{1}})},~~~~{\textbf{T}}_{\textbf{H}\textbf{X}}^{*}=\frac{\varphi _{4}{\textbf{H}}^{*}+\varphi _{7}{\textbf{H}}_{\textbf{X}}^{*}+{\Im _{2}}{\textbf{Y}}^{*}}{(\eta +\sigma \chi _{\textbf{q}}^{*})}. \end{aligned}$$
  Because of the excessive nonlinearity of the co-infection system (5) that we suggested, it is challenging to compute the EEPS explicitly in regard to the demonstrated system factors mathematically. Nevertheless, in accordance with the results of previous investigations of the H/A-P variants, the EEPs indicated by $\mathcal {E}_{\textbf{H}\textbf{P}}^{*}=\big ({\textbf{S}}^{*},{\textbf{P}}_{\textbf{m}_{1}}^{*},{\textbf{H}}_{\textbf{m}_{1}}^{*},{\textbf{H}}^{*},{\textbf{H}}_{\textbf{X}}^{*},{\textbf{P}}^{*},{\textbf{Y}}^{*},{\textbf{P}}_{\textbf{T}}^{*},{\textbf{T}}_{\textbf{H}\textbf{X}}^{*}\big )$ have developed every $\mathbb {R}_{\textbf{H}}^{0}>1$ and $\mathbb {R}_{\textbf{P}}^{0}>1$, i.e., $\mathbb {R}_{\textbf{H}\textbf{P}}^{0}>1$. In the quantitative modeling section, the stability is displayed.
-  Assume that $\mathbb {N}=\varkappa _{1}+\varkappa _{2}+\varkappa _{3}+\varkappa _{4}+\varkappa _{5}+\varkappa _{6}+\varkappa _{7}+\varkappa _{8}+\varkappa _{9}$ gives the entire populace of humans while $\textbf{S}=\varkappa _{1},~{\textbf{P}}_{\textbf{m}_{1}}=\varkappa _{2},~{\textbf{H}}_{\textbf{m}_{1}}=\varkappa _{3},~{\textbf{H}}=\varkappa _{4},~{\textbf{H}}_{\textbf{X}}=\varkappa _{5},~{\textbf{P}}=\varkappa _{6},~{\textbf{Y}}=\varkappa _{7},~{\textbf{P}}_{\textbf{T}}=\varkappa _{8}$ and ${\textbf{T}}_{\textbf{H}\textbf{X}}=\varkappa _{9}.$ This means that the nonlinear systems (5) will be expressed as $\dot{\textbf{Z}}(\textbf{t})=\mathcal {H}(\textbf{Z})$ having $\mathcal {H}=(\textbf{f}_{1},\textbf{f}_{2},\textbf{f}_{3},\textbf{f}_{4},\textbf{f}_{5},\textbf{f}_{6},\textbf{f}_{7},\textbf{f}_{8},\textbf{f}_{9})^{\textbf{T}}$ whilst the vector description $\textbf{Z}=(\varkappa _{1},\varkappa _{2},\varkappa _{3},\varkappa _{4},\varkappa _{5},\varkappa _{6},\varkappa _{7},\varkappa _{8},\varkappa _{9})^{\textbf{T}}$ and
  11 $$\begin{aligned} {\left\{ \begin{array}{ll} \textbf{f}_{1}=\dot{\varkappa _{1}}(\textbf{t})=(1-\textbf{m}_{1}-\textbf{m}_{2})\Lambda +\varphi _{1}\varkappa _{2}+\varphi _{2}\varkappa _{3}+{\Im _{1}} \varkappa _{8}-(\chi _{\textbf{H}}+\chi _{\textbf{q}}+\eta ){\varkappa _{1}},\\ \textbf{f}_{2}=\dot{\varkappa _{2}}(\textbf{t})=\textbf{m}_{1}\Lambda -(\varphi _{3}\chi _{\textbf{H}}+\varphi _{1}+\eta )\varkappa _{2},\\ \textbf{f}_{3}=\dot{\varkappa _{3}}(\textbf{t})=\textbf{m}_{2}\Lambda -(\varphi _{2}+\eta +\omega _{1}\chi _{\textbf{q}})\varkappa _{3},\\ \textbf{f}_{4}= \dot{\varkappa _{4}}(\textbf{t})=\chi _{\textbf{H}}{\varkappa _{1}}+\varphi _{3}\chi _{\textbf{H}}\varkappa _{2}-(\eta +\tau +\varphi _{4}+\varphi _{5}\chi _{\textbf{q}})\varkappa _{4},\\ \textbf{f}_{5}= \dot{\varkappa _{5}}(\textbf{t})=\tau {\varkappa _{4}}-(\eta +\eta _{1}+\varphi _{7}+\varphi _{6}\chi _{\textbf{q}})\varkappa _{5},\\ \textbf{f}_{6}= \dot{\varkappa _{6}}(\textbf{t})=\chi _{\textbf{q}}{\varkappa _{1}}+\omega _{1}\chi _{\textbf{q}}\varkappa _{3}-(\eta +\eta _{2}+\delta _{3}+\zeta \chi _{\textbf{H}}){\varkappa _{6}},\\ \textbf{f}_{7}= \dot{{\varkappa _{7}}}(\textbf{t})=\zeta \chi _{\textbf{H}}{\varkappa _{6}}+\varphi _{5}\chi _{\textbf{q}}{\varkappa _{4}}+\varphi _{6}\chi _{\textbf{q}}\varkappa _{5}+\sigma \chi _{\textbf{q}}\varkappa _{9}-(\eta +\eta _{3}+{\Im _{2}})\varkappa _{7},\\ \textbf{f}_{8}= \dot{\varkappa _{8}}=\delta _{3}{\varkappa _{6}}-(\eta +{\Im _{1}})\varkappa _{8},\\ \textbf{f}_{9}= \dot{\varkappa _{9}}(\textbf{t})=\varphi _{4}\varkappa _{4}+\varphi _{7}\varkappa _{5}+{\Im _{2}}{\varkappa _{7}}-(\eta +\omega _{1}\chi _{\textbf{q}})\varkappa _{9}, \end{array}\right. } \end{aligned}$$
  where $$\chi _{\textbf{H}}=\frac{\delta _{1}}{\mathbb {N}}\big (\varkappa _{1}+\tilde{\omega _{1}}\varkappa _{5}+{\Im _{2}} \varkappa _{7}\big )$$ and $$\chi _{\textbf{q}}=\frac{\delta _{2}}{\mathbb {N}}(\varkappa _{6}+\omega \varkappa _{7}),~\omega ,\tilde{\omega _{1}}\in [1,\infty ).$$

Next, the newly created nonlinear technique’s Jacobian matrix, denoted by $\mathbb {J}_{\mathcal {\textbf{H}\textbf{P}}}^{0}$ was identified, as provided in (11) at $\mathcal {E}_{{\textbf{H}\textbf{P}}}^{0}$ expressed as

$$\begin{aligned} \mathbb {J}_{\mathcal {E}_{\textbf{H}\textbf{P}}}^{0}=\begin{pmatrix} -\eta &{}\varphi _{1}&{}\varphi _{2}&{}\mathcal {E}_{1}&{}\mathcal {E}_{2}&{}\mathcal {E}_{3}&{}\mathcal {E}_{4}&{}{\Im _{1}}&{}0\\ 0&{}-(\eta +\varphi _{1})&{}0&{}\mathcal {E}_{5}&{}\mathcal {E}_{6}&{}0&{}\mathcal {E}_{7}&{}0&{}0\\ 0&{}0&{}-(\eta +\varphi _{2})&{}0&{}0&{}\mathcal {E}_{8}&{}\mathcal {E}_{9}&{}0&{}0\\ 0&{}0&{}0&{}\mathcal {E}_{10}&{}\mathcal {E}_{11}&{}0&{}\mathcal {E}_{12}&{}0&{}0\\ 0&{}0&{}0&{}\tau &{}-(\eta +\eta _{1}+\varphi _{7})&{}0&{}0&{}0&{}0\\ 0&{}0&{}0&{}0&{}0&{}\mathcal {E}_{13}&{}\mathcal {E}_{14}&{}0&{}0\\ 0&{}0&{}0&{}0&{}0&{}0&{}-(\eta +\eta _{1}+{\Im _{2}})&{}0&{}0\\ 0&{}0&{}0&{}0&{}0&{}\delta _{3}&{}0&{}-(\eta +{\Im _{1}})&{}0\\ 0&{}0&{}0&{}\varphi _{4}&{}\varphi _{7}&{}0&{}{\Im _{2}}&{}0&{}-\eta \end{pmatrix}, \end{aligned}$$

where $$\mathcal {E}_{1}=-\delta _{1}\varkappa _{1}^{0}/\mathbb {N}^{0},\, \mathcal {E}_{2}=-\delta _{1}\beta \varkappa _{1}^{0}/\mathbb {N}^{0},\, \mathcal {E}_{3}=-\delta _{2}\varkappa _{1}^{0}/\mathbb {N},\, \mathcal {E}_{4}=-\delta _{1}{\Im _{2}} \varkappa _{1}^{0}/\mathbb {N}^{0}-\delta _{2}\omega \varkappa _{1}^{0}/\mathbb {N}^{0},\, \mathcal {E}_{5}=-\delta _{1}\varkappa _{2}^{0}/\mathbb {N}^{0},\, \, \mathcal {E}_{6}=\delta _{1}\varphi _{3}\tilde{\omega _{1}}\varkappa _{2}^{0}/\mathbb {N}^{0},\, \mathcal {E}_{7}=-\delta _{1}\sigma _{1}\varkappa _{2}^{0}/\mathbb {N}^{0},\mathcal {E}_{8}=-\delta _{2}\varkappa _{3}^{0}/\mathbb {N}^{0},\, \mathcal {E}_{9}=-\delta _{2}\omega \varkappa _{3}^{0}/\mathbb {N}^{0},\, \mathcal {E}_{10}=\delta _{1}\varkappa _{1}^{0}/\mathbb {N}^{0}+\delta _{1}\varphi _{3} \varkappa _{2}^{0}/\mathbb {N}-(\eta +\tau +\varphi _{4}),\, \mathcal {E}_{11}=\delta _{1}\tilde{\omega _{1}}\varkappa _{1}^{0}/\mathbb {N}^{0}+\delta _{1}\varphi _{3} \varkappa _{2}^{0}/\mathbb {N}^{0},\, \mathcal {E}_{12}=\delta _{1}{\Im _{2}} \varkappa _{1}^{0}/\mathbb {N}^{0}+\delta _{1}\varphi _{3}{\Im _{2}} \varkappa _{2}^{0}/\mathbb {N}^{0},\, \mathcal {E}_{13}=\delta _{2}\varkappa _{1}^{0}/\mathbb {N}^{0}+\delta _{2}\varkappa _{3}^{0}/\mathbb {N}^{0}-(\delta _{3}+\eta +\eta _{2}),\, \mathcal {E}_{14}=\delta _{2}\omega \varkappa _{1}^{0}/\mathbb {N}^{0}+\delta _{2}\omega \varkappa _{3}^{0}/\mathbb {N}^{0}.$$

Without loss of the generality, let’s suppose that $\mathbb {R}_{\textbf{H}}^{0}<\mathbb {R}_{\textbf{P}}^{0}$ and that $\mathbb {R}_{\textbf{H}}^{0}=1$, or $\mathbb {R}_{\textbf{P}}^{0}=1$. Furthermore, suppose that $\delta _{2}=\beta ^{*}$ is a bifurcation factor. After utilizing $\mathbb {R}_{\textbf{P}}^{0}=1$ to solve for $\delta _{2}$, we obtained $\beta ^{*}=\delta _{2}=(\eta +\eta _{2}+\delta _{3})(\eta +\varphi _{1})/\eta (1-\textbf{m}_{1})+\varphi _{1}.$

Eventually, we evaluate the eigenvalues of the Jacobian matrix $\mathbb {J}(\mathcal {E}_{\textbf{H}\textbf{P}}^{0})$ at $\mathcal {E}_{\textbf{H}\textbf{P}}^{0},$ for $\delta _{2}=\beta ^{*}$, thus we find the eigenvalues provided by

12 $$\begin{aligned}{} & {} \varpi _{1}=-\eta<0,~~\varpi _{2}=-(\varphi _{1}+\eta )<0,~~~~\varpi _{3}=-(\eta +\varphi _{2})<0,~~~\varpi _{4}=(\eta +\tau +\varphi _{4})\big (\mathbb {R}_{\textbf{H}}^{0}-1\big )<0,~~~~\varpi _{5}=0,\nonumber \\ {}{} & {} \varpi _{6}=(\delta _{3}+\eta +\eta _{2})\big (\mathbb {R}_{\textbf{P}}^{0}-1\big )<0,~~~\varpi _{7}=-(\eta +\sigma +{\Im _{2}})<0,~~~\varpi _{8}=-(\eta +{\Im _{1}})<0,~~\varpi _{9}=-\eta <0. \end{aligned}$$

Based on the calculations, we found that if $\mathbb {R}_{\textbf{H}\textbf{P}}<1$, then all of the eigenvalues are negative. We demonstrate that the nonlinear structure (5) experiences the phenomenon of forward bifurcation at $\mathbb {R}_{\textbf{P}}^{0}=1,$ by applying the center manifold hypothesis proposed in^50^. The Jacobian $\mathbb {J}_{\beta ^{*}}$ eigenvectors for the situation $\mathbb {R}_{\textbf{P}}^{0}=1$ may be found with the following eigenvectors at $\delta _{2}=\beta ^{*}$: these correlate to the zero eigenvalue, which is provided by $\bar{y}=(p_{1},p_{2},p_{3},p_{4},p_{5},p_{6},p_{7},p_{8},p_{9})^{\textbf{T}}.$ Therefore, we have

13 $$\begin{aligned}{} & {} p_{1}=\frac{\varphi _{2}(\eta +{\Im _{1}})p_{5}\mathcal {E}_{7}+(\eta +\varphi _{2})(\eta +{\Im _{1}})p_{5}\mathcal {E}_{2}+{\Im _{1}}\delta _{3} p_{5}(\eta +\varphi _{2})}{\eta (\varphi _{2}+\eta )(\eta +{\Im _{1}})},~~~p_{2}=0,~~~p_{3}=\mathcal {E}_{7}p_{5}/(\eta +\varphi _{2}),\nonumber \\ {}{} & {} p_{4}=0,~~p_{5}>0~~,p_{6}=0,~~~p_{7}=\kappa p_{5}/\eta +{\Im _{1}},~~~p_{8}=0,~~p_{9}=0. \end{aligned}$$

The leftmost eigenvectors that correlate to the zero eigenvalue at $\delta _{2}=\beta ^{*}$ and which maintain $\bar{y}\bar{z}=1$ are as follows: $\bar{z}=(g_{1},g_{2},g_{3},g_{4},g_{5},g_{6},g_{7},g_{8},g_{9})$ and $g_{1}=g_{2}=g_{3}=g_{4}=g_{5}=g_{6}=g_{7}=g_{8}=g_{9}$ and $g_{5}=g_{6}>0.$

We calculated and simplified a number of times before determining the bifurcation parameters, denoted by $\bar{a}$ and $\bar{b}$, as

14 $$\begin{aligned} \bar{a}{} & {} =2g_{5}p_{1}p_{5}\frac{\partial ^{2}\textbf{f}_{5}(0,0)}{\partial \varkappa _{1}\partial \varkappa _{5}}+2g_{5}p_{3}p_{5}\frac{\partial ^{2}\textbf{f}_{5}(0,0)}{\partial \varkappa _{2}\partial \varkappa _{5}}=2\delta _{2}^{*}g_{5}p_{5}[p_{1}+p_{3}]\nonumber \\ {}{} & {} =2\delta _{2}^{*}g_{5}p_{5}^{2}\Big \{\frac{-\varphi _{2}\delta _{2}\varkappa _{3}^{0}(\eta +{\Im _{1}})-(\eta +\varphi _{2})(\eta +{\Im _{1}})\delta _{2}\varkappa _{1}^{0}-(\eta +\varphi _{2}){\Im _{1}}\delta _{3}-\eta (\eta +{\Im _{1}})\delta _{2}\varkappa _{3}^{0}}{\eta (\eta +\varphi _{2})(\eta +{\Im _{1}})}\Big \}. \end{aligned}$$

Finally, we have

15 $$\begin{aligned} \bar{a}{} & {} =-2\delta _{2}^{*}g_{5}p_{5}^{2}\Big \{\frac{\varphi _{2}\delta _{2}\varkappa _{3}^{0}(\eta +{\Im _{1}})+(\eta +\varphi _{2})(\eta +{\Im _{1}})\delta _{2}\varkappa _{1}^{0}+(\eta +\varphi _{2}){\Im _{1}}\delta _{3}-\eta (\eta +{\Im _{1}})\delta _{2}\varkappa _{3}^{0}}{\eta (\eta +\varphi _{2})(\eta +{\Im _{1}})}\Big \}, \nonumber \\ \bar{b}{} & {} =g_{5}p_{5}\frac{\partial ^{2}\textbf{f}_{5}(0,0)}{\partial \varkappa _{5}\partial \delta _{2}}=g_{5}p_{5}(\varkappa _{3}^{0}+\varkappa _{1}^{0})>0. \end{aligned}$$

Thus, the H/A-P pairing nonlinear structure (5) does not display the phenomenon of backward bifurcation where $\mathbb {R}_{\textbf{H}\textbf{P}}^{0}=\mathbb {R}_{\textbf{P}}^{0}=1$, according to the specifications given in^50^. Therefore, in the zone where $\mathbb {R}_{\textbf{H}\textbf{P}}^{0}<1$, there exists just a pairing model DFEP and no detected EEP.

## Stochastic configuration of co-dynamics of H/A-P model

We examine how random interference affects the distinctiveness and presence of a stable dispersion, as well as the gradual disappearance of diseases, in the system (4). The formula that follows is a representation of the stochastic adaptation relating to the model (4) is

16 $$\begin{aligned} {\left\{ \begin{array}{ll} d{\textbf{S}}=\big [(1-\textbf{m}_{1}-\textbf{m}_{2})\Lambda +\varphi _{1}{\textbf{P}}_{\textbf{m}_{1}}+\varphi _{2}{\textbf{H}}_{\textbf{m}_{1}}+{\Im _{1}}{\textbf{P}}_{\textbf{T}}-(\chi _{\textbf{H}}+\chi _{\textbf{q}}+\eta ){\textbf{S}}\big ]d\textbf{t}+\wp _{1}{\textbf{S}}d\mathbb {W}_{1}(\textbf{t}),\\ d{{\textbf{P}}_{\textbf{m}_{1}}}=\big [\textbf{m}_{1}\Lambda -(\varphi _{3}\chi _{\textbf{H}}+\varphi _{1}+\eta ){\textbf{P}}_{\textbf{m}_{1}}\big ]d\textbf{t}+\wp _{2}{{\textbf{P}}_{\textbf{m}_{1}}}d\mathbb {W}_{2}(\textbf{t}),\\ d{{\textbf{H}}_{\textbf{m}_{1}}}=\big [\textbf{m}_{2}\Lambda -(\varphi _{2}+\eta +\omega _{1}\chi _{\textbf{q}}){\textbf{H}}_{\textbf{m}_{1}}\big ]d\textbf{t}+\wp _{3}{{\textbf{H}}_{\textbf{m}_{1}}}d\mathbb {W}_{3}(\textbf{t}),\\ d{{\textbf{H}}_{1}}=\big [\chi _{\textbf{H}}{\textbf{S}}+\varphi _{3}\chi _{\textbf{H}}{\textbf{P}}_{\textbf{m}_{1}}-(\eta +\tau +\varphi _{4}+\varphi _{5}\chi _{\textbf{q}}){\textbf{H}}\big ]d\textbf{t}+\wp _{4}{{\textbf{H}}_{1}}d\mathbb {W}_{4}(\textbf{t}),\\ d{{\textbf{H}}_{\textbf{X}}}=\big [\tau {\textbf{H}}-(\eta +\eta _{1}+\varphi _{7}+\varphi _{6}\chi _{\textbf{q}}){\textbf{H}}_{\textbf{X}}\big ]d\textbf{t}+\wp _{5}{{\textbf{H}}_{\textbf{X}}}d\mathbb {W}_{5}(\textbf{t}),~~~~~~~~~~~~~~~~\intercal _{2}\le \textbf{t}\le \intercal ,\\ d{\textbf{P}}=\big [\chi _{\textbf{q}}{\textbf{S}}+\omega _{1}\chi _{\textbf{q}}{\textbf{H}}_{\textbf{m}_{1}}-(\eta +\eta _{2}+\delta _{3}+\zeta \chi _{\textbf{H}}){\textbf{P}}\big ]d\textbf{t}+\wp _{6}{{\textbf{P}}_{1}}d\mathbb {W}_{6}(\textbf{t}),\\ d{{\textbf{Y}}}=\big [\zeta \chi _{\textbf{H}}{\textbf{P}}+\varphi _{5}\chi _{\textbf{q}}{\textbf{H}}+\varphi _{6}\chi _{\textbf{q}}{\textbf{H}}_{\textbf{X}}+\sigma \chi _{\textbf{q}}{\textbf{T}}_{\textbf{H}\textbf{X}}-(\eta +\eta _{3}+{\Im _{2}}){\textbf{Y}}\big ]d\textbf{t}+\wp _{7}{{\textbf{Y}}}d\mathbb {W}_{7}(\textbf{t}),\\ d{{\textbf{P}}_{\textbf{T}}}=\big [\delta _{3}{\textbf{P}}-(\eta +{\Im _{1}}){\textbf{P}}_{\textbf{T}}\big ]d\textbf{t}+\wp _{8}{{\textbf{P}}_{\textbf{T}}}d\mathbb {W}_{8}(\textbf{t}),\\ d{{\textbf{T}}_{\textbf{H}\textbf{X}}}=\big [\varphi _{4}\textbf{H}+\varphi _{7}{\textbf{H}}_{\textbf{X}}+{\Im _{2}}{\textbf{Y}}-(\eta +\omega _{1}\chi _{\textbf{q}}){\textbf{T}}_{\textbf{H}\textbf{X}}\big ]d\textbf{t}+\wp _{9}{{\textbf{P}}_{\textbf{m}_{1}}}d\mathbb {W}_{9}(\textbf{t}), \end{array}\right. } \end{aligned}$$

in which $\wp _{\jmath }$ indicate the variability in noise and ${\mathbb {W}_{\jmath }}(\textbf{t}),~~(\jmath =1,...,9)$ are conventional one-dimensional autonomous Brownian movements. The additional parameters have the same relevance as they do in system (4).

 In the sequel, let $(\tilde{\Upsilon },\mathfrak {F},\{\mathfrak {F}_{\textbf{t}}\}_{\textbf{t}\ge 0},\mathbb {P})$ be a complete probability space and its filtration $\{\mathfrak {F}_{\textbf{t}}\}_{\textbf{t}\ge 0}$ needs to fulfill the standard requirements (that is., it must be right continuous and comprise all $\mathbb {P}$-null sets), whilst ${\mathbb {W}}_{\jmath }(\textbf{t}),~(\jmath =1,...,9)$ are stated on the complete probability space. In addition, take $\mathbb {R}_{+}=\big \{\Upsilon \ge 0\big \},~\mathbb {R}_{+}^{9}=\big \{\Upsilon =(\Upsilon _{1},...,\Upsilon _{9})\in \mathbb {R}^{9}:\Upsilon _{\jmath }>0,~\jmath =1,...,9\big \}.$ For any matrix $\mathbb {M},$ its transpose is indicated by $\mathbb {M}^{\bar{T}}.$

Utilizing $\Upsilon (\textbf{t})=\big ({\textbf{S}}(\textbf{t}),{\textbf{P}}_{\textbf{m}_{1}}(\textbf{t}),{\textbf{H}}_{\textbf{m}_{1}}(\textbf{t}),{\textbf{H}}_{1}(\textbf{t}),{\textbf{H}}_{\textbf{X}}(\textbf{t}),{\textbf{P}}(\textbf{t}),{\textbf{Y}}(\textbf{t}),{\textbf{P}}_{\textbf{T}}(\textbf{t}),{\textbf{T}}_{\textbf{H}\textbf{X}}(\textbf{t})\big )^{\bar{T}}$ as the solution of model (16) supplemented by ICs $\Upsilon (0)=\big ({\textbf{S}}(0),{\textbf{P}}_{\textbf{m}_{1}}(0),{\textbf{H}}_{\textbf{m}_{1}}(0),{\textbf{H}}_{1}(0),{\textbf{H}}_{\textbf{X}}(0),{\textbf{P}}(0),{\textbf{Y}}(0), {\textbf{P}}_{\textbf{T}}(0),{\textbf{T}}_{\textbf{H}\textbf{X}}(0)\big )^{\bar{T}}.$ Furthermore, we utilize ${\textbf{z}}_{1}\vee ..\vee {\textbf{z}}_{\kappa }$ to represent $\max \{{\textbf{z}}_{1}...{\textbf{z}}_{\kappa }\}$ and ${\textbf{z}}\wedge ...\wedge {\textbf{z}}_{\kappa }$ to show $\min \{{\textbf{z}}_{1}...{\textbf{z}}_{\kappa }\}.$ Firstly, we assert an outcome about the existence-uniqueness of a global non-negative solution for system (16).

### Theorem 3.1

*Assume that there is a unique solution* $\Upsilon (\textbf{t})\in \mathbb {R}_{+}^{9}$ *of structure* (16) *on* $[0,\infty )$ *for any starting value* $\Upsilon (0)\in \mathbb {R}_{+}^{9}.$ *It stays in* $\in \mathbb {R}_{+}^{9}$ *having probability* 1 (*a.s*).

### Proof

Here, we overlook the initial portion of the explanation just to display the essential Lyapunov function because it is comparable to Theorem 2.1 in^40^.

 Introducing a $\mathbb {C}^{2}$-functional $\Phi _{0}$ on $\mathbb {R}_{+}^{9}\mapsto \mathbb {R}_{+}$ by

17 $$\begin{aligned} \Phi _{0}(\Upsilon ){} & {} =\Big [\big ({\textbf{S}}-\ell -\ell \ln \frac{\textbf{S}}{\ell }\Big )+({\textbf{P}}_{\textbf{m}_{1}}-1-\ln {\textbf{P}}_{\textbf{m}_{1}})+({\textbf{H}}_{\textbf{m}_{1}}-1-\ln {\textbf{H}}_{\textbf{m}_{1}})+({\textbf{H}}-1-\ln {\textbf{H}})\nonumber \\ {}{} & {} \quad +({\textbf{H}}_{\textbf{X}}-1-\ln {{\textbf{H}}_{\textbf{X}}})+({\textbf{P}}-1-\ln {\textbf{P}})+({\textbf{Y}}-1-\ln {\textbf{Y}})+({\textbf{P}}_{\textbf{T}}-1-\ln {{\textbf{P}}_{\textbf{T}}})\nonumber \\ {}{} & {} \quad +({\textbf{T}}_{\textbf{H}\textbf{X}}-1-\ln {{\textbf{T}}_{\textbf{H}\textbf{X}}})\Big ], \end{aligned}$$

where the value of the non-negative constant $\ell$ will be obtained hereafter. When we implement Itô’s algorithm^52^ to $\Phi _{0}$, we obtain

18 $$\begin{aligned} d\Phi _{0}(\Upsilon ){} & {} =\mathfrak {L}\Phi _{0}(\Upsilon )d\textbf{t}+\wp _{1}(\textbf{S}-\ell )d\mathbb {W}_{1}(\textbf{t})+\wp _{2}({\textbf{P}}_{\textbf{m}_{1}}-1)d\mathbb {W}_{2}(\textbf{t})+\wp _{3}({\textbf{H}}_{\textbf{m}_{1}}-1)d\mathbb {W}_{3}(\textbf{t})\nonumber \\ {}{} & {} \quad +\wp _{4}({\textbf{H}}-1)d\mathbb {W}_{4}(\textbf{t})+\wp _{5}({\textbf{H}}_{\textbf{X}}-1)d\mathbb {W}_{5}(\textbf{t})+\wp _{6}({\textbf{P}}-1)d\mathbb {W}_{6}(\textbf{t})\nonumber \\ {}{} & {} \quad +\wp _{7}({\textbf{Y}}-1)d\mathbb {W}_{7}(\textbf{t})+\wp _{8}({\textbf{P}}_{\textbf{T}}-1)d\mathbb {W}_{8}(\textbf{t})+\wp _{9}({\textbf{T}}_{\textbf{H}\textbf{X}}-1)d\mathbb {W}_{9}(\textbf{t}), \end{aligned}$$

where $\mathfrak {L}\Phi _{0}:\mathbb {R}_{+}^{8}\mapsto \mathbb {R}$ is determined by

19 $$\begin{aligned} \mathfrak {L}\Phi _{0}(\Upsilon ){} & {} =\Big (1-\frac{\ell }{\textbf{S}}\Big )\Big [(1-\textbf{m}_{1}-\textbf{m}_{2})\Lambda +\varphi _{1}{\textbf{P}}_{\textbf{m}_{1}}+\varphi {\textbf{H}}_{\textbf{m}_{1}}+{\Im _{1}}{\textbf{P}}_{\textbf{T}}-(\chi _{\textbf{H}}+\chi _{\textbf{q}}+\eta ){\textbf{S}}\Big ]+\frac{\ell }{2}\wp _{1}^{2}\nonumber \\ {}{} & {} \quad +\Big (1-\frac{1}{{\textbf{P}}_{\textbf{m}_{1}}}\Big )\Big [\textbf{m}_{1}\Lambda -(\varphi _{3}\chi _{\textbf{H}}+\varphi _{1}+\eta ){\textbf{P}}_{\textbf{m}_{1}}\Big ]+\frac{1}{2}\wp _{2}^{2}\nonumber \\ {}{} & {} \quad + \Big (1-\frac{1}{{\textbf{H}}_{\textbf{m}_{1}}}\Big )\Big [\textbf{m}_{2}\Lambda -(\varphi _{2}+\eta +\omega _{1}\chi _{\textbf{q}}){\textbf{H}}_{\textbf{m}_{1}}\Big ]+\frac{1}{2}\wp _{3}^{2}\nonumber \\ {}{} & {} \quad + \Big (1-\frac{1}{{\textbf{H}}}\Big )\Big [\chi _{\textbf{H}}{\textbf{S}}+\varphi _{3}\chi _{\textbf{H}}{\textbf{P}}_{\textbf{m}_{1}}-(\eta +\tau +\varphi _{4}+\varphi _{5}\chi _{\textbf{q}}){\textbf{H}}\Big ]+\frac{1}{2}\wp _{4}^{2}\nonumber \\ {}{} & {} \quad + \Big (1-\frac{1}{{\textbf{H}}_{\textbf{X}}}\Big )\Big [\tau {\textbf{H}}-(\eta +\eta _{1}+\varphi _{7}+\varphi _{6}\chi _{\textbf{q}}){\textbf{H}}_{\textbf{X}}\Big ]+\frac{1}{2}\wp _{5}^{2}\nonumber \\ {}{} & {} \quad +\Big (1-\frac{1}{{\textbf{P}}}\Big )\Big [\chi _{\textbf{q}}{\textbf{S}}+\omega _{1}\chi _{\textbf{q}}{\textbf{H}}_{\textbf{m}_{1}}-(\eta +\eta _{2}+\delta _{3}+\zeta \chi _{\textbf{H}}){\textbf{P}}\Big ]+\frac{1}{2}\wp _{6}^{2}\nonumber \\ {}{} & {} \quad + \Big (1-\frac{1}{\textbf{Y}}\Big )\Big [\zeta \chi _{\textbf{H}}{\textbf{P}}+\varphi _{5}\chi _{\textbf{q}}{\textbf{H}}+\varphi _{6}\chi _{\textbf{q}}{\textbf{H}}_{\textbf{X}}+\sigma \chi _{\textbf{q}}{\textbf{T}}_{\textbf{H}\textbf{X}}-(\eta +\eta _{3}+{\Im _{2}}){\textbf{Y}}\Big ]+\frac{1}{2}\wp _{7}^{2}\nonumber \\ {}{} & {} \quad + \Big (1-\frac{1}{{\textbf{P}}_{\textbf{T}}}\Big )\Big [\delta _{3}{\textbf{P}}-(\eta +{\Im _{1}}){\textbf{P}}_{\textbf{T}}\Big ]+\frac{1}{2}\wp _{8}^{2}\nonumber \\ {}{} & {} \quad + \Big (1-\frac{1}{{\textbf{T}}_{\textbf{H}\textbf{X}}}\Big )\Big [\varphi _{4}\textbf{H}+\varphi _{7}{\textbf{H}}_{\textbf{X}}+{\Im _{2}}{\textbf{Y}}-(\eta +\omega _{1}\chi _{\textbf{q}}){\textbf{T}}_{\textbf{H}\textbf{X}}\Big ]+\frac{1}{2}\wp _{9}^{2}\nonumber \\ {}{} & {} \le \big (\Lambda -\ell (\chi _{\textbf{H}}+\chi _{\textbf{q}}+\eta )+\varphi _{3}\chi _{\textbf{H}}+\varphi _{1}+8\eta +\varphi _{2}+\omega _{1}\chi _{\textbf{q}}+\tau +\varphi _{4}+\varphi _{5}\chi _{\textbf{q}}+\eta _{1}+\varphi _{7}\nonumber \\ {}{} & {} \quad +\varphi _{6}\chi _{\textbf{q}}+\eta _{2}+\delta _{3}+\zeta \chi _{\textbf{H}}+\eta _{3}+{\Im _{2}}+{\Im _{1}}+\omega _{1}\chi _{\textbf{q}}\big ) \nonumber \\ {}{} & {} \quad +\frac{1}{2}\big (\ell \wp _{1}^{2}+\wp _{2}^{2}+\wp _{3}^{2}+\wp _{4}^{2}+\wp _{5}^{2}+\wp _{6}^{2}+\wp _{7}^{2}+\wp _{8}^{2}+\wp _{9}^{2}\big ). \end{aligned}$$

Letting $\ell =\Lambda /(\chi _{\textbf{H}}+\chi _{\textbf{q}}+\eta ).$ As a result, we have

20 $$\begin{aligned} \mathfrak {L}\Phi _{0}(\Upsilon ){} & {} \le \big (\varphi _{3}\chi _{\textbf{H}}+\varphi _{1}+8\eta +\varphi _{2}+\omega _{1}\chi _{\textbf{q}}+\tau +\varphi _{4}+\varphi _{5}\chi _{\textbf{q}}+\eta _{1}+\varphi _{7}\nonumber \\ {}{} & {} \quad +\varphi _{6}\chi _{\textbf{q}}+\eta _{2}+\delta _{3}+\zeta \chi _{\textbf{H}}+\eta _{3}+{\Im _{2}}+{\Im _{1}}+\omega _{1}\chi _{\textbf{q}}\big ) \nonumber \\{} & {} \quad +\frac{1}{2}\Big (\frac{\Lambda }{\chi _{\textbf{H}}+\chi _{\textbf{q}}+\eta }\wp _{1}^{2}+\wp _{2}^{2}+\wp _{3}^{2}+\wp _{4}^{2}+\wp _{5}^{2}+\wp _{6}^{2}+\wp _{7}^{2}+\wp _{8}^{2}+\wp _{9}^{2}\Big )\nonumber \\ {}{} & {} :=\mathcal {K}, \end{aligned}$$

where the constant $\mathcal {K}$ is non-negative. According to^40^, we similarly exclude the remaining portion of the explanation. This completes the proof. $\square$

### Stationary distribution

Our primary concern with the stochastic outbreak framework is the virus’s permanence. In this portion, we employ a novel method to demonstrate that structure (16) has a unique ESD, depending on the hypothesis of Khasminskii^53^. 

By developing appropriate Lyapunov functions, we will show adequate conditions for the development of a unique ESD. A key component of our major result’s explanation is the lemma that follows. Assume that $\mathcal {Y}(\textbf{t})$ is an ordinary time-homogeneous Markov phenomenon with $\mathbb {R}^{\mathbb {S}}$. Its stochastic DE is as follows:

21 $$\begin{aligned} d\mathcal {Y}(\textbf{t})=\textbf{b}(\textbf{y})(\textbf{t})+\sum \limits _{\textbf{w}=1}^{\textbf{s}}{\Im _{1}}_{\textbf{w}}(\mathcal {Y})d{\mathbb {W}}_{\textbf{w}}(\textbf{t}), \end{aligned}$$

and the diffusion matrix is $\textbf{X}(\varkappa )=\big ({a}_{\jmath \kappa }(\varkappa )\big )_{\jmath \ge 1, \textbf{s}\ge \kappa },~{a}_{\jmath \kappa }(\varkappa )=\sum \limits _{\textbf{w}=1}^{l}{\Im _{1}}_{\textbf{w}}^{\jmath }(\varkappa ){\Im _{1}}_{\textbf{w}}^{\kappa }(\varkappa ).$ Consider the differential operator L connected to (24) as follows:

22 $$\begin{aligned} \mathfrak {L}=\sum \limits _{\jmath =1}^{\textbf{s}}{\textbf{b}}_{\jmath }(\varkappa )\frac{\partial }{\partial {\varkappa }_{\jmath }}+\frac{1}{2}\sum \limits _{\jmath ,\kappa =1}^{\textbf{s}}\textbf{V}_{\jmath \kappa }(\varkappa )\frac{\partial ^{2}}{\partial {\varkappa }_{\jmath }\partial {\varkappa }_{\kappa }}. \end{aligned}$$

#### Lemma 3.1

(^53^) *Let us suppose the subsequent characteristics of a bounded open region* $\mathcal {D}_{\varphi }\in \mathbb {R}^{\mathbb {S}}$ *with a regular boundary*:

 ($\mathcal {H}_{1}$):  *In the region* $\mathcal {D}_{\varphi }\in \mathbb {R}^{\mathbb {S}}$ *and some neighborhood therefore, the least significant eigenvalue of the diffusion matrix* $\textbf{V}(\varkappa )$ *is bounded away from zero*. 

($\mathcal {H}_{2}$): $\exists$ *a positive* $\mathbb {C}^{2}$-*function* $\Phi$ *so that* $\mathfrak {L}\Phi$ *is negative for all* $\mathbb {R}^{\mathbb {S}}\setminus \mathcal {D}_{\varphi }.$

*Then the Markov procedure* $\mathcal {Y}(\textbf{t})$ *has a stationary distribution* $\pi (.\,).$ *Also, consider* $\mathcal {F}(\varkappa )$ *be a mapping which is positive in regard to the measure* $\pi ,~~\forall ~\varkappa \in \mathbb {R}^{\mathbb {S}},$ *ones obtain*

23 $$\begin{aligned} \mathbb {P}\Big \{\lim _{\textbf{T}\mapsto \infty }\frac{1}{\textbf{T}}\int \limits _{0}^{\textbf{T}}\mathcal {F}\big (\mathcal {Y}(\textbf{t})\big )d\textbf{t}=\int \limits _{\mathbb {R}^{\mathbb {S}}}\mathcal {F}(\varkappa )\zeta _{2}(d\varkappa )\Big \}=1. \end{aligned}$$

To begin with, we establish a few concepts for ease of use in later explanations. By resolving the subsequent (24) as

24 $$\begin{aligned} {\left\{ \begin{array}{ll} (1-\textbf{m}_{1}-\textbf{m}_{2})\Lambda +\varphi _{1}\tilde{{\textbf{P}}_{\textbf{m}_{1}}}+\varphi _{2}\tilde{{\textbf{H}}_{\textbf{m}_{1}}}=\Big (\chi _{\textbf{H}}+\chi _{\textbf{q}}+\eta +\frac{\wp _{1}^{2}}{2}\Big )\tilde{{\textbf{S}}},\\ \textbf{m}_{1}\Lambda =\Big (\varphi _{3}\chi _{\textbf{H}}+\varphi _{1}+\eta +\frac{\wp _{2}^{2}}{2}\Big )\tilde{{\textbf{P}}_{\textbf{m}_{1}}},\\ \textbf{m}_{2}\Lambda =\Big (\varphi _{2}+\eta +\omega _{1}\chi _{\textbf{q}}+\frac{\wp _{3}^{2}}{2}\Big )\tilde{{\textbf{H}}_{\textbf{P}}},\\ \chi _{\textbf{q}}\tilde{\textbf{S}}+\omega _{1}\chi _{\textbf{q}}\tilde{{\textbf{H}}_{\textbf{P}}}=\Big (\eta +\eta _{2}+\delta _{3}+\zeta \chi _{\textbf{H}}+\frac{\wp _{6}^{2}}{2}\Big ) \tilde{\textbf{P}},\\ \delta _{3}\tilde{\textbf{P}}=\Big (\eta +{\Im _{1}}+\frac{\wp _{8}^{2}}{2}\Big ) \tilde{{\textbf{P}}_{\textbf{T}}}, \end{array}\right. } \end{aligned}$$

we find

25 $$\begin{aligned}{} & {} \tilde{\textbf{S}}=\frac{\Lambda (1-\textbf{m}_{1}-\textbf{m}_{2})\rho _{3}\rho _{2}+\varphi _{1}\textbf{m}_{1}\Lambda \rho _{3}+\varphi _{2}\textbf{m}_{2}\chi \rho _{3}}{\rho _{1}\rho _{2}\rho _{3}},~~~~~ \tilde{{\textbf{P}}_{\textbf{m}_{1}}}=\frac{\textbf{m}_{1}\Lambda }{\rho _{2}},~~~\tilde{{\textbf{H}}_{\textbf{P}}}=\frac{\textbf{m}_{2}\Lambda }{\rho _{3}},\nonumber \\ {}{} & {} \tilde{\textbf{P}}=\bigg \{\frac{\chi _{\textbf{q}}\big (\Lambda (1-\textbf{m}_{1}-\textbf{m}_{2})\rho _{3}\rho _{2}+\varphi _{1}\textbf{m}_{1}\Lambda \rho _{3}+\varphi _{2}\textbf{m}_{2}\chi \rho _{3}\big )+\Lambda \omega _{1} \textbf{m}_{2}\chi _{\textbf{q}}\rho _{1}\rho _{2}}{\rho _{1}\rho _{2}\rho _{3}\rho _{4}}\bigg \},\nonumber \\ {}{} & {} \tilde{{\textbf{P}}_{\textbf{T}}}=\frac{\delta _{3}}{\rho _{5}}\bigg \{\frac{\chi _{\textbf{q}}\big (\Lambda (1-\textbf{m}_{1}-\textbf{m}_{2})\rho _{3}\rho _{2}+\varphi _{1}\textbf{m}_{1}\Lambda \rho _{3}+\varphi _{2}\textbf{m}_{2}\chi \rho _{3}\big )+\Lambda \omega _{1} \textbf{m}_{2}\chi _{\textbf{q}}\rho _{1}\rho _{2}}{\rho _{1}\rho _{2}\rho _{3}\rho _{4}}\bigg \}, \end{aligned}$$

where

$${\left\{ \begin{array}{ll}\rho _{1}=\chi _{\textbf{H}}+\chi _{\textbf{q}}+\eta +\frac{\wp _{1}^{2}}{2},\\ \rho _{2}=\varphi _{3}\chi _{\textbf{H}}+\varphi _{1}+\eta +\frac{\wp _{2}^{2}}{2},\\ \rho _{3}=\varphi _{2}+\eta +\omega _{1}\chi _{\textbf{q}}+\frac{\wp _{3}^{2}}{2},\\ \rho _{4}=\eta +\eta _{2}+\delta _{3}+\zeta \chi _{\textbf{H}}+\frac{\wp _{6}^{2}}{2},\\ \rho _{5}=\eta +{\Im _{1}}+\frac{\wp _{8}^{2}}{2}.\end{array}\right. }$$

Afterwards, by addressing the subsequent formula’s:

26 $$\begin{aligned} {\left\{ \begin{array}{ll} \chi _{\textbf{H}}\tilde{\textbf{S}}+\varphi _{3}\chi _{\textbf{H}}\tilde{{{\textbf{P}}_{\textbf{m}_{1}}}}=\Big (\eta +\tau +\varphi _{4}+\varphi _{5}\chi _{\textbf{q}}+\frac{\wp _{4}^{2}}{2}\Big )\tilde{{\textbf{H}}},\\ \tau \tilde{\textbf{H}}=\Big (\eta +\eta _{1}+\varphi _{7}+\varphi _{6}\chi _{\textbf{q}}+\frac{\wp _{5}^{2}}{2}\Big )\tilde{{\textbf{H}}_{\textbf{X}}},\\ \zeta \chi _{\textbf{H}}\tilde{\textbf{P}}+\varphi _{5}\chi _{\textbf{q}}{\textbf{H}}+\varphi _{6}\chi _{\textbf{q}}\tilde{{\textbf{H}}_{\textbf{X}}}=\Big (\eta +\eta _{3}+{\Im _{2}}+\frac{\wp _{7}^{2}}{2}\Big )\tilde{\textbf{Y}},\\ \varphi _{4}\tilde{{\textbf{H}}}+\varphi _{7}\tilde{{\textbf{H}}_{\textbf{X}}}+{\Im _{2}}\tilde{\textbf{Y}}=\Big (\eta +\sigma \chi _{\textbf{q}}+\frac{\wp _{9}^{2}}{2}\Big )\tilde{{\textbf{T}}_{\textbf{H}\textbf{X}}},\end{array}\right. } \end{aligned}$$

which lead us

27 $$\begin{aligned} {\left\{ \begin{array}{ll} \tilde{{\textbf{H}}}=\frac{1}{\rho _{6}}\bigg \{\frac{\Lambda (1-\textbf{m}_{1}-\textbf{m}_{2})\rho _{3}\rho _{2}+\varphi _{1}\textbf{m}_{1}\Lambda \rho _{3}+\varphi _{2}\textbf{m}_{2}\chi \rho _{3}+\textbf{m}_{1}\Lambda \rho _{1}\rho _{3}}{\rho _{1}\rho _{2}\rho _{3}}\bigg \},\\ \tilde{{\textbf{H}}_{\textbf{X}}}=\frac{\tau }{\rho _{6}\rho _{7}}\bigg \{\frac{\Lambda (1-\textbf{m}_{1}-\textbf{m}_{2})\rho _{3}\rho _{2}+\varphi _{1}\textbf{m}_{1}\Lambda \rho _{3}+\varphi _{2}\textbf{m}_{2}\chi \rho _{3}+\textbf{m}_{1}\Lambda \rho _{1}\rho _{3}}{\rho _{1}\rho _{2}\rho _{3}}\bigg \},\\ \tilde{{\textbf{Y}}}=\frac{\zeta \chi _{\textbf{H}}\chi _{\textbf{q}}+\varphi _{5}\chi _{\textbf{q}}+\varphi _{6}\chi _{\textbf{q}}\tau }{\rho _{6}\rho _{7}\rho _{8}}\times \bigg \{\frac{\Lambda (1-\textbf{m}_{1}-\textbf{m}_{2})\rho _{3}\rho _{2}+\varphi _{1}\textbf{m}_{1}\Lambda \rho _{3}+\varphi _{2}\textbf{m}_{2}\chi \rho _{3}+\textbf{m}_{1}\Lambda \rho _{1}\rho _{3}}{\rho _{1}\rho _{2}\rho _{3}}\bigg \},\\ \tilde{{\textbf{T}}_{\textbf{H}\textbf{X}}}=\frac{1}{\rho _{9}}\bigg \{\frac{\varphi _{4}\rho _{7}\rho _{8}+\varphi _{7}\tau \rho _{8}+{\Im _{2}}(\zeta \chi _{\textbf{H}}\chi _{\textbf{q}}+\varphi _{5}\chi _{\textbf{q}}+\varphi _{6}\chi _{\textbf{q}}\tau )}{\rho _{6}\rho _{7}\rho _{8}}\bigg \}\times \bigg \{\frac{\Lambda (1-\textbf{m}_{1}-\textbf{m}_{2})\rho _{3}\rho _{2}+\varphi _{1}\textbf{m}_{1}\Lambda \rho _{3}+\varphi _{2}\textbf{m}_{2}\chi \rho _{3}+\textbf{m}_{1}\Lambda \rho _{1}\rho _{3}}{\rho _{1}\rho _{2}\rho _{3}}\bigg \},\\ \end{array}\right. } \end{aligned}$$

where

$$\rho _{6}=\eta +\tau +\varphi _{4}+\varphi _{5}\chi _{\textbf{q}}+\frac{\wp _{4}^{2}}{2},$$

$$\rho _{7}=\eta +\eta _{1}+\varphi _{7}+\varphi _{6}\chi _{\textbf{q}}+\frac{\wp _{5}^{2}}{2},$$

$$\rho _{8}=\eta +\eta _{3}+{\Im _{2}}+\frac{\wp _{7}^{2}}{2}$$,

$$\rho _{9}=\eta +\sigma \chi _{\textbf{q}}+\frac{\wp _{9}^{2}}{2}.$$

Introduce

28 $$\begin{aligned} \mathbb {R}_{0}^{\mathbb {S}}=\frac{\chi _{\textbf{H}}\widetilde{\textbf{S}}+\varphi _{3}\chi _{\textbf{H}}\widetilde{{\textbf{P}}_{\textbf{m}_{1}}}}{\eta +\tau +\varphi _{4}+\varphi _{5}\chi _{\textbf{q}}+\frac{\wp _{4}^{2}}{2}}. \end{aligned}$$

#### Theorem 3.2

*If we suppose that* $\mathbb {R}_{0}^{\mathbb {S}}>1$, *then structure* (16) *permits a unique ESD*, $\pi (.\,).$

#### Proof

It is necessary to validate assumptions $(\mathcal {H}_{1})$ and $(\mathcal {H}_{2})$ in Lemma 3.1 for the purpose of establishing Theorem 3.2.

 To begin with, we create an appropriate Lyapunov function $\Phi$ and identify a closed set $\mathcal {D}_{\varphi }\in \mathbb {R}_{+}^{8}$ that ensures $\sup \limits _{\varkappa \in \mathbb {R}_{+}^{8}\setminus \mathcal {D}_{\varphi }}\mathfrak {L}\Phi (\varkappa )$ is negative in order to ensure the efficacy of $(\mathcal {H}_{2})$ in Lemma 3.1.

For this, let us suppose

$$\begin{aligned} \widetilde{{\textbf{S}}}=\frac{{\textbf{S}}}{\overline{{\textbf{S}}}},~~~\widetilde{{{\textbf{P}}}_{\textbf{m}_{1}}}=\frac{{\textbf{P}}_{\textbf{m}_{1}}}{\overline{{\textbf{P}}_{\textbf{m}_{1}}}},~~~\widetilde{{\textbf{H}}_{\textbf{m}_{1}}}=\frac{{\textbf{H}}_{\textbf{m}_{1}}}{\overline{{\textbf{H}}_{\textbf{m}_{1}}}},~~~\widetilde{{\textbf{P}}}=\frac{\textbf{P}}{\overline{{\textbf{P}}}},~~~\widetilde{{\textbf{P}}_{\textbf{T}\textbf{X}}}=\frac{{\textbf{P}}_{\textbf{T}\textbf{X}}}{\overline{{\textbf{P}}_{\textbf{T}\textbf{X}}}}. \end{aligned}$$

Implementing Itô’s technique to $-\ln {\textbf{S}},$ we find

29 $$\begin{aligned} \mathfrak {L}(-\ln {\textbf{S}}){} & {} \le -\frac{(1-\textbf{m}_{1}-\textbf{m}_{2})\Lambda }{{\textbf{S}}}-\frac{\varphi _{1}{\textbf{P}}_{\textbf{m}_{1}}}{\textbf{S}}-\frac{\varphi _{2}{\textbf{H}}_{\textbf{m}_{1}}}{\textbf{S}}-\frac{{\Im _{1}}{\textbf{P}}_{\textbf{T}}}{\textbf{S}}+\chi _{\textbf{H}}+\chi _{\textbf{q}}+\eta +\frac{\wp _{1}^{2}}{2}\nonumber \\ {}{} & {} =-\frac{(1-\textbf{m}_{1}-\textbf{m}_{2})\Lambda }{\overline{\textbf{S}}\tilde{\textbf{S}}}-\frac{\varphi _{1}\overline{{\textbf{P}}_{\textbf{m}_{1}}}\tilde{{\textbf{P}}_{\textbf{m}_{1}}}}{\overline{\textbf{S}}\tilde{\textbf{S}}}-\frac{\varphi _{2}\overline{{\textbf{H}}_{\textbf{m}_{1}}}\tilde{{\textbf{H}}_{\textbf{m}_{1}}}}{\overline{\textbf{S}}\tilde{\textbf{S}}}-\frac{{\Im _{1}}\overline{{\textbf{P}}_{\textbf{T}}}\tilde{{\textbf{P}}_{\textbf{T}}}}{\overline{\textbf{S}}\tilde{\textbf{S}}}+\chi _{\textbf{H}}+\chi _{\textbf{q}}+\eta +\frac{\wp _{1}^{2}}{2}\nonumber \\ {}{} & {} =-\frac{(1-\textbf{m}_{1}-\textbf{m}_{2})\Lambda }{\overline{\textbf{S}}}-\frac{\varphi _{1}\overline{{\textbf{P}}_{\textbf{m}_{1}}}}{\overline{\textbf{S}}}-\frac{\varphi _{2}\overline{{\textbf{H}}_{\textbf{m}_{1}}}\tilde{{\textbf{H}}_{\textbf{m}_{1}}}}{\overline{\textbf{S}}\tilde{\textbf{S}}}-\frac{{\Im _{1}}\overline{{\textbf{P}}_{\textbf{T}}}\tilde{{\textbf{P}}_{\textbf{T}}}}{\overline{\textbf{S}}\tilde{\textbf{S}}}+\chi _{\textbf{H}}+\chi _{\textbf{q}}+\eta +\frac{\wp _{1}^{2}}{2}\nonumber \\ {}{} & {} \quad -\frac{(1-\textbf{m}_{1}-\textbf{m}_{2})\Lambda }{\overline{\textbf{S}}}\Big (\frac{1}{\tilde{\textbf{S}}}-1\Big )-\frac{\varphi _{1}\overline{{\textbf{P}}_{\textbf{m}_{1}}}}{\overline{\textbf{S}}}\Big (\frac{\tilde{{\textbf{P}}_{\textbf{m}_{1}}}}{\tilde{\textbf{S}}}-1\Big )-\frac{\varphi _{2}\overline{{\textbf{H}}_{\textbf{m}_{1}}}}{\overline{\textbf{S}}}\Big (\frac{\tilde{{\textbf{H}}_{\textbf{m}_{1}}}}{\tilde{\textbf{S}}}-1\Big )-\frac{{\Im _{1}}\overline{{\textbf{P}}_{\textbf{T}}}}{\overline{\textbf{S}}}\Big (\frac{\tilde{{\textbf{P}}_{\textbf{T}}}}{\tilde{\textbf{S}}}-1\Big ).\nonumber \\ \end{aligned}$$

Applying the variant $\ln \varkappa \le \varkappa -1~(\forall ~\varkappa >0),$ we have $\ln \frac{1}{\widetilde{\textbf{S}}}\le \frac{1-\widetilde{\textbf{S}}}{\widetilde{\textbf{S}}}.$

Again, considering (24), we have

30 $$\begin{aligned}{} & {} \mathfrak {L}(-\ln \textbf{S})\le -\frac{(1-\textbf{m}_{1}-\textbf{m}_{2})\Lambda }{\overline{\textbf{S}}}\ln \frac{1}{\textbf{S}}-\frac{\varphi _{1}\overline{{\textbf{P}}_{\textbf{m}_{1}}}}{\overline{\textbf{S}}}\ln \frac{\widetilde{{\textbf{P}}_{\textbf{m}_{1}}}}{\widetilde{\textbf{S}}}-\frac{\varphi _{2}\overline{{\textbf{H}}_{\textbf{m}_{1}}}}{\overline{\textbf{S}}}\ln \frac{\widetilde{\textbf{S}}}{\widetilde{{\textbf{H}}_{\textbf{m}_{1}}}}-\frac{{\Im _{1}}\overline{{\textbf{P}}_{\textbf{T}}}}{\overline{\textbf{S}}}\ln \frac{\widetilde{\textbf{S}}}{\widetilde{{\textbf{P}}_{\textbf{T}}}}\nonumber \\{} & {} \quad -\frac{(1-\textbf{m}_{1}-\textbf{m}_{2})\Lambda }{\overline{\textbf{S}}}\Big (\frac{1}{\tilde{\textbf{S}}}-1\Big )-\frac{\varphi _{1}\overline{{\textbf{P}}_{\textbf{m}_{1}}}}{\overline{\textbf{S}}}\Big (\frac{\tilde{{\textbf{P}}_{\textbf{m}_{1}}}}{\tilde{\textbf{S}}}-1\Big )-\frac{\varphi _{2}\overline{{\textbf{H}}_{\textbf{m}_{1}}}}{\overline{\textbf{S}}}\Big (\frac{\tilde{{\textbf{H}}_{\textbf{m}_{1}}}}{\tilde{\textbf{S}}}-1\Big )-\frac{{\Im _{1}}\overline{{\textbf{P}}_{\textbf{T}}}}{\overline{\textbf{S}}}\Big (\frac{\tilde{{\textbf{P}}_{\textbf{T}}}}{\tilde{\textbf{S}}}-1\Big )\nonumber \\{} & {} =\frac{(1-\textbf{m}_{1}-\textbf{m}_{2})\Lambda +\varphi _{1}\overline{\textbf{P}}_{\textbf{m}_{1}}+\varphi _{2}\overline{\textbf{H}}_{\textbf{m}_{1}}+{\Im _{1}}\overline{\textbf{P}}_{\textbf{T}}}{\overline{\textbf{S}}}\ln \widetilde{\textbf{S}}-\frac{\varphi _{1}\overline{{\textbf{P}}_{\textbf{m}_{1}}}}{\overline{\textbf{S}}}\ln \widetilde{{\textbf{P}}_{\textbf{m}_{1}}}-\frac{\varphi _{2}\overline{{\textbf{H}}_{\textbf{m}_{1}}}}{\overline{\textbf{S}}}\ln \widetilde{{\textbf{H}}_{\textbf{m}_{1}}}-\frac{{\Im _{1}}\overline{{\textbf{P}}_{\textbf{T}}}}{\overline{\textbf{S}}}\ln \widetilde{{\textbf{P}}_{\textbf{T}}}. \end{aligned}$$

Adopting the similar technique to $-\ln {\textbf{P}}_{\textbf{m}_{1}},~-\ln {\textbf{H}}_{\textbf{m}_{1}},~\ln {\textbf{P}}$ and $\ln {{\textbf{P}}_{\textbf{T}}},$ respectively, we have

31 $$\begin{aligned}{} & {} \mathfrak {L}(-\ln {\textbf{P}}_{\textbf{m}_{1}})\le \frac{\textbf{m}_{1}\Lambda }{\overline{{\textbf{P}}_{\textbf{m}_{1}}}}\ln \widetilde{{{\textbf{P}}_{\textbf{m}_{1}}}}-\frac{\delta _{1}\overline{\textbf{H}}}{\overline{{\textbf{P}}_{\textbf{m}_{1}}}}\ln \widetilde{\textbf{H}}-\frac{\delta _{1}\tilde{\omega _{1}}\overline{{\textbf{H}}_{\textbf{X}}}}{\overline{{\textbf{P}}_{\textbf{m}_{1}}}}\ln \widetilde{{\textbf{H}}_{\textbf{X}}}-\frac{\delta _{1}{\Im _{2}}\overline{{\textbf{Y}}}}{\overline{{\textbf{P}}_{\textbf{m}_{1}}}}\ln \widetilde{{\textbf{Y}}},\nonumber \\{} & {} \mathfrak {L}(-\ln {{\textbf{H}}_{\textbf{m}_{1}}})\le \frac{\textbf{m}_{2}\Lambda }{\overline{{\textbf{H}}_{\textbf{m}_{1}}}}\ln \widetilde{{\textbf{H}}_{\textbf{m}_{1}}}-\frac{\delta _{2}\overline{\textbf{P}}}{\overline{{\textbf{H}}_{\textbf{m}_{1}}}}\ln \widetilde{\textbf{P}}-\frac{\delta _{2}\omega \overline{{\textbf{Y}}}}{\overline{{\textbf{H}}_{\textbf{m}_{1}}}}\ln \widetilde{{\textbf{Y}}},\nonumber \\{} & {} \mathfrak {L}(-\ln {\textbf{P}})\le \frac{\chi _{\textbf{q}}}{\overline{{\textbf{P}}}}\ln \widetilde{\textbf{P}}+\frac{-\omega _{1}\chi _{\textbf{q}}\overline{{\textbf{H}}_{\textbf{m}_{1}}}}{\overline{{\textbf{P}}}}\ln \widetilde{{\textbf{H}}_{\textbf{m}_{1}}},\nonumber \\{} & {} \mathfrak {L}(-\ln {{\textbf{P}}_{\textbf{T}}})\le \frac{\delta _{3}\overline{\textbf{P}}}{\overline{{\textbf{P}}_{\textbf{T}}}}\ln \widetilde{\textbf{P}}. \end{aligned}$$

Now, introducing a $\mathbb {C}^{2}$-mapping $\Phi _{1}$ as

$$\begin{aligned} \Phi _{1}(\Upsilon )=-\ln \textbf{S}-{a_{1}}\ln {\textbf{P}}_{\textbf{m}_{1}}-{a_{2}}\ln {\textbf{H}}_{\textbf{m}_{1}}-{a_{3}}\ln {\textbf{P}}-{a_{4}}\ln {{\textbf{P}}_{\textbf{T}}}, \end{aligned}$$

so that

32 $$\begin{aligned} {\left\{ \begin{array}{ll} {a_{1}}\frac{\textbf{m}_{1}\Lambda }{\overline{{\textbf{P}}_{\textbf{m}_{1}}}}-\frac{\varphi _{1}\overline{{\textbf{P}}_{\textbf{m}_{1}}}}{\overline{\textbf{S}}}=0,\\ {a_{2}}\frac{\textbf{m}_{2}\Lambda }{\overline{{\textbf{H}}_{\textbf{m}_{1}}}}-\frac{\varphi _{2}\overline{{\textbf{H}}_{\textbf{m}_{1}}}}{\overline{\textbf{S}}}-{a_{3}}\frac{\omega _{1}\chi _{\textbf{q}}\overline{{\textbf{H}}_{\textbf{m}_{1}}}}{\overline{\textbf{P}}}=0,\\ {a_{3}}\frac{\chi _{\textbf{q}}}{\overline{\textbf{P}}}-{a_{4}}\frac{\delta _{2}\overline{\textbf{P}}}{\overline{{\textbf{H}}_{\textbf{m}_{1}}}}=0,\\ {a_{4}}-\frac{{\Im _{1}}\overline{{\textbf{P}}_{\textbf{T}}}}{\overline{\textbf{S}}}=0, \end{array}\right. } \end{aligned}$$

where

$$\begin{aligned} a_{1}=\frac{\varphi _{1}\overline{{\textbf{P}}_{\textbf{m}_{1}}^{2}}}{\textbf{m}_{1}\Lambda \overline{\textbf{S}}},~~~{a_{2}}=\frac{\overline{{\textbf{H}}_{\textbf{m}_{1}}}\big (\varphi _{2}\overline{{\textbf{H}}}_{\textbf{m}_{1}}+\omega _{1}{\Im _{1}}\delta _{2}\overline{\textbf{P}}\overline{{\textbf{P}}_{\textbf{T}}}\big )}{\textbf{m}_{2}\Lambda \overline{\textbf{S}}},~~~{a_{3}}=\frac{{\Im _{1}}\delta _{2}\overline{\textbf{P}}^{2}\overline{{\textbf{P}}_{\textbf{T}}}}{\chi _{\textbf{q}}\overline{{\textbf{H}}_{\textbf{m}_{1}}}\overline{\textbf{S}}},~~~~{a_{4}}=\frac{{\Im _{1}}\overline{{\textbf{P}}_{\textbf{T}}}}{\overline{\textbf{S}}}. \end{aligned}$$

Implementing the Itô’s technique to $\Phi _{1}$ and considering (30)-(32), we have

33 $$\begin{aligned} \mathfrak {L}\Phi _{1}{} & {} \le \frac{(1-\textbf{m}_{1}-\textbf{m}_{2})\Lambda +\varphi _{1}\overline{{\textbf{P}}_{\textbf{m}_{1}}}+\varphi _{2}\overline{\textbf{H}}_{\textbf{m}_{1}}+{\Im _{1}}\overline{\textbf{P}}_{\textbf{T}}}{\overline{\textbf{S}}}\ln \widetilde{\textbf{S}}+\Big ({a_{2}}\frac{\textbf{m}_{1}\Lambda }{\overline{{\textbf{P}}_{\textbf{m}_{1}}}}-\frac{\varphi _{1}\overline{{\textbf{P}}_{\textbf{m}_{1}}}}{\overline{\textbf{S}}}\Big )\ln \widetilde{{\textbf{P}}_{\textbf{P}}}\nonumber \\ {}{} & {} \quad +\Big ({a_{3}}\frac{\textbf{m}_{2}\Lambda }{\overline{{\textbf{H}}}_{\textbf{m}_{1}}}-\frac{\varphi _{2}\overline{{\textbf{H}}_{\textbf{m}_{1}}}}{\overline{\textbf{S}}}-{a_{4}}\frac{\omega _{1}\chi _{\textbf{q}}\overline{{\textbf{H}}_{\textbf{m}_{1}}}}{\overline{\textbf{P}}}\Big )\ln \widetilde{{{\textbf{H}}}_{\textbf{m}_{1}}}+\Big ({a_{4}}\frac{\chi _{\textbf{q}}\overline{\textbf{P}}}{\overline{{\textbf{P}}_{\textbf{T}}}}-\frac{\delta _{2}\omega \overline{\textbf{Y}}}{\overline{{\textbf{H}}_{\textbf{m}_{1}}}}\Big )\ln \widetilde{\textbf{P}}+\frac{\delta _{3}\overline{\textbf{P}}}{\overline{{\textbf{P}}_{\textbf{T}}}}\widetilde{{\textbf{P}}_{\textbf{T}}}. \end{aligned}$$

Then, we describe a $\mathbb {C}^{2}$-function $\Phi _{2}$ as

34 $$\begin{aligned} \Phi _{2}(\Upsilon )=-\ln {{\textbf{P}}_{\textbf{m}_{1}}}-{\textbf{b}_{1}}\ln {\textbf{S}}-{\textbf{b}_{2}}\ln {{\textbf{H}}_{\textbf{m}_{1}}}-{\textbf{b}_{3}}\ln {{\textbf{P}}}-{\textbf{b}_{4}}\ln {{\textbf{P}}_{\textbf{T}}}, \end{aligned}$$

which leads to

35 $$\begin{aligned} {\left\{ \begin{array}{ll} {\textbf{b}_{1}}\frac{(1-\textbf{m}_{1}-\textbf{m}_{2})\Lambda }{\overline{\textbf{S}}}+{\textbf{b}_{2}}\frac{\chi _{\textbf{q}}\overline{\textbf{S}}}{\overline{\textbf{H}}}=0,\\ {\textbf{b}_{2}}\frac{\textbf{m}_{2}\Lambda }{\overline{{\textbf{H}}_{\textbf{m}_{1}}}}-\textbf{b}_{3}\frac{\varphi _{2}\overline{{\textbf{H}}_{\textbf{P}}}}{\overline{\textbf{S}}}=0,\\ -\textbf{b}_{3}\frac{\varphi _{3}\chi _{\textbf{H}}}{\overline{\textbf{H}}}+\frac{\textbf{m}_{1}\Lambda }{\overline{\textbf{P}}}-\textbf{b}_{4}\frac{\varphi _{1}\overline{{\textbf{P}}_{\textbf{P}}}}{\overline{\textbf{S}}}=0,\\ -\textbf{b}_{4}\frac{{\Im _{1}}\overline{{\textbf{P}}_{\textbf{T}}}}{\overline{\textbf{S}}}+\frac{\delta _{3}\overline{\textbf{P}}}{\overline{{\textbf{P}}_{\textbf{T}}}}=0, \end{array}\right. } \end{aligned}$$

where

$$\begin{aligned} \textbf{b}_{1}=\frac{\varphi _{2}\overline{{\textbf{H}}_{\textbf{P}}}^{2}\overline{\textbf{S}}^{2}\chi _{\textbf{q}}}{\Lambda ^{2}\textbf{m}_{2}(1-\textbf{m}_{1}-\textbf{m}_{2})\overline{\textbf{H}}}\bigg \{\frac{\textbf{m}_{1}\Lambda {\Im _{1}}\overline{{\textbf{H}}}\overline{{\textbf{P}}_{\textbf{T}}}^{2}-\delta _{3}\varphi _{1}\overline{{\textbf{H}}}\overline{{\textbf{P}}}\overline{{\textbf{P}}_{\textbf{m}_{1}}}^{2}}{{\Im _{1}}\varphi _{3}\chi _{\textbf{H}}\overline{{\textbf{P}}_{\textbf{m}_{1}}}^{2}\overline{{\textbf{P}}_{\textbf{T}}}^{2}}\bigg \}, \end{aligned}$$

$$\begin{aligned} \textbf{b}_{2}=\frac{\varphi _{2}\overline{{\textbf{H}}_{\textbf{P}}}^{2}}{\Lambda \textbf{m}_{2}(1-\textbf{m}_{1}-\textbf{m}_{2})\overline{\textbf{S}}}\bigg \{\frac{\textbf{m}_{1}\Lambda {\Im _{1}}\overline{{\textbf{H}}}\overline{{\textbf{P}}_{\textbf{T}}}^{2}-\delta _{3}\varphi _{1}\overline{{\textbf{H}}}\overline{{\textbf{P}}}\overline{{\textbf{P}}_{\textbf{m}_{1}}}^{2}}{{\Im _{1}}\varphi _{3}\chi _{\textbf{H}}\overline{{\textbf{P}}_{\textbf{m}_{1}}}^{2}\overline{{\textbf{P}}_{\textbf{T}}}^{2}}\bigg \}, \\ \textbf{b}_{3}=\frac{\textbf{m}_{1}\Lambda {\Im _{1}}\overline{{\textbf{H}}}\overline{{\textbf{P}}_{\textbf{T}}}^{2}-\delta _{3}\varphi _{1}\overline{{\textbf{H}}}\overline{{\textbf{P}}}\overline{{\textbf{P}}_{\textbf{m}_{1}}}^{2}}{{\Im _{1}}\varphi _{3}\chi _{\textbf{H}}\overline{{\textbf{P}}_{\textbf{m}_{1}}}^{2}\overline{{\textbf{P}}_{\textbf{T}}}^{2}},~~~~~~~~~~~~\textbf{b}_{4}=\frac{\delta _{3} \textbf{m}_{1}\overline{\textbf{S}}}{{\Im _{1}}\overline{{\textbf{P}}}_{\textbf{T}}}. \end{aligned}$$

Implementing the Itô’s technique to $\Phi _{2}$ and considering (33)-(35), we have

36 $$\begin{aligned} \mathfrak {L}\Phi _{2}(\Upsilon )\le \bigg (\frac{\textbf{m}_{1}\Lambda }{\overline{{\textbf{P}}_{\textbf{m}_{1}}}}-\mathbf {\textbf{b}_{1}}\frac{\varphi _{1}\overline{{\textbf{P}}_{\textbf{m}_{1}}}}{\overline{\textbf{S}}}-\textbf{b}_{3}\frac{\varphi _{3}\chi _{\textbf{H}}\overline{{\textbf{P}}_{\textbf{m}_{1}}}}{\overline{{\textbf{H}}}}\bigg )\ln \widetilde{{\textbf{P}}_{\textbf{m}_{1}}}+\textbf{b}_{1}\delta _{1}{\textbf{H}}+\delta _{1}\tilde{\omega _{1}}{\textbf{H}}_{\textbf{X}}. \end{aligned}$$

Again, we describe a $\mathbb {C}^{2}$-function $\Phi _{3}$ as

37 $$\begin{aligned} \Phi _{3}(\Lambda )=-\ln {{\textbf{H}}_{\textbf{m}_{1}}}-{c_{1}}\ln {{\textbf{P}}_{\textbf{m}_{1}}}-{c_{2}}\ln {\textbf{S}}-{c_{3}}\ln {{\textbf{P}}}-{c_{4}}\ln {{\textbf{P}}_{\textbf{T}}}, \end{aligned}$$

which leads to

38 $$\begin{aligned} {\left\{ \begin{array}{ll} {\textbf{c}_{1}}\frac{\textbf{m}_{1}\Lambda }{\overline{{\textbf{P}}_{\textbf{m}_{1}}}}-\textbf{c}_{2}\frac{\varphi _{1}\overline{{\textbf{P}}_{\textbf{m}_{1}}}}{\overline{\textbf{S}}}=0,\\ \mathbf {c_{2}}\frac{(1-\textbf{m}_{1}-\textbf{m}_{2})\Lambda }{\overline{\textbf{S}}}+\textbf{c}_{3}\frac{\chi _{\textbf{q}}\overline{\textbf{S}}}{\overline{\textbf{P}}}=0,\\ \textbf{c}_{3}\frac{\delta _{3}\overline{\textbf{P}}}{\overline{{\textbf{P}}_{\textbf{T}}}}-\textbf{c}_{4}\frac{{\Im _{1}}\overline{{\textbf{P}}_{\textbf{T}}}}{\overline{{\textbf{S}}}}=0,\\ \textbf{c}_{4}\frac{\varphi _{2}\overline{{\textbf{H}}_{\textbf{P}}}}{\overline{{\textbf{S}}}}-\frac{\textbf{m}_{2}\Lambda }{\overline{{\textbf{H}}_{\textbf{m}_{1}}}}=0,\\ \end{array}\right. } \end{aligned}$$

where

$$\begin{aligned} \textbf{c}_{1}=\frac{\varphi _{1}\chi _{\textbf{q}}{\Im _{1}} \textbf{m}_{2}\overline{{\textbf{P}}_{\textbf{m}_{1}}}\overline{\textbf{S}}\overline{{\textbf{P}}}_{\textbf{T}}^{2}}{\delta _{3}\varphi _{2}(1-\textbf{m}_{1}-\textbf{m}_{2})\overline{\textbf{S}}\overline{{\textbf{H}}_{\textbf{m}_{1}}}\overline{\textbf{P}}},~~~\textbf{c}_{2}=\frac{-\chi _{\textbf{q}}{\Im _{1}}\Lambda \textbf{m}_{2}\overline{\textbf{S}}^{2}\overline{{\textbf{P}}_{\textbf{T}}^{2}}}{\Lambda (1-\textbf{m}_{1}-\textbf{m}_{2})\delta _{3}\varphi _{2}\overline{\textbf{P}}^{2}\overline{{\textbf{H}}_{\textbf{P}}}},~~~\mathbf {c_{3}}=\frac{{\Im _{1}}\Lambda \textbf{m}_{2}\overline{{\textbf{P}}_{\textbf{T}}^{2}}}{\delta _{3} \varphi _{2}\overline{\textbf{P}}\overline{{\textbf{H}}_{\textbf{P}}}},~~\textbf{c}_{4}=\frac{\textbf{m}_{2}\Lambda \overline{\textbf{S}}}{\varphi _{2}\overline{{\textbf{H}}_{\textbf{m}_{1}}}}. \end{aligned}$$

Implementing the Itô’s technique to $\Phi _{3}$ and considering (36)-(38), we have

39 $$\begin{aligned} \mathfrak {L}\Phi _{3}(\Lambda )\le \bigg (\frac{\textbf{m}_{2}\Lambda }{\overline{{\textbf{H}}_{\textbf{m}_{1}}}}-\mathbf {c_{1}}\frac{\textbf{m}_{1}\Lambda \overline{{\textbf{P}}_{\textbf{m}_{1}}}}{\overline{{\textbf{P}}_{\textbf{m}_{1}}}}-\textbf{c}_{2}\frac{\varphi _{2}\overline{{\textbf{S}}}}{\overline{{\textbf{S}}}}\bigg )\ln \widetilde{{\textbf{H}}_{\textbf{m}_{1}}}+c_{1}\delta _{1}{\textbf{H}}+\delta _{1}{\Im _{2}}{\textbf{Y}}. \end{aligned}$$

Furthermore, we indicate

$$\begin{aligned} \widetilde{{\textbf{H}}}=\frac{{\textbf{H}}}{\overline{\textbf{H}}},~~~\widetilde{{\textbf{H}}_{\textbf{X}}}=\frac{{{\textbf{H}}_{\textbf{X}}}}{\overline{{\textbf{H}}_{\textbf{X}}}},~~~\widetilde{{\textbf{Y}}}=\frac{{{\textbf{Y}}}}{\overline{{\textbf{Y}}}},~~~~\widetilde{{\textbf{T}}_{\textbf{H}\textbf{X}}}=\frac{{{\textbf{T}}_{\textbf{H}\textbf{X}}}}{\overline{{\textbf{T}}_{\textbf{H}\textbf{X}}}}. \end{aligned}$$

Implementing Itô’s technique to $-\ln {{\textbf{H}}},$ one obtains

40 $$\begin{aligned} \mathfrak {L}(-\ln {\textbf{H}}){} & {} \le -\frac{\chi _{\textbf{H}}\textbf{S}}{\textbf{H}}-\frac{\varphi _{3}\chi _{\textbf{H}}{\textbf{P}}_{\textbf{m}_{1}}}{\textbf{H}}+(\eta +\tau +\varphi _{4}+\varphi _{5}\chi _{\textbf{q}})+\frac{\wp _{4}^{2}}{2}\nonumber \\ {}{} & {} \le -\frac{\chi _{\textbf{H}}\textbf{S}}{\textbf{H}}-\frac{\varphi _{3}\chi _{\textbf{H}}{\textbf{P}}_{\textbf{m}_{1}}}{\textbf{H}}+(\eta +\tau +\varphi _{4}+\varphi _{5}\chi _{\textbf{q}})+\frac{\wp _{4}^{2}}{2}-\frac{\chi _{\textbf{H}}\widetilde{\textbf{S}}}{\widetilde{\textbf{H}}}\Big (\frac{\widetilde{\textbf{S}}}{\widetilde{\textbf{H}}}-1\Big )-\frac{\varphi _{3}\chi _{\textbf{H}}{\textbf{P}}_{\textbf{m}_{1}}}{\textbf{H}}\Big (\frac{\widetilde{{\textbf{P}}_{\textbf{m}_{1}}}}{\widetilde{\textbf{H}}}-1\Big )\nonumber \\ {}{} & {} \le -(\mathbb {R}_{0}^{\mathbb {S}}-1)\Big (\eta +\tau +\varphi _{4}+\varphi _{5}\chi _{\textbf{q}}+\frac{\wp _{4}^{2}}{2}\Big )+\frac{\chi _{\textbf{H}}\widetilde{\textbf{S}}}{\widetilde{\textbf{H}}}\ln \widetilde{\textbf{S}}-\frac{\varphi _{3}\chi _{\textbf{H}}{\textbf{P}}_{\textbf{m}_{1}}}{\textbf{H}}\ln \widetilde{{\textbf{P}}_{\textbf{m}_{1}}}, \end{aligned}$$

where

$$\begin{aligned} \mathbb {R}_{0}^{\mathbb {S}}=\frac{\chi _{\textbf{H}}\widetilde{\textbf{S}}+\varphi _{3}\chi _{\textbf{H}}\widetilde{{\textbf{P}}_{\textbf{m}_{1}}}}{\eta +\tau +\varphi _{4}+\varphi _{5}\chi _{\textbf{q}}+\frac{\wp _{4}^{2}}{2}}. \end{aligned}$$

Analogously, implementing Itô’s technique to $-\ln {\textbf{H}}_{\textbf{X}},~~-\ln {\textbf{Y}}$ and $-\ln {\textbf{T}}_{\textbf{H}\textbf{X}},$ we find

41 $$\begin{aligned}{} & {} \mathfrak {L}(-\ln {\textbf{H}}_{\textbf{X}})\le \tau \frac{\widetilde{\textbf{H}}}{\overline{{\textbf{H}}_{\textbf{X}}}}\ln \widetilde{{\textbf{H}}_{\textbf{X}}}+(\eta +\eta _{1}+\varphi _{7}+\varphi _{6}\chi _{\textbf{q}})\frac{1}{\overline{{\textbf{H}}_{\textbf{X}}}}\widetilde{{\textbf{H}}_{\textbf{X}}},\nonumber \\{} & {} \mathfrak {L}(-\ln {\textbf{Y}})\le \zeta \chi _{\textbf{H}}\frac{\widetilde{\textbf{S}}}{\overline{{\textbf{Y}}}}\ln {\widetilde{\textbf{S}}}+\varphi _{5}\chi _{\textbf{q}}\frac{\widetilde{{\textbf{H}}}}{\overline{{\textbf{Y}}}}\ln {\widetilde{{\textbf{H}}}}+\varphi _{6}\chi _{\textbf{q}}\frac{\widetilde{{\textbf{H}}_{\textbf{X}}}}{\overline{{\textbf{Y}}}}\ln {\widetilde{{\textbf{H}}_{\textbf{X}}}}+\sigma \chi _{\textbf{q}}\frac{\widetilde{{\textbf{T}}_{\textbf{H}\textbf{X}}}}{\overline{{\textbf{Y}}}}\ln {\widetilde{{\textbf{T}}_{\textbf{H}\textbf{X}}}}\nonumber \\ {}{} & {} \qquad \qquad \qquad +(\eta +\eta _{3}+{\Im _{2}})\frac{\widetilde{{\textbf{Y}}}}{\overline{{\textbf{Y}}}}\ln {\widetilde{{\textbf{Y}}}},\nonumber \\{} & {} \mathfrak {L}(-\ln {{\textbf{T}}_{\textbf{H}\textbf{X}}})\le \varphi _{4}\frac{\widetilde{\textbf{H}}}{\overline{{\textbf{T}}_{\textbf{H}\textbf{X}}}}\ln {\widetilde{\textbf{H}}}+\varphi _{7}\frac{\widetilde{{\textbf{H}}_{\textbf{X}}}}{\overline{{\textbf{T}}_{\textbf{H}\textbf{X}}}}\ln {\widetilde{{\textbf{H}}_{\textbf{X}}}}+{\Im _{2}}\frac{\widetilde{\textbf{Y}}}{\overline{{\textbf{T}}_{\textbf{H}\textbf{X}}}}\ln {\widetilde{\textbf{Y}}}+(\eta +\sigma \chi _{\textbf{q}})\frac{\widetilde{{\textbf{T}}_{\textbf{H}\textbf{X}}}}{\overline{{\textbf{T}}_{\textbf{H}\textbf{X}}}}\ln {\widetilde{{\textbf{T}}_{\textbf{H}\textbf{X}}}}.\nonumber \\ \end{aligned}$$

Introducing

42 $$\begin{aligned} \Phi _{4}(\Upsilon )=-\ln {\textbf{H}}_{\textbf{X}}-\textbf{d}_{1}\ln {\textbf{Y}}-\textbf{d}_{2}\ln {\textbf{T}}_{\textbf{H}\textbf{X}}-\textbf{d}_{3}\ln {\textbf{H}}, \end{aligned}$$

which leads to

43 $$\begin{aligned} {\left\{ \begin{array}{ll} \textbf{d}_{1}(\eta +\eta _{3}+{\Im _{2}})\frac{\widetilde{\textbf{Y}}}{\overline{\textbf{Y}}}+\textbf{d}_{2}\frac{{\Im _{2}}\widetilde{\textbf{Y}}}{\overline{{\textbf{T}}_{\textbf{H}\textbf{X}}}}-\delta _{2}\omega \frac{\widetilde{\textbf{Y}}}{\overline{{\textbf{H}}_{\textbf{X}}}}=0,\\ \textbf{d}_{2}\frac{\sigma \chi _{\textbf{q}}\widetilde{{\textbf{T}}_{\textbf{H}\textbf{X}}}}{\overline{\textbf{Y}}}+\textbf{d}_{3}(\eta +\sigma \chi _{\textbf{q}})\frac{\widetilde{{\textbf{T}}_{\textbf{H}\textbf{X}}}}{\overline{{\textbf{T}}_{\textbf{H}\textbf{X}}}}=0,\\ \textbf{d}_{3}\frac{\tau \widetilde{\textbf{H}}}{\overline{\textbf{H}}}+\varphi _{5}\chi _{\textbf{q}}\frac{\widetilde{\textbf{H}}}{\overline{\textbf{Y}}}+\frac{\varphi _{4}\widetilde{\textbf{H}}}{\overline{{\textbf{T}}_{\textbf{H}\textbf{X}}}}=0, \end{array}\right. } \end{aligned}$$

where

$$\begin{aligned}{} & {} \textbf{d}_{1}=\frac{\overline{\textbf{Y}}}{\widetilde{\textbf{Y}}(\eta +\eta _{3}+{\Im _{2}})}\Big \{-\frac{{\Im _{2}}\widetilde{\textbf{Y}}}{\widetilde{{\textbf{T}}_{\textbf{H}\textbf{X}}}}\frac{(\eta +\sigma \chi _{\textbf{q}})\overline{\textbf{Y}}\overline{\textbf{H}}(\varphi _{5}\chi _{\textbf{q}}\overline{\textbf{H}}\overline{{\textbf{T}}_{\textbf{H}\textbf{X}}}+\varphi _{4}\widetilde{\textbf{H}}\overline{\textbf{Y}})}{\sigma \chi _{\textbf{q}}\tau \overline{{\textbf{T}}_{\textbf{H}\textbf{X}}}\widetilde{\textbf{H}}\overline{\textbf{Y}}\overline{{\textbf{T}}_{\textbf{H}\textbf{X}}}}+\frac{\delta _{2}\omega \widetilde{\textbf{Y}}}{\overline{{\textbf{H}}_{\textbf{X}}}}\frac{\overline{\textbf{H}}(\varphi _{5}\chi _{\textbf{q}}\overline{\textbf{H}}\overline{{\textbf{T}}_{\textbf{H}\textbf{X}}}+\varphi _{4}\widetilde{\textbf{H}}\overline{\textbf{Y}})}{\tau \widetilde{\textbf{H}}\overline{\textbf{Y}}\overline{{\textbf{T}}_{\textbf{H}\textbf{X}}}}\Big \}, \\{} & {} \textbf{d}_{2}=\frac{-(\eta +\sigma \chi _{\textbf{q}})\overline{\textbf{Y}}\overline{\textbf{H}}(\varphi _{5}\chi _{\textbf{q}}\overline{\textbf{H}}\overline{{\textbf{T}}_{\textbf{H}\textbf{X}}}+\varphi _{4}\widetilde{\textbf{H}}\overline{\textbf{Y}})}{\sigma \chi _{\textbf{q}}\tau \overline{{\textbf{T}}_{\textbf{H}\textbf{X}}}\widetilde{\textbf{H}}\overline{\textbf{Y}}\overline{{\textbf{T}}_{\textbf{H}\textbf{X}}}},~~~~\textbf{d}_{3}=\frac{\overline{\textbf{H}}(\varphi _{5}\chi _{\textbf{q}}\overline{\textbf{H}}\overline{{\textbf{T}}_{\textbf{H}\textbf{X}}}+\varphi _{4}\widetilde{\textbf{H}}\overline{\textbf{Y}})}{\tau \widetilde{\textbf{H}}\overline{\textbf{Y}}\overline{{\textbf{T}}_{\textbf{H}\textbf{X}}}}. \end{aligned}$$

Now, considering (41)-(43), we have

44 $$\begin{aligned} \mathfrak {L}\Phi _{4}(\Lambda )=-(\mathbb {R}_{0}^{\mathbb {S}}-1)\Big (\eta +\tau +\varphi _{4}+\varphi _{5}\chi _{\textbf{q}}+\frac{\wp _{4}^{2}}{2}\Big )-\frac{\delta _{1}\widetilde{\textbf{H}}}{\widetilde{{{\textbf{P}}}_{\textbf{m}_{1}}}}\ln \widetilde{{\textbf{P}}_{\textbf{m}_{1}}}-\frac{\delta _{2}\widetilde{\textbf{P}}}{\widetilde{{\textbf{P}}_{\textbf{m}_{1}}}}\ln \widetilde{\textbf{P}}. \end{aligned}$$

Furthermore, we describe

45 $$\begin{aligned} \Phi _{5}(\Upsilon )=\Phi _{4}(\Upsilon )+\textbf{e}_{1}\Phi _{1}(\Upsilon )+\textbf{e}_{2}\Phi _{2}(\Upsilon )+\textbf{e}_{3}\Phi _{3}(\Upsilon ). \end{aligned}$$

Thus, we conclude that $\textbf{e}_{1}=\frac{\delta _{1}\overline{\textbf{S}}^{2}\overline{{\textbf{H}}_{\textbf{X}}}}{(\Lambda \textbf{m}_{1}\overline{\textbf{S}}+\textbf{m}_{2}\Lambda \overline{{\textbf{H}}_{\textbf{m}_{1}}})\overline{{\textbf{P}}_{\textbf{m}_{1}}}},~~~~\textbf{e}_{2}=\frac{\delta _{2}\overline{\textbf{S}}\overline{{\textbf{H}}_{\textbf{X}}}^{2}}{(\Lambda \textbf{m}_{2}\overline{\textbf{S}}+\textbf{c}_{1}\textbf{m}_{1}\Lambda \overline{{\textbf{H}}_{\textbf{m}_{1}}})\overline{{\textbf{P}}}}$ and $\textbf{e}_{3}=\frac{\textbf{d}_{2}\overline{\textbf{H}}\overline{{\textbf{P}}_{\textbf{T}}}^{2}}{(\varphi _{4}\overline{{\textbf{Y}}}+\varphi _{7}\overline{{\textbf{T}}_{\textbf{H}\textbf{X}}}-\sigma \chi _{\textbf{q}}\overline{{\textbf{H}}_{\textbf{m}_{1}}})}.$

In view of (33), (39) and (44), we have

46 $$\begin{aligned} \mathfrak {L}\Phi _{5}(\Lambda )\le -(\mathbb {R}_{0}^{\mathbb {S}}-1)\Big (\eta +\tau +\varphi _{4}+\varphi _{5}\chi _{\textbf{q}}+\frac{\wp _{4}^{2}}{2}\Big )+\delta _{1}(\textbf{e}_{1}+\textbf{c}_{1}\textbf{e}_{2}){{\textbf{H}}_{\textbf{m}_{1}}}+\delta _{2}(\textbf{b}_{2}\textbf{e}_{1}+\textbf{e}_{2}){\textbf{H}}. \end{aligned}$$

Introducing

47 $$\begin{aligned} \Phi _{6}(\Upsilon )=\Phi _{5}(\Upsilon )-\frac{\delta _{2}(\textbf{b}_{2}\textbf{e}_{1}+\textbf{e}_{2})}{{\Im _{2}}\tau }{{\textbf{H}}_{\textbf{X}}}. \end{aligned}$$

Again, implementing the Itô’s technique to $\Phi _{6},$ we have

48 $$\begin{aligned} \mathfrak {L}\Phi _{6}(\Upsilon )\le -(\mathbb {R}_{0}^{\mathbb {S}}-1)\Big (\eta +\tau +\varphi _{4}+\varphi _{5}\chi _{\textbf{q}}+\frac{\wp _{4}^{2}}{2}\Big )+\big [\delta _{1}(\textbf{e}_{1}+\textbf{c}_{1}\textbf{e}_{2})+\frac{\delta _{2}(\textbf{b}_{2}\textbf{e}_{1}+\textbf{e}_{2})(\varphi _{1}+\varphi -{2}+\chi _{\textbf{q}}+{\Im _{1}})}{{\Im _{2}}_{2}\tau }\big ]{\textbf{H}}_{\textbf{X}}.\nonumber \\ \end{aligned}$$

Introducing

49 $$\begin{aligned} \Phi _{7}(\Upsilon )=-\ln {\textbf{S}}-\ln {\textbf{P}}_{\textbf{m}_{1}}-\ln {\textbf{H}}_{\textbf{m}_{1}}-\ln {\textbf{H}}-\ln {\textbf{H}}_{\textbf{X}}-\ln {\textbf{P}}-\ln {\textbf{Y}}-\ln {{\textbf{P}}_{\textbf{T}}}. \end{aligned}$$

Implementing the Itô’s technique to $\Phi _{7},$ we have

50 $$\begin{aligned} \mathfrak {L}\Phi _{7}(\Upsilon ){} & {} \le -\frac{(1-\textbf{m}_{1}-\textbf{m}_{2})\Lambda }{\textbf{S}}-\varphi _{1}\frac{{\textbf{P}}_{\textbf{m}_{1}}}{\textbf{S}}-\varphi _{2}\frac{{\textbf{H}}_{\textbf{m}_{1}}}{\textbf{S}}-{\Im _{1}}\frac{{\textbf{P}}_{\textbf{T}}}{\textbf{S}}-\frac{\textbf{m}_{1}\Lambda }{{\textbf{P}}_{\textbf{m}_{1}}}-\frac{\textbf{m}_{2}\Lambda }{{\textbf{H}}_{\textbf{m}_{1}}}-\chi _{\textbf{H}}\frac{\textbf{S}}{\textbf{H}}-\varphi _{3}\chi _{\textbf{H}}\frac{{\textbf{P}}_{\textbf{m}_{1}}}{{\textbf{H}}}\nonumber \\ {}{} & {} \quad -\frac{\tau \textbf{H}}{{\textbf{H}}_{\textbf{X}}}-\chi _{\textbf{q}}\frac{\textbf{S}}{\textbf{P}}-\omega _{1}\chi _{\textbf{q}}\frac{{\textbf{H}}_{\textbf{m}_{1}}}{\textbf{P}}-\zeta \chi _{\textbf{H}}\frac{\textbf{P}}{\textbf{Y}}-\frac{\varphi _{5}\chi _{\textbf{q}}{\textbf{H}}}{\textbf{Y}}-\frac{\varphi _{6}\chi _{\textbf{q}}{\textbf{H}}_{\textbf{X}}-\sigma \chi _{\textbf{q}}{\textbf{T}}_{\textbf{H}\textbf{X}}}{\textbf{Y}}\nonumber \\ {}{} & {} \quad -\delta _{3}\frac{\textbf{P}}{{\textbf{P}}_{\textbf{T}}}+\chi _{\textbf{H}}(1+\varphi _{3}+\zeta )+\chi _{\textbf{q}}(1+\tilde{\omega _{1}}+\varphi _{5}+\varphi _{6}+\sigma )+9\eta +\varphi _{1}+\varphi _{2}+\tau +\varphi _{4}+\varphi _{7}+\eta _{1}\nonumber \\ {}{} & {} \quad +\varphi _{7}+\eta _{2}+\delta _{3}+\eta _{3}+{\Im _{2}}+{\Im _{1}}+\frac{1}{2}(\wp _{1}^{2}+\wp _{2}^{2}+\wp _{3}^{2}+\wp _{4}^{2}+\wp _{5}^{2}+\wp _{6}^{2}+\wp _{7}^{2}+\wp _{8}^{2}+\wp _{9}^{2}). \end{aligned}$$

Again, we describe

51 $$\begin{aligned} \Phi _{8}(\Upsilon )=\frac{1}{\bar{q}+1}\big ({\textbf{S}}+{\textbf{P}}_{\textbf{m}_{1}}+{\textbf{H}}_{\textbf{m}_{1}}+{\textbf{H}}+{\textbf{H}}_{\textbf{X}}+{\textbf{P}}+{\textbf{Y}}+{{\textbf{P}}_{\textbf{T}}}\big )^{\bar{q}+1}, \end{aligned}$$

where $$\bar{q}\in (0,1)$$ fulfilling

$$\begin{aligned} (\omega +\chi _{\textbf{q}})\wedge (\omega +\chi _{\textbf{H}})-\frac{\bar{q}}{2}(\wp _{1}^{2}\vee \wp _{2}^{2}\vee \wp _{3}^{2}\vee \wp _{4}^{2}\vee \wp _{5}^{2}\vee \wp _{6}^{2}\vee \wp _{7}^{2}\vee \wp _{8}^{2}\vee \wp _{9}^{2})>0. \end{aligned}$$

Employing the Itô’s technique to $\Phi _{8},$ we have

52 $$\begin{aligned}{} \mathfrak {L}\Phi _{8}(\Upsilon ) & =\big ({\textbf{S}}+{\textbf{P}}_{\textbf{m}_{1}}+{\textbf{H}}_{\textbf{m}_{1}}+{\textbf{H}}+{\textbf{H}}_{\textbf{X}}+{\textbf{P}}+{\textbf{Y}}+{{\textbf{P}}_{\textbf{T}}}\big )^{\bar{q}} \\ & \quad \times \big [(1-\textbf{m}_{1}-\textbf{m}_{2})\Lambda -(\chi _{\textbf{H}}+\chi _{\textbf{q}})(\textbf{S}+{\textbf{P}}_{\textbf{m}_{1}}+\omega _{1}{\textbf{H}}_{\textbf{m}_{1}}+\varphi _{5}{\textbf{H}}+\varphi _{6}{\textbf{H}}_{\textbf{X}}+\zeta {\textbf{P}}+{\Im _{2}} \textbf{Y}+{\Im _{1}}{\textbf{P}}_{\textbf{T}})\big ] \\ & \quad +\frac{\bar{q}}{2}\big ({\textbf{S}}+{\textbf{P}}_{\textbf{m}_{1}}+{\textbf{H}}_{\textbf{m}_{1}}+{\textbf{H}}+{\textbf{H}}_{\textbf{X}}+{\textbf{P}}+{\textbf{Y}}+{{\textbf{P}}_{\textbf{T}}}\big )^{\bar{q}-1} \\ & \quad \times \big (\wp _{1}^{2}{\textbf{S}}^{2}+\wp _{2}^{2}{{\textbf{P}}_{\textbf{m}_{1}}}^{2}+\wp _{3}^{2}{\textbf{H}}_{\textbf{m}_{1}}^{2}+\wp _{4}^{2}{\textbf{H}}^{2}+\wp _{5}^{2}{\textbf{H}}_{\textbf{X}}^{2}+\wp _{6}^{2}{\textbf{P}}^{2}+\wp _{7}^{2}{\textbf{Y}}^{2}+\wp _{8}^{2}{{\textbf{P}}_{\textbf{T}}}^{2}+\wp _{9}^{2}{{\textbf{T}}_{\textbf{H}\textbf{X}}}^{2}\big ) \\ & \quad \le \big ({\textbf{S}}+{\textbf{P}}_{\textbf{m}_{1}}+{\textbf{H}}_{\textbf{m}_{1}}+{\textbf{H}}+{\textbf{H}}_{\textbf{X}}+{\textbf{P}}+{\textbf{Y}}+{{\textbf{P}}_{\textbf{T}}}\big )^{\bar{q}} \\ & \quad \times \Big [(1-\textbf{m}_{1}-\textbf{m}_{2})-[(\omega +\chi _{\textbf{q}})\wedge (\omega +\chi _{\textbf{H}})]\big ({\textbf{S}}+{\textbf{P}}_{\textbf{m}_{1}}+{\textbf{H}}_{\textbf{m}_{1}}+{\textbf{H}}+{\textbf{H}}_{\textbf{X}}+{\textbf{P}}+{\textbf{Y}}+{{\textbf{P}}_{\textbf{T}}}\big )\Big ]\\ & \quad +\frac{\bar{q}}{2}\big ({\textbf{S}}+{\textbf{P}}_{\textbf{m}_{1}}+{\textbf{H}}_{\textbf{m}_{1}}+{\textbf{H}}+{\textbf{H}}_{\textbf{X}}+{\textbf{P}}+{\textbf{Y}}+{{\textbf{P}}_{\textbf{T}}}\big )^{\bar{q}+1}\nonumber \\ {}{} & {} \quad \times \big (\wp _{1}^{2}\vee \wp _{2}^{2}\vee \wp _{3}^{2}\vee \wp _{4}^{2}\vee \wp _{5}^{2}\vee \wp _{6}^{2}\vee \wp _{7}^{2}\vee \wp _{9}^{2}\big ) \\ & \quad \le \textbf{V}-\frac{\tilde{\bar{q}}}{2}\big ({\textbf{S}}+{\textbf{P}}_{\textbf{m}_{1}}+{\textbf{H}}_{\textbf{m}_{1}}+{\textbf{H}}+{\textbf{H}}_{\textbf{X}}+{\textbf{P}}+{\textbf{Y}}+{{\textbf{P}}_{\textbf{T}}}\big )^{\bar{q}+1}, \end{aligned}$$

where

53 $$\begin{aligned} \textbf{V}{} & {} =\sup \limits _{\Lambda \in \mathbb {R}_{+}^{9}}\Big \{(1-\textbf{m}_{1}-\textbf{m}_{2})\Lambda \big ({\textbf{S}}+{\textbf{P}}_{\textbf{m}_{1}}+{\textbf{H}}_{\textbf{m}_{1}}+{\textbf{H}}+{\textbf{H}}_{\textbf{X}}+{\textbf{P}}+{\textbf{Y}}+{{\textbf{P}}_{\textbf{T}}}\big )^{\bar{q}}\nonumber \\ {}{} & {} \quad -\frac{\tilde{\bar{q}}}{2}\big ({\textbf{S}}+{\textbf{P}}_{\textbf{m}_{1}}+{\textbf{H}}_{\textbf{m}_{1}}+{\textbf{H}}+{\textbf{H}}_{\textbf{X}}+{\textbf{P}}+{\textbf{Y}}+{{\textbf{P}}_{\textbf{T}}}\big )^{\bar{q}+1}\Big \} \end{aligned}$$

and

54 $$\begin{aligned} \tilde{\bar{q}}=(\omega +\chi _{\textbf{q}})\wedge (\omega +\chi _{\textbf{H}})-\frac{\bar{q}}{2}(\wp _{1}^{2}\vee \wp _{2}^{2}\vee \wp _{3}^{2}\vee \wp _{4}^{2}\vee \wp _{5}^{2}\vee \wp _{6}^{2}\vee \wp _{7}^{2}\vee \wp _{9}^{2}). \end{aligned}$$

Here, introducing a $\mathbb {C}^{2}$-function $\Phi _{9}$ on $\mathbb {R}_{+}^{9}\mapsto \mathbb {R}$

55 $$\begin{aligned} \Phi _{9}(\Lambda )=\mathbb {M}\Phi _{6}(\Lambda )+\Phi _{7}(\Lambda )+\Phi _{8}(\Lambda ), \end{aligned}$$

where $\mathbb {M}$ is a sufficiently significant non-negative constant that satisfies

56 $$\begin{aligned} -\mathbb {M}(\mathbb {R}_{0}^{\textbf{S}}-1)\Big (\eta +\tau +\varphi _{4}+\varphi _{5}\chi _{\textbf{q}}+\frac{\wp _{4}^{2}}{2}\Big )+\mathcal {W}\le -2 \end{aligned}$$

and

57 $$\begin{aligned} \mathcal {W}{} & {} =\sup \limits _{\Lambda \in \mathbb {R}_{+}^{9}}\Big \{\chi _{\textbf{H}}(1+\varphi _{3}+\zeta )+\chi _{\textbf{q}}(1+\tilde{\omega _{1}}+\varphi _{5}+\varphi _{6}+\sigma )+9\eta +\varphi _{1}+\varphi _{2}+\tau +\varphi _{4}+\varphi _{7}+\eta _{1}\nonumber \\ {}{} & {} \quad +\varphi _{7}+\eta _{2}+\delta _{3}+\eta _{3}+{\Im _{2}}+{\Im _{1}}+\frac{1}{2}(\wp _{1}^{2}+\wp _{2}^{2}+\wp _{3}^{2}+\wp _{4}^{2}+\wp _{5}^{2}+\wp _{6}^{2}+\wp _{7}^{2}+\wp _{8}^{2}+\wp _{9}^{2})+\textbf{V}+\delta _{1}{\textbf{H}}_{\textbf{X}}+\delta _{2}{\textbf{P}}\nonumber \\ {}{} & {} \quad -\frac{\tilde{\bar{q}}}{2}\big ({\textbf{S}}+{\textbf{P}}_{\textbf{m}_{1}}+{\textbf{H}}_{\textbf{m}_{1}}+{\textbf{H}}+{\textbf{H}}_{\textbf{X}}+{\textbf{P}}+{\textbf{Y}}+{{\textbf{P}}_{\textbf{T}}}\big )^{\bar{q}+1}\Big \}. \end{aligned}$$

Examine that the minimal point $\tilde{\Upsilon }\in \mathbb {R}_{+}^{9}$ of $\Phi _{9}(\Upsilon )$ appears to exist, therefore we conclude

58 $$\begin{aligned} \Phi (\Upsilon )=\Phi _{9}(\Upsilon )-\Phi _{9}(\tilde{\Upsilon }). \end{aligned}$$

Merging (48), (50) and (52), we have

59 $$\begin{aligned} \mathfrak {L}\Phi (\Upsilon ){} & {} \le -\mathbb {M}(\mathbb {R}_{0}^{\mathbb {S}}-1)\Big (\eta +\tau +\varphi _{4}+\varphi _{5}\chi _{\textbf{q}}+\frac{\wp _{4}^{2}}{2}\Big )+\mathcal {W}\nonumber \\ {}{} & {} \quad +\mathbb {M}\Big [\delta _{1}(\textbf{e}_{1}+\textbf{c}_{1}\textbf{e}_{2})+\frac{\delta _{2}(\textbf{b}_{2}\textbf{e}_{1}+\textbf{e}_{2})(\varphi _{1}+\varphi _{2}+\chi _{\textbf{q}}+{\Im _{1}})}{{\Im _{2}}_{2}\tau }\Big ]{\textbf{H}}_{\textbf{X}}-\frac{(1-\textbf{m}_{1}-\textbf{m}_{2})\Lambda }{\textbf{S}}-\varphi _{1}\frac{{\textbf{P}}_{\textbf{m}_{1}}}{\textbf{S}}\nonumber \\ {}{} & {} \quad -\varphi _{2}\frac{{\textbf{H}}_{\textbf{m}_{1}}}{\textbf{S}}-{\Im _{1}}\frac{{\textbf{P}}_{\textbf{T}}}{\textbf{S}}-\frac{\textbf{m}_{1}\Lambda }{{\textbf{P}}_{\textbf{m}_{1}}}-\frac{\textbf{m}_{2}\Lambda }{{\textbf{H}}_{\textbf{m}_{1}}}-\chi _{\textbf{H}}\frac{\textbf{S}}{\textbf{H}}-\varphi _{3}\chi _{\textbf{H}}\frac{{\textbf{P}}_{\textbf{m}_{1}}}{{\textbf{H}}}-\frac{\tau \textbf{H}}{{\textbf{H}}_{\textbf{X}}}-\chi _{\textbf{q}}\frac{\textbf{S}}{\textbf{P}}-\omega _{1}\chi _{\textbf{q}}\frac{{\textbf{H}}_{\textbf{m}_{1}}}{\textbf{P}}\nonumber \\ {}{} & {} \quad -\zeta \chi _{\textbf{H}}\frac{\textbf{P}}{\textbf{Y}}-\frac{\varphi _{5}\chi _{\textbf{q}}{\textbf{H}}}{\textbf{Y}}-\frac{\varphi _{6}\chi _{\textbf{q}}{\textbf{H}}_{\textbf{X}}-\sigma \chi _{\textbf{q}}{\textbf{T}}_{\textbf{H}\textbf{X}}}{\textbf{Y}}-\delta _{3}\frac{\textbf{P}}{{\textbf{P}}_{\textbf{T}}}+\chi _{\textbf{H}}(1+\varphi _{3}+\zeta )\nonumber \\ {}{} & {} \quad +\chi _{\textbf{q}}(1+\tilde{\omega _{1}}+\varphi _{5}+\varphi _{6}+\sigma )+9\eta +\varphi _{1}+\varphi _{2}+\tau +\varphi _{4}+\varphi _{7}+\eta _{1}\nonumber \\ {}{} & {} \quad +\varphi _{7}+\eta _{2}+\delta _{3}+\eta _{3}+{\Im _{2}}+{\Im _{1}}-\frac{\tilde{\bar{q}}}{4}\big ({\textbf{S}}+{\textbf{P}}_{\textbf{m}_{1}}+{\textbf{H}}_{\textbf{m}_{1}}+{\textbf{H}}+{\textbf{H}}_{\textbf{X}}+{\textbf{P}}+{\textbf{Y}}+{{\textbf{P}}_{\textbf{T}}}\big )^{\bar{q}+1}. \end{aligned}$$

Next, we construct the following for a bounded closed set:

60 $$\begin{aligned} \mathbb {V}_{\varphi }{} & {} =\Big \{\Upsilon \in \mathbb {R}_{+}^{9}:~\textbf{S}\in [\varphi ,1/\varphi ],~{\textbf{m}_{1}}_{\textbf{m}_{1}}\in [\varphi ^{2},1/\varphi ^{2}],~{\textbf{H}}_{\textbf{m}_{1}}\in [\varphi ^{3},1/\varphi ^{3}],~{{\textbf{H}}}\in [\varphi ^{2},1/\varphi ^{2}],~{\textbf{H}}_{\textbf{X}}\in [\varphi ,1/\varphi ],\nonumber \\ {}{} & {} \qquad \qquad \qquad ~{\textbf{P}}\in [\varphi ^{4},1/\varphi ^{4}],~{\textbf{Y}}\in [\varphi ^{2},1/\varphi ^{2}],~{\textbf{P}}_{\textbf{T}}\in [\varphi ^{3},1/\varphi ^{3}],~{\textbf{T}}_{\textbf{H}\textbf{X}}\in [\varphi ^{4},1/\varphi ^{4}]\Big \}, \end{aligned}$$

where $\varphi$ is a non-negative constant that is small enough to meet the subsequent variants

61 $$\begin{aligned}{} & {} -\frac{\Lambda (1-\textbf{m}_{1}-\textbf{m}_{2})\wedge \chi _{\textbf{q}}\wedge \omega \wedge \chi _{\textbf{H}}\wedge {\Im _{1}}}{\varphi }+\mathcal {F}\le -1,\nonumber \\{} & {} \mathbb {M}\Big [\delta _{1}(\textbf{e}_{1}+\textbf{c}_{1}\textbf{e}_{2})+\frac{\delta _{2}(\textbf{b}_{2}\textbf{e}_{1}+\textbf{e}_{2})(\varphi _{1}+\varphi _{2}+\chi _{\textbf{q}}+{\Im _{1}})}{{\Im _{2}}_{2}\tau }\Big ]\varphi \le 1,\nonumber \\ {}{} & {} -\tilde{\bar{q}}+8\mathcal {F}\varphi ^{\bar{q}}\le -8\varphi ^{\bar{q}},\nonumber \\ {}{} & {} -\tilde{\bar{q}}+8\mathcal {F}\varphi ^{\bar{q}+1}\le -8\varphi ^{\bar{q}+1},\nonumber \\ {}{} & {} -\tilde{\bar{q}}+8\mathcal {F}\varphi ^{2\bar{q}+2}\le -8\varphi ^{2\bar{q}+2},\nonumber \\{} & {} -\tilde{\bar{q}}+8\mathcal {F}\varphi ^{3\bar{q}+3}\le -8\varphi ^{3\bar{q}+3},\nonumber \\{} & {} -\tilde{\bar{q}}+8\mathcal {F}\varphi ^{4\bar{q}+4}\le -8\varphi ^{4\bar{q}+4}, \end{aligned}$$

having

62 $$\begin{aligned} \mathcal {F}=\sup \limits _{{\textbf{H}}_{\textbf{X}}\in \mathbb {R}_{+}^{9}}\Big \{\mathbb {M}\Big [\delta _{1}(\textbf{e}_{1}+\textbf{c}_{1}\textbf{e}_{2})+\frac{\delta _{2}(\textbf{b}_{2}\textbf{e}_{1}+\textbf{e}_{2})(\varphi _{1}+\varphi _{2}+\chi _{\textbf{q}}+{\Im _{1}})}{{\Im _{2}}_{2}\tau }\Big ]{\textbf{H}}_{\textbf{X}}-\frac{\tilde{\bar{q}}}{9}{{\textbf{H}}}_{\textbf{X}}^{\bar{q}+1}\Big \}. \end{aligned}$$

For the sake of simplicity, we can split $\mathbb {R}_{+}^{9}\setminus \mathbb {V}_{\varphi }$ into the subsequent eighteen regions:

63 $$\begin{aligned}{} & {} \mathbb {V}_{\varphi }^{1}=\big \{\Upsilon \in \mathbb {R}_{+}^{9}:~{\textbf{S}}\in (0,\varphi ]\big \},~~~\mathbb {V}_{\varphi }^{2}=\big \{\Upsilon \in \mathbb {R}_{+}^{9}:~{\textbf{P}}_{\textbf{m}_{1}}\in (0,\varphi ]\big \},\nonumber \\ {}{} & {} \mathbb {V}_{\varphi }^{3}=\big \{\Upsilon \in \mathbb {R}_{+}^{9}:~{\textbf{H}}_{\textbf{m}_{1}}\in (0,\varphi ^{3}],~{\textbf{S}}>\varphi ,~{\textbf{P}}_{\textbf{m}_{1}}>\varphi \big \},~~~\mathbb {V}_{\varphi }^{4}=\big \{\Upsilon \in \mathbb {R}_{+}^{9}:~{\textbf{H}}\in (0,\varphi ^{2}],~{\textbf{S}}>\varphi \big \},\nonumber \\ {}{} & {} \mathbb {V}_{\varphi }^{5}=\big \{\Upsilon \in \mathbb {R}_{+}^{9}:~{\textbf{H}}_{\textbf{X}}\in (0,\varphi ]\big \},~~~\mathbb {V}_{\varphi }^{6}=\big \{\Upsilon \in \mathbb {R}_{+}^{9}:~{\textbf{P}}\in (0,\varphi ^{4}],~{\textbf{H}}>\varphi ^{3}\big \},\nonumber \\ {}{} & {} \mathbb {V}_{\varphi }^{7}=\big \{\Upsilon \in \mathbb {R}_{+}^{9}:~{\textbf{Y}}\in (0,\varphi ^{2}],{\textbf{H}}_{\textbf{m}_{1}}>\varphi \big \},~~~\mathbb {V}_{\varphi }^{8}=\big \{\Upsilon \in \mathbb {R}_{+}^{9}:~{\textbf{P}}_{\textbf{T}}\in (0,\varphi ^{3}],~{{\textbf{T}}_{\textbf{H}\textbf{X}}}>\varphi ^{2}\big \},\nonumber \\ {}{} & {} \mathbb {V}_{\varphi }^{9}=\big \{\Upsilon \in \mathbb {R}_{+}^{9}:~{\textbf{T}}_{\textbf{H}\textbf{X}}\in (0,\varphi ^{4}]\big \},~~~ \mathbb {V}_{\varphi }^{10}=\big \{\Upsilon \in \mathbb {R}_{+}^{9}:~{\textbf{S}}\ge 1/\varphi \big \},\nonumber \\ {}{} & {} \mathbb {V}_{\varphi }^{11}=\big \{\Upsilon \in \mathbb {R}_{+}^{9}:~{\textbf{P}}_{\textbf{m}_{1}}\ge 1/\varphi ^{3}\big \},~~~ \mathbb {V}_{\varphi }^{12}=\big \{\Upsilon \in \mathbb {R}_{+}^{9}:~{\textbf{H}}_{\textbf{m}_{1}}\ge 1/\varphi \big \},\nonumber \\ {}{} & {} \mathbb {V}_{\varphi }^{13}=\big \{\Upsilon \in \mathbb {R}_{+}^{9}:~{\textbf{H}}_{\textbf{X}}\ge 1/\varphi ^{2}\big \},~~~ \mathbb {V}_{\varphi }^{14}=\big \{\Upsilon \in \mathbb {R}_{+}^{9}:~{\textbf{H}}\ge 1/\varphi \big \},\nonumber \\ {}{} & {} \mathbb {V}_{\varphi }^{15}=\big \{\Upsilon \in \mathbb {R}_{+}^{9}:~{\textbf{H}}_{\textbf{X}}\ge 1/\varphi ^{4}\big \},~~~ \mathbb {V}_{\varphi }^{16}=\big \{\Upsilon \in \mathbb {R}_{+}^{9}:~{\textbf{P}}\ge 1/\varphi ^{2}\big \},\nonumber \\ {}{} & {} \mathbb {V}_{\varphi }^{17}=\big \{\Upsilon \in \mathbb {R}_{+}^{9}:~{\textbf{P}}_{\textbf{T}}\ge 1/\varphi ^{3}\big \},~~~\mathbb {V}_{\varphi }^{18}=\big \{\Upsilon \in \mathbb {R}_{+}^{9}:~{\textbf{T}}_{\textbf{H}\textbf{X}}\ge 1/\varphi ^{4}\big \}. \end{aligned}$$

Evidently, $\mathbb {R}_{+}^{9}\setminus \mathbb {V}_{\varphi }=\bigcup \limits _{\jmath =1}^{18}\mathbb {V}_{\varphi }^{\jmath }.$ Consequently, it is easy to demonstrate that

64 $$\begin{aligned} \mathfrak {L}\Phi (\Upsilon )\le -1~~\forall ~\Lambda \in \mathbb {R}_{+}^{9}\setminus \mathbb {V}_{\varphi }. \end{aligned}$$

This verifies assumption $(\mathcal {H}_{1})$ of Lemma 3.1.

 The diffusion matrix for model (16) is presented as follows:

65 $$\begin{aligned} \textbf{V}= \begin{pmatrix} \wp _{1}^{2}{\textbf{S}}^{2}&{}0&{}0&{}0&{}0&{}0&{}0&{}0&{}0\\ 0&{}\wp _{2}^{2}{\textbf{P}}_{\textbf{m}_{1}}^{2}&{}0&{}0&{}0&{}0&{}0&{}0&{}0\\ 0&{}0&{}\wp _{3}^{2}{\textbf{H}}_{\textbf{m}_{1}}^{2}&{}0&{}0&{}0&{}0&{}0&{}0\\ 0&{}0&{}0&{}\wp _{4}^{2}{\textbf{H}}^{2}&{}0&{}0&{}0&{}0&{}0\\ 0&{}0&{}0&{}0&{}\wp _{5}^{2}{\textbf{H}}_{\textbf{X}}^{2}&{}0&{}0&{}0&{}0\\ 0&{}0&{}0&{}0&{}0&{}\wp _{6}^{2}{\textbf{P}}^{2}&{}0&{}0&{}0\\ 0&{}0&{}0&{}0&{}0&{}0&{}\wp _{7}^{2}{\textbf{Y}}^{2}&{}0&{}0\\ 0&{}0&{}0&{}0&{}0&{}0&{}0&{}\wp _{8}^{2}{{\textbf{P}}_{\textbf{T}}}^{2}&{}0\\ 0&{}0&{}0&{}0&{}0&{}0&{}0&{}0&{}\wp _{9}^{2}{{\textbf{T}}_{\textbf{H}\textbf{X}}}^{2}\\ \end{pmatrix}. \end{aligned}$$

It is evident that, matrix $\textbf{V}$ is positive definite $\forall ~\Upsilon \in \mathbb {V}.$ This verifies assumption $(\mathcal {H}_{1})$ of Lemma 3.1. Thus, the model (16) has a unique stationary distribution $\pi (.\,)$ and ergodic. This puts the proof to its conclusion. . $\square$

## Analysis of pneumonia

When the infection of HIV/AIDS is disregarded, the deterministic model (4) becomes the subsequent system:

66 $$\begin{aligned} {\left\{ \begin{array}{ll} \dot{\textbf{S}}=(1-\textbf{m}_{1})\Lambda +\varphi _{1}{\textbf{P}}_{\textbf{m}_{1}}+{\Im _{1}}{\textbf{P}}_{\textbf{T}}-(\chi _{\textbf{q}}+\eta )\textbf{S},\\ \dot{{\textbf{P}}_{\textbf{m}_{1}}}=\textbf{m}_{1}\Lambda -(\eta +\varphi _{1}){\textbf{P}}_{\textbf{m}_{1}},\\ \dot{{\textbf{P}}}=\chi _{\textbf{q}}\textbf{S}-(\eta +\eta _{2}+\delta _{3}){\textbf{P}},\\ \dot{{\textbf{P}}_{\textbf{T}}}=\delta _{3} {\textbf{P}}-(\eta +{\Im _{1}}){\textbf{P}}_{\textbf{T}}. \end{array}\right. } \end{aligned}$$

The basic reproduction number $\mathbb {R}_{\textbf{P}}^{0}$ of the model (66) is presented as

67 $$\begin{aligned} \mathbb {R}_{\textbf{P}}^{0}=\frac{\delta _{2}(\varphi _{1}+\eta (1-\textbf{m}_{1}))}{(\varphi _{1}+\eta )(\eta +\delta _{3}+\eta _{2})}. \end{aligned}$$

### Stochastic pneumonia model without HIV/AIDS infection

By utilizing the identical technique from probabilistic framework (16) to incorporate random perturbation, we obtain the subsequent stochastic model:

68 $$\begin{aligned} {\left\{ \begin{array}{ll} d{\textbf{S}}=\big [(1-\textbf{m}_{1})\Lambda +\varphi _{1}{\textbf{P}}_{\textbf{m}_{1}}+{\Im _{1}}{\textbf{P}}_{\textbf{T}}-(\chi _{\textbf{q}}+\eta )\textbf{S}\big ]d\textbf{t}+\wp _{4\jmath -3}{\textbf{S}}d{\mathbb {W}}_{4\jmath -3}(\textbf{t}),\\ d{{\textbf{P}}_{\textbf{m}_{1}}}=\big [\textbf{m}_{1}\Lambda -(\eta +\varphi _{1}){\textbf{P}}_{\textbf{m}_{1}}\big ]d\textbf{t}+\wp _{4\jmath -2}{{\textbf{P}}_{\textbf{m}_{1}}}d{\mathbb {W}}_{4\jmath -2}(\textbf{t}),\\ d{{\textbf{P}}}=\big [\chi _{\textbf{q}}\textbf{S}-(\eta +\eta _{2}+\delta _{3}){\textbf{P}}\big ]d\textbf{t}+\wp _{4\jmath -1}{{\textbf{P}}}d{\mathbb {W}}_{4\jmath -1}(\textbf{t}),\\ d{{\textbf{P}}_{\textbf{T}}}=\big [\delta _{3} {\textbf{P}}-(\eta +{\Im _{1}}){\textbf{P}}_{\textbf{T}}\big ]d\textbf{t}+\wp _{4\jmath }{{\textbf{P}}_{\textbf{T}}}d{\mathbb {W}}_{4\jmath }(\textbf{t}). \end{array}\right. } \end{aligned}$$

Then, we state

$$\begin{aligned} \mathbb {R}_{\jmath }^{\kappa }=\frac{\Lambda (1-\textbf{m}_{1}-\textbf{m}_{2})\varphi _{1}\varrho _{4}}{\varrho _{3}\varrho _{2}(\varrho _{4}\varrho _{1}-\chi _{\textbf{q}}{\Im _{1}})}. \end{aligned}$$

The values of the parameters have similar significance within the system (68). Indicate

69 $$\begin{aligned} \varrho _{1}=(\eta +\chi _{\textbf{q}})+\frac{\wp _{4\jmath -3}^{2}}{2},~~\varrho _{2}=(\eta +\varphi _{1})+\frac{\wp _{4\jmath -2}^{2}}{2},~~~\varrho _{3}=(\eta +\eta _{2}+\delta _{3})+\frac{\wp _{4\jmath -1}^{2}}{2},~~~\varrho _{4}=(\eta +{\Im _{1}})+\frac{\wp _{4\jmath }^{2}}{2}. \end{aligned}$$

The two theorems that proceed are derived from Sections 2 and 3 using a similar methodology.

#### Theorem 4.1

*Suppose there are initial values* $\big ({{\textbf{S}}}(0),{\textbf{P}}_{\textbf{m}_{1}}(0),{\textbf{P}}(0),{\textbf{P}}_{\textbf{T}}(0)\big )\in \mathbb {R}_{+}^{4}$ *have unique solution* $\big ({{\textbf{S}}}(\textbf{t}),{\textbf{P}}_{\textbf{m}_{1}}(\textbf{t}),{\textbf{P}}(\textbf{t}),{\textbf{P}}_{\textbf{T}}(\textbf{t})\big )\in \mathbb {R}_{+}^{4}$ *of the model* (68) with $\textbf{t}>0$ *and the solution will exist in* $\mathbb {R}_{+}^{4}$ *having probability* 1 (a.s).

#### Theorem 4.2

*Suppose that* $\mathbb {R}_{\jmath }^{\kappa }>1,$ *then model* (68) *possesses the ergodic functionality and yields a unique stationary distribution* $\pi (.\,).$

### Probability density function (P.D.F)

In what follows, we present a mathematical principle pertaining to the P.D.F associated with the subsequent probabilistic framework as:

70 $$\begin{aligned} d\Upsilon (\tau )={\hat{\textbf{c}}}(\Lambda ,\tau )d\tau +{\hat{\textbf{d}}}(\Upsilon ,\tau )d\textbf{W}(\tau ), \end{aligned}$$

where $\Upsilon$ indicates the parameter whilst ${\hat{\textbf{c}}}(\Upsilon ,\tau ),~{\hat{\textbf{d}}}(\Upsilon ,\tau )$ are some functions and $\textbf{W}(\tau )$ is the Wiener technique.

#### Lemma 4.1

(^54^) *Suppose there is a* *mapping* $\tilde{p}(\Upsilon )$ *states the P.D.F associated to the formula* (70):

$$\begin{aligned} \partial _{\tau }\tilde{p}(\Upsilon ,\tau \vert {\Upsilon _{0}},{\tau }_{0})=-\partial _{\Upsilon }\big [{\hat{\textbf{c}}}(\Upsilon ,\tau )\tilde{p}(\Upsilon ,\tau \vert {\Upsilon _{0}},{\tau }_{0})\big ]+\frac{1}{2}\partial _{\Upsilon }^{2}\Big ({\hat{\textbf{d}}}(\Upsilon ,\tau )^{2}\tilde{p}(\Upsilon ,\tau \vert {\Upsilon _{0}},{\tau }_{0})\Big ). \end{aligned}$$

Following that, we provide the prerequisites required to find the positive definite (P-D) 4D real symmetric matrix.

#### Lemma 4.2

*Assume that there is a 4D real* *algebraic equation* $\digamma _{0}^{2}+\textbf{W}\mho +\mho \textbf{W}^{\textbf{T}}=0$ *having* $\digamma _{0}=diag(1,0,0,0),$ *while* $\mho$ *indicates the real symmetric matrix*. 

(i) *If*

$$\begin{aligned} \textbf{W}= \begin{pmatrix} -\varrho _{1}&{}\varrho _{2}&{}-\varrho _{3}&{}-\varrho _{4}\\ 1&{}0&{}0&{}0\\ 0&{}1&{}0&{}0\\ 0&{}0&{}1&{}0 \end{pmatrix}, \end{aligned}$$

*containing with* $\varrho _{1}>0,\varrho _{3}>0,\varrho _{4}>0$ *and* $\varrho _{1}\varrho _{2}\varrho _{3}-\varrho _{3}^{2}-\varrho _{1}^{2}\varrho _{4}>0,$ *then*

71 $$\begin{aligned} \mho = \begin{pmatrix} \frac{\varrho _{2}\varrho _{3}-\varrho _{1}\varrho _{4}}{2(\varrho _{1}\varrho _{2}\varrho _{3}-\varrho _{3}^{2}-\varrho _{1}^{2}\varrho _{4})}&{}0&{}-\frac{\varrho _{3}}{2(\varrho _{1}\varrho _{2}\varrho _{3}-\varrho _{3}^{2}-\varrho _{1}^{2}\varrho _{4})}&{}0\\ 0&{}\frac{\varrho _{3}}{2(\varrho _{1}\varrho _{2}\varrho _{3}-\varrho _{3}^{2}-\varrho _{1}^{2}\varrho _{4})}&{}0&{}-\frac{\varrho _{1}}{2(\varrho _{1}\varrho _{2}\varrho _{3}-\varrho _{3}^{2}-\varrho _{1}^{2}\varrho _{4})}\\ -\frac{\varrho _{3}}{2(\varrho _{1}\varrho _{2}\varrho _{3}-\varrho _{3}^{2}-\varrho _{1}^{2}\varrho _{4})}&{}0&{}\frac{\varrho _{1}}{2(\varrho _{1}\varrho _{2}\varrho _{3}-\varrho _{3}^{2}-\varrho _{1}^{2}\varrho _{4})}&{}0\\ 0&{}-\frac{\varrho _{1}}{2(\varrho _{1}\varrho _{2}\varrho _{3}-\varrho _{3}^{2}-\varrho _{1}^{2}\varrho _{4})}&{}0&{}-\frac{\varrho _{1}\varrho _{2}-\varrho _{3}}{2(\varrho _{1}\varrho _{2}\varrho _{3}-\varrho _{3}^{2}-\varrho _{1}^{2}\varrho _{4})} \end{pmatrix} \end{aligned}$$

is a P-D. 

(ii) *If*

$$\begin{aligned} \textbf{W}=\begin{pmatrix} -\varrho _{1}&{}\varrho _{2}&{}-\varrho _{3}&{}\varrho _{4}\\ 1&{}0&{}0&{}0\\ 0&{}1&{}0&{}0\\ 0&{}0&{}1&{}\varrho _{5} \end{pmatrix}, \end{aligned}$$

containing $\varrho _{1}>0,\varrho _{3}>0$ and $\varrho _{1}\varrho _{2}-\varrho _{3}>0,$ *then*

72 $$\begin{aligned} \mho = \begin{pmatrix} \frac{\varrho _{2}}{2(\varrho _{1}\varrho _{2}-\varrho _{3})}&{}0&{}-\frac{1}{2(\varrho _{1}\varrho _{2}-\varrho _{3})}&{}0\\ 0&{}\frac{1}{2(\varrho _{1}\varrho _{2}-\varrho _{3})}&{}0&{}0\\ -\frac{1}{2(\varrho _{1}\varrho _{2}-\varrho _{3})}&{}0&{}\frac{\varrho _{1}}{2\varrho _{3}(\varrho _{1}\varrho _{2}-\varrho _{3})}&{}0\\ 0&{}0&{}0&{}0 \end{pmatrix} \end{aligned}$$

*is a semi P-D matrix*. X(iii) *If*

$$\begin{aligned} \textbf{W}=\begin{pmatrix} -\varrho _{1}&{}\varrho _{2}&{}\varrho _{3}&{}\varrho _{4}\\ 1&{}0&{}0&{}0\\ 0&{}0&{}\varrho _{5}&{}\varrho _{6}\\ 0&{}0&{}\varrho _{7}&{}\varrho _{8} \end{pmatrix}, \end{aligned}$$

*containing* $\varrho _{1}>0$ *and* $\varrho _{2}>0,$ *then*

73 $$\begin{aligned} \mho =\begin{pmatrix}(2\varrho _{1})^{-1}&{}0&{}0&{}0\\ 0&{}(2\varrho _{1}\varrho _{2})^{-1}&{}0&{}0\\ 0&{}0&{}0&{}0\\ 0&{}0&{}0&{}0 \end{pmatrix} \end{aligned}$$

*is a semi P-D matrix*.

#### Proof

Indicate the $\ell$-th significant main component of $\mho$ is $\mho ^{(\ell )},$ which is expressed as

(i) Observe that $\varrho _{1}(\varrho _{2}\varrho _{3}-\varrho _{1}\varrho _{4})>\varrho _{3}^{2}>0,$ then

$$\begin{aligned} \mho ^{(\Bbbk )}={\left\{ \begin{array}{ll} \frac{\varrho _{2}\varrho _{3}-\varrho _{1}\varrho _{4}}{2(\varrho _{1}\varrho _{2}\varrho _{3}-\varrho _{3}^{2}-\varrho _{1}^{2}\varrho _{4})}>0,~~\Bbbk =1\\ \frac{\varrho _{3}(\varrho _{2}\varrho _{3}-\varrho _{1}\varrho _{4})}{4(\varrho _{1}\varrho _{2}\varrho _{3}-\varrho _{3}^{2}-\varrho _{1}^{2}\varrho _{4})^{2}}>0,~~\Bbbk =2\\ \frac{\varrho _{3}}{8(\varrho _{1}\varrho _{2}\varrho _{3}-\varrho _{3}^{2}-\varrho _{1}^{2}\varrho _{4})^{2}}>0,~~\Bbbk =3\\ \frac{1}{16(\varrho _{1}\varrho _{2}\varrho _{3}-\varrho _{3}^{2}-\varrho _{1}^{2}\varrho _{4})^{2}}>0,~~\Bbbk =4. \end{array}\right. } \end{aligned}$$

Furthermore, assertions(ii) and (iii) can be obtained in the same way. $\square$

Here, the precise representation of the density function of system (68) at a quasi-equilibrium point will be derived. In relation to analytical importance, it is important to note that the P.D.F can represent the majority of the unpredictable features of a probabilistic process.

 Initially, we apply an analogous change to illustrate (68). For this, consider $\zeta _{4\jmath -3}=\ln {\textbf{S}},~\zeta _{4\jmath -2}=\ln {\textbf{P}}_{\textbf{m}_{1}},~\zeta _{4\jmath -1}=\ln {\textbf{P}}$ and $\zeta _{4\jmath }=\ln {\textbf{P}}_{\textbf{T}}.$ Thus, system (68)’s corresponding expression is provided by

74 $$\begin{aligned} {\left\{ \begin{array}{ll} d\zeta _{4\jmath -3}=\big [\pi (1-\textbf{m}_{1}) e^{-(4\jmath -3)}+\varphi _{1}e^{4\jmath -2}e^{-(4\jmath -3)}+{\Im _{1}} e^{4\jmath -1}e^{-(4\jmath -3)}+\varrho _{1}\big ]d\textbf{t}+\wp _{4\jmath -3}d{\mathbb {W}}_{4\jmath -3}(\textbf{t}),\\ d\zeta _{4\jmath -2}=\big [\textbf{m}_{1}\pi e^{-(4\jmath -2)}-\varrho _{2}\big ]d\textbf{t}+\wp _{4\jmath -2}d{\mathbb {W}}_{4\jmath -2}(\textbf{t}),\\ d\zeta _{4\jmath -1}=\big [\chi _{\textbf{q}} e^{4\jmath -3}e^{-(4\jmath -1)}-\varrho _{3}\big ]d\textbf{t}+\wp _{4\jmath -2}d{\mathbb {W}}_{4\jmath -2}(\textbf{t}),\\ d\zeta _{4\jmath }=\big [\delta _{3} e^{-4\jmath }-\varrho _{4}\big ]d\textbf{t}+\wp _{4\jmath }d{\mathbb {W}}_{4\jmath }(\textbf{t}), \end{array}\right. } \end{aligned}$$

When $\mathbb {R}_{0}^{\kappa }>1,$ we illustrate a quasi steady state $\mathcal {V}_{\jmath }^{*}=({\textbf{S}}_{\jmath }^{*},{{\textbf{P}}_{\textbf{P}}}_{\jmath }^{*},{{\textbf{P}}}_{\jmath }^{*},{{\textbf{P}}_{\textbf{T}}}_{\jmath }^{*}),$ where

75 $$\begin{aligned} {\textbf{S}}_{\jmath }^{*}=\frac{\varrho _{2}\varrho _{3}}{{\Im _{1}}\varphi _{1}},~~{{\textbf{P}}_{\textbf{P}}}_{\jmath }^{*}=\frac{\varrho _{3}{{\textbf{P}}}_{\jmath }^{*}}{\chi _{\textbf{q}}},~~{{\textbf{P}}}_{\jmath }^{*}=\frac{\varrho _{2}(\mathbb {R}_{0}^{\kappa }-1)(\varrho _{2}\varrho _{4}-\delta _{3}{\Im _{1}})}{(\chi _{\textbf{q}}+\eta )\varrho _{4}\big (\eta +\varphi +{\Im _{1}}+\frac{\wp _{4\jmath -2}^{2}}{2}\big )-\delta _{3}{\Im _{1}}},~~{\textbf{P}}_{{\textbf{T}} _{\jmath }}^{*}=\frac{\delta _{3}{{\textbf{P}}_{\textbf{T}}}_{\jmath }^{*}}{\varrho _{4}}. \end{aligned}$$

Assume that $\textbf{u}=\zeta _{\ell }-\zeta _{\ell }^{*},~(\ell =1,...,8).$ Thus, system (74) can be expressed as

76 $$\begin{aligned} {\left\{ \begin{array}{ll} d\textbf{u}_{4\jmath -3}=(-{\amalg _{11}}\textbf{u}_{4\jmath -3}+{\amalg _{12}}\textbf{u}_{4\jmath -2}-{\amalg _{13}}\textbf{u}_{4\jmath -1}-{\amalg _{14}}\textbf{u}_{4\jmath })d\tau +\wp _{4\jmath -3}d{\mathbb {W}}_{4\jmath -3}(\tau ),\\ d\textbf{u}_{4\jmath -2}=({\amalg _{22}}\textbf{u}_{4\jmath -3}+{\amalg _{22}}\textbf{u}_{4\jmath -2}-{\amalg _{22}}\textbf{u}_{4\jmath -1})d\tau +\wp _{4\jmath -2}d{\mathbb {W}}_{4\jmath -2}(\tau ),\\ d\textbf{u}_{4\jmath -1}=({\amalg _{33}}\textbf{u}_{4\jmath -2}-{\amalg _{33}}\textbf{u}_{4\jmath -2})d\tau +\wp _{4\jmath -1}d{\mathbb {W}}_{4\jmath -1}(\tau ),\\ d\textbf{u}_{4\jmath }=\big ({\amalg _{41}}\textbf{u}_{4\jmath -3}+{\amalg _{42}}\textbf{u}_{4\jmath -2}-({\amalg _{41}+\amalg _{42}})\textbf{u}_{4\jmath }\big )d\tau +\wp _{4\jmath }d{\mathbb {W}}_{4\jmath }(\tau ), \end{array}\right. } \end{aligned}$$

where $${\amalg _{11}}=\frac{\pi (1-\textbf{m}_{1})+\varphi _{1}{\textbf{P}}_{\textbf{m}_{1}}^{*}}{{\textbf{S}}_{\jmath }^{*}},~~{\amalg _{12}}=\frac{\varphi {{\textbf{P}}_{\textbf{P}}}_{\jmath }^{*}}{{\textbf{S}}_{\jmath }^{*}},~~{\amalg _{13}}={{\Im _{1}}{{{\textbf{P}}}}_{\jmath }^{*}},~~{\amalg _{14}}=\frac{\varphi _{1}{{\textbf{P}}_{\textbf{T}}}_{\jmath }^{*}}{{\textbf{S}}_{\jmath }^{*}},~~{\amalg _{22}}=\frac{\textbf{m}_{1}\pi {{\textbf{S}}}_{\jmath }^{*}{\textbf{P}}_{\jmath }^{*}}{{{\textbf{P}}_{\textbf{m}_{1}}}_{\jmath }^{*}},~~{\amalg _{33}}=\frac{\chi _{\textbf{q}}{{\textbf{P}}_{\textbf{P}}}_{\jmath }^{*}}{{{\textbf{P}}}_{\jmath }^{*}},~~{\amalg _{41}}=\frac{\delta _{3}{{\textbf{S}}}_{\jmath }^{*}}{{{\textbf{P}}_{\textbf{T}}}_{\jmath }^{*}},~~{\amalg _{42}}=\frac{\delta _{3}{{\textbf{P}}_{\textbf{m}_{1}}}_{\jmath }^{*}}{{{\textbf{P}}_{\textbf{T}}}_{\jmath }^{*}}.$$

Furthermore, $${\amalg _{11}}=\varrho _{1}{\amalg _{22}}+{\amalg _{13}}={\amalg _{33}}\varrho _{2}=\varrho _{3}$$ and $${\amalg _{44}}=\varrho _{4}.$$

Define $\Phi (\tau )=\big (\textbf{u}_{4\jmath -3}(\tau )....\textbf{u}_{4\jmath }(\tau )\big )$ and $\textbf{W}(\tau )=\big (\textbf{W}_{4\jmath -3}(\tau )....\textbf{W}_{4\jmath }(\tau )\big ),$ we have

$$\begin{aligned} d\Phi (\tau )=\textbf{W}\Phi (\tau )d\tau +\digamma d\textbf{W}(\tau ), \end{aligned}$$

where

77 $$\begin{aligned} \textbf{W}=\begin{pmatrix} -{\amalg _{11}}&{}{\amalg _{12}}&{}-{\amalg _{13}}&{}{\amalg _{14}}\\ {\amalg _{22}}&{}-{\amalg _{22}}&{}\amalg _{22}&{}0\\ 0&{}{\amalg _{33}}&{}-{\amalg _{33}}&{}0\\ {\amalg _{41}}&{}{\amalg _{42}}&{}0&{}-({\amalg _{14}+\amalg _{42}}) \end{pmatrix}~~and ~~\digamma =\begin{pmatrix} \wp _{4\jmath -3}&{}0&{}0&{}0\\ 0&{}\wp _{4\jmath -2}&{}0&{}0\\ 0&{}0&{}\wp _{4\jmath -1}&{}0\\ 0&{}0&{}0&{}\wp _{4\jmath } \end{pmatrix}. \end{aligned}$$

Next, we confirm that the real components of each of $\textbf{W}$’s eigenvalues are negative. The characteristic polynomial of $\textbf{W}$ that corresponds to it is ${\amalg _{\textbf{W}}}(\upsilon )={\tilde{a}}_{4}+{\tilde{a}}_{3}\psi _{1}+\tilde{a}_{2}\psi _{1}^{2}+{\tilde{a}}_{1}\psi _{1}^{3}+\psi _{1}^{4},$

where

78 $$\begin{aligned}{} & {} \tilde{a}_{1}={\amalg _{11}}+{\amalg _{22}}+{\amalg _{33}}+{\amalg _{41}}+{\amalg _{42}}>0,\nonumber \\ {}{} & {} \tilde{a}_{2}=({\amalg _{11}}-{\amalg _{12}}){\amalg _{22}}+({\amalg _{11}}-{\amalg _{14}}){\amalg _{41}}+({\amalg _{33}}+{\amalg _{42}}){\amalg _{11}}+({\amalg _{22}}+{\amalg _{33}})({\amalg _{41}}+{\amalg _{42}})>0,\nonumber \\ {}{} & {} \tilde{a}_{3}=({\amalg _{13}}-{\amalg _{12}}){\amalg _{22}}{\amalg _{33}}+({\amalg _{11}}-{\amalg _{12}}-{\amalg _{14}})({\amalg _{41}}+{\amalg _{42}}){\amalg _{22}}+({\amalg _{11}}-{\amalg _{14}}){\amalg _{33}}{\amalg _{41}}+{\amalg _{11}}{\amalg _{33}}{\amalg _{42}}>0,\nonumber \\ {}{} & {} \tilde{a}_{4}=\big (({\amalg _{13}}-{\amalg _{12}}-{\amalg _{14}})({\amalg _{41}}+{\amalg _{42}})+{\amalg _{14}}{\amalg _{41}}\big ){\amalg _{22}}{\amalg _{33}}. \end{aligned}$$

Following that, if $\mathbb {R}_{0}^{\kappa }>1,$ then

79 $$\begin{aligned}{} & {} {\amalg _{13}}-{\amalg _{12}}=\Big (1-\frac{\varphi _{1}}{\eta +\varphi _{1}+\frac{\wp _{4\jmath -2}^{2}}{2}}\Big )\textbf{m}_{1}\pi>0.\nonumber \\ {}{} & {} ({\amalg _{13}}-{\amalg _{12}}-{\amalg _{14}})({\amalg _{41}}+{\amalg _{42}})+{\amalg _{14}}{\amalg _{41}}=\Big (\frac{\pi }{{\textbf{S}}^{*}}-\varrho _{1}\Big )+\delta _{3}{\Im _{1}}=\varrho _{1}\varrho _{4}(\mathbb {R}_{0}^{\kappa }-1)>0.\nonumber \\ \tilde{a}_{1}\tilde{a}_{2}{} & {} \ge {\amalg _{33}}\big [({\amalg _{11}}-{\amalg _{12}}){\amalg _{22}}+({\amalg _{11}}-{\amalg _{14}}){\amalg _{41}}+{\amalg _{11}}{\amalg _{42}}\big ]+{\amalg _{11}}{\amalg _{22}}({\amalg _{41}}+{\amalg _{42}})\ge \tilde{a}_{3}+\tilde{a}_{1}(\tilde{a}_{2}\tilde{a}_{3}-\tilde{a}_{1}\tilde{a}_{4})\nonumber \\ {}{} & {} \ge \tilde{a}_{1}\big \{\big [({\amalg _{11}}({\amalg _{41}}+{\amalg _{42}}))+{\amalg _{22}}({\amalg _{11}}-{\amalg _{12}}+{\amalg _{41}+{\amalg _{42}}})-{\amalg _{14}}{\amalg _{41}}\big ]{\amalg _{22}}({\amalg _{11}}-{\amalg _{12}}-{\amalg _{14}})({\amalg _{41}}+{\amalg _{42}})\nonumber \\ {}{} & {} \quad +\big [{\amalg _{11}}({\amalg _{22}}+{\amalg _{33}}+{\amalg _{41}}+{\amalg _{42}})+{\amalg _{33}}({\amalg _{41}}+{\amalg _{42}})-{\amalg _{12}}{\amalg _{22}}-{\amalg _{14}}{\amalg _{41}}\big ]{\amalg _{33}}({\amalg _{11}}-{\amalg _{14}})({\amalg _{41}}+{\amalg _{42}})\nonumber \\ {}{} & {} \quad +{\amalg _{14}}{\amalg _{33}}{\amalg _{42}}\tilde{a}_{2}\big \} \nonumber \\ {}{} & {} \ge \big [{\amalg _{22}}({\amalg _{41}}+{\amalg _{42}})({\amalg _{11}}-{\amalg _{12}}-{\amalg _{14}})+({\amalg _{13}}-{\amalg _{12}}){\amalg _{22}}{\amalg _{33}}+({\amalg _{11}}-{\amalg _{14}}){\amalg _{33}}{\amalg _{41}}+{\amalg _{11}}{\amalg _{33}}{\amalg _{42}}\big ]\nonumber \\ {}{} & {} \quad \times {\amalg _{22}}({\amalg _{11}}-{\amalg _{12}}-{\amalg _{14}})({\amalg _{41}}+{\amalg _{42}})+\big [({\amalg _{11}}-{\amalg _{12}}-{\amalg _{14}})({\amalg _{41}}+{\amalg _{42}}){\amalg _{22}}+(\amalg _{13}-{\amalg _{12}}){\amalg _{22}}{\amalg _{33}}\nonumber \\ {}{} & {} \quad +({\amalg _{11}}-{\amalg _{14}}){\amalg _{33}}{\amalg _{41}}+{\amalg _{11}}{\amalg _{33}}{\amalg _{42}}\big ](\amalg _{13}-{\amalg _{12}}){\amalg _{22}}{\amalg _{33}}+\big [({\amalg _{11}}-{\amalg _{12}}-{\amalg _{14}})({\amalg _{41}}+{\amalg _{42}}){\amalg _{22}}\nonumber \\ {}{} & {} \quad +({\amalg _{13}}-{\amalg _{12}}){\amalg _{22}}{\amalg _{33}}+(\amalg _{11}-{\amalg _{41}}){\amalg _{33}}{\amalg _{41}}+{\amalg _{11}}{\amalg _{33}}{\amalg _{42}}\big ]{\amalg _{33}}({\amalg _{11}}-{\amalg _{14}})({\amalg _{41}}+{\amalg _{42}})+{\amalg _{14}}{\amalg _{33}}{\amalg _{42}}\tilde{a}_{1}\tilde{a}_{2}\nonumber \\ {}{} & {} >\tilde{a}_{3}^{2}. \end{aligned}$$

Thus, $\tilde{a}_{\jmath }>0,~(\jmath =1,...,4)~(\tilde{a}_{1}\tilde{a}_{2}-\tilde{a}_{3})>0$ and $\tilde{a}_{1}\tilde{a}_{2}\tilde{a}_{3}-\tilde{a}_{3}^{2}-\tilde{a}_{1}^{2}\tilde{a}_{4}>0.$ Subsequently it appears that A possesses every negative real-part eigenvalues that correspond to the Routh-Hurwitz stability condition^55^. With reference to Lemma 4.1, the Fokker-Planck equation below is satisfied by the relevant P.D.F $\mathcal {Q}(\Phi )$ to the Quasi-stationary condition of the system (74) can be expressed as

$$\begin{aligned}{} & {} \sum \limits _{\jmath =1}^{2}\Big (\frac{\wp _{4\jmath -3}^{2}}{2}\frac{\partial ^{2}\mathcal {Q}}{\partial \textbf{u}_{4\jmath -3}^{2}}+\frac{\wp _{4\jmath -2}^{2}}{2}\frac{\partial ^{2}\mathcal {Q}}{\partial \textbf{u}_{4\jmath -2}^{2}}+\frac{\wp _{4\jmath -1}^{2}}{2}\frac{\partial ^{2}\mathcal {Q}}{\partial \textbf{u}_{4\jmath -1}^{2}}+\frac{\wp _{4\jmath }^{2}}{2}\frac{\partial ^{2}\mathcal {Q}}{\partial \textbf{u}_{4\jmath }^{2}}\Big )\nonumber \\ {}{} & {} =\sum \limits _{\jmath =1}^{2}\Big \{\frac{\partial }{\partial \textbf{u}_{4\jmath -3}}\big ({\amalg _{14}}\textbf{u}_{4\jmath }-{\amalg _{13}}\textbf{u}_{4\jmath -1}-{\amalg _{11}}\textbf{u}_{4\jmath -3}\big )\mathcal {Q}+\frac{\partial }{\partial \textbf{u}_{4\jmath -2}}\big ({\amalg _{22}}\textbf{u}_{4\jmath -3}-{\amalg _{22}}\textbf{u}_{4\jmath -2}+{\amalg _{22}}\textbf{u}_{4\jmath -1}\big )\mathcal {Q}\nonumber \\ {}{} & {} \quad +\frac{\partial }{\partial \textbf{u}_{4\jmath -1}}\big ({\amalg _{33}}\textbf{u}_{4\jmath -2}-{\amalg _{33}}\textbf{u}_{4\jmath -2}\big )\mathcal {Q}+\frac{\partial }{\partial \textbf{u}_{4\jmath }}\big ({\amalg _{41}}\textbf{u}_{4\jmath -3}+{\amalg _{42}}\textbf{u}_{4\jmath -2}-({\amalg _{41}}+{\amalg _{42}})\textbf{u}_{4\jmath }\big )\mathcal {Q}\Big \}. \end{aligned}$$

Given that $\digamma$ is an invariant matrix, one can determine that $\mathcal {Q}(\Phi )$ is potentially identified as having a Gaussian distribution by incorporating the pertinent findings of Roozen^56^:

$$\begin{aligned} \mathcal {Q}(\Phi )=\tilde{c}\exp \Big (\frac{-1}{2}\Phi ^{\textbf{T}}\mathbb {Q}\Phi \Big ), \end{aligned}$$

where $\tilde{c}$ justifying $\int \limits _{\mathbb {R}_{+}^{4}}\tilde{c}\exp \Big (\frac{-1}{2}\Phi ^{\textbf{T}}\mathbb {Q}\Phi \Big )d\Phi =1$ and $\mathbb {Q}=({{\Im _{2}}_{1}}_{\jmath \kappa })_{4\times 4}$ is a real symmetric matrix fulfilling

80 $$\begin{aligned} \mathbb {Q}\digamma ^{2}\mathbb {Q}+\mathbb {Q}\textbf{W}+\textbf{W}^{\textbf{T}}\mathbb {Q}=0. \end{aligned}$$

If $\mathbb {Q}^{-1}$ holds, we indicate $\Pi =\mathbb {Q}^{-1},$ $\mathbb {Q}$ can be found to possess the equivalent degree of positive definiteness. Following this, (80) has the structure that follows.

81 $$\begin{aligned} \digamma ^{2}+\textbf{W}\Pi +\Pi \textbf{W}^{\textbf{T}}=0. \end{aligned}$$

The mathematical structure of (81) is obtained by employing a finitely autonomous coherence theory, which gives us $\digamma =\sum \limits _{\ell =1}^{4}\digamma _{\ell }$ and $\Pi =\sum \limits _{\ell =1}^{4},$ then we have

$$\begin{aligned} \digamma _{\ell }^{2}+\textbf{W}\Pi _{\ell }+\Pi _{\ell }\textbf{W}^{\textbf{T}}=0,~~\ell =1,...,4, \end{aligned}$$

where

$$\begin{aligned} \digamma _{1}=\begin{pmatrix} \wp _{4\jmath -3}^{2}&{}0&{}0&{}0\\ 0&{}0&{}0&{}0\\ 0&{}0&{}0&{}0\\ 0&{}0&{}0&{}0\\ \end{pmatrix},~~ \digamma _{2}=\begin{pmatrix} 0&{}0&{}0&{}0\\ 0&{}\wp _{4\jmath -2}^{2}&{}0&{}0\\ 0&{}0&{}0&{}0\\ 0&{}0&{}0&{}0\\ \end{pmatrix},~~\digamma _{3}=\begin{pmatrix} 0&{}0&{}0&{}0\\ 0&{}0&{}0&{}0\\ 0&{}0&{}\wp _{4\jmath -1}^{2}&{}0\\ 0&{}0&{}0&{}0\\ \end{pmatrix},~~\digamma _{4}=\begin{pmatrix} 0&{}0&{}0&{}0\\ 0&{}0&{}0&{}0\\ 0&{}0&{}0&{}0\\ 0&{}0&{}0&{}\wp _{4\jmath }^{2}\\ \end{pmatrix} \end{aligned}$$

and $\Pi _{\ell }$ are decided upon thereafter. 

Taking into account $\textbf{u}_{\ell }=\upsilon _{\ell }-\upsilon _{\ell }^{*}$ and the transformation between the frameworks (68) and (74) yields the following:

$$\begin{aligned} \mathcal {Q}(\tilde{\Phi })=\frac{1}{4\varpi _{2}^{2}}\vert \Pi \vert ^{-1/2}\exp \Big (\frac{-1}{2}\tilde{\Phi }\Pi ^{-1}\tilde{\Phi }^{\textbf{T}}\Big ), \end{aligned}$$

where $$\tilde{\Phi }=\Big (\ln \frac{\textbf{S}}{{\textbf{S}}^{*}},\ln \frac{{\textbf{P}}_{\textbf{m}_{1}}}{{\textbf{P}}_{\textbf{m}_{1}}^{*}},\ln \frac{{\textbf{P}}}{{\textbf{P}}^{*}},\ln \frac{{\textbf{P}}_{\textbf{T}}}{{{\textbf{P}}_{\textbf{T}}}^{*}}\Big ).$$

#### Theorem 4.3

Surmising that $\mathbb {R}_{0}^{\kappa }>1,$ for any $\big ({\textbf{S}}(0),{{\textbf{P}}_{\textbf{m}_{1}}}(0),{{\textbf{P}}}(0),{{\textbf{P}}_{\textbf{T}}}(0)\big )\in \mathbb {R}_{+}^{4},$ then the solution $\big ({\textbf{S}}(\textbf{t}),{{\textbf{P}}_{\textbf{m}_{1}}}(\textbf{t}),{{\textbf{P}}}(\textbf{t}),{{\textbf{P}}_{\textbf{T}}}(\textbf{t})\big )\in \mathbb {R}_{+}^{4}$ model (68) possess a log-normal P.D.F $\mathcal {Q}(\tilde{\Phi })$ about $\mathcal {V}_{\jmath }^{*}$ as follows $\tilde{\Phi }=\Big (\ln \frac{\textbf{S}}{{\textbf{S}}^{*}},\ln \frac{{\textbf{P}}_{\textbf{m}_{1}}}{{\textbf{P}}_{\textbf{m}_{1}}^{*}},\ln \frac{{\textbf{P}}}{{\textbf{P}}^{*}},\ln \frac{{\textbf{P}}_{\textbf{T}}}{{{\textbf{P}}_{\textbf{T}}}^{*}}\Big )$ having $\Pi =\Pi _{\ell },~(\ell =1,...,4)$ is a positive definite matrix and the components $\Pi _{1},\Pi _{2},\Pi _{3}$ and $\Pi _{4}$ are described as

82 $$\begin{aligned}{} & {} \Pi _{1}={\left\{ \begin{array}{ll} ({\amalg _{22}}{\amalg _{33}}{\amalg _{41}}\wp _{4\jmath -3})^{2}(\mathcal {V}_{1}\textbf{K}_{1})^{-1}\mho _{1}\big [(\mathcal {V}_{1}\textbf{K}_{1})^{-1}\big ]^{\textbf{T}},~~~~if~~~~\vartheta _{1}=0,\\ ({\amalg _{22}}{\amalg _{33}}\wp _{4\jmath -3})^{2}(\mathcal {V}_{2}\textbf{K}_{2}\textbf{K}_{1})^{-1}\mho _{2}\big [(\mathcal {V}_{2}\textbf{K}_{2}\textbf{K}_{1})^{-1}\big ]^{\textbf{T}},~~~~if~~~~\vartheta _{1}\ne 0,~\vartheta _{2}=0,\\ ({\amalg _{22}}{\amalg _{33}}\vartheta _{2}\wp _{4\jmath -3})^{2}(\mathcal {V}_{3}\textbf{K}_{2}\textbf{K}_{1})^{-1}\mho _{1}\big [(\mathcal {V}_{3}\textbf{K}_{2}\textbf{K}_{1})^{-1}\big ]^{\textbf{T}},~~~~if~~~~\vartheta _{1}\ne 0,~\vartheta _{2}\ne 0,\\ \end{array}\right. }\nonumber \\ {}{} & {} \Pi _{2}= {\left\{ \begin{array}{ll} ({\amalg _{12}}\wp _{4\jmath -2})^{2}(\mathcal {V}_{4}\textbf{K}_{3})^{-1}\mho _{3}\big [(\mathcal {V}_{4}\textbf{K}_{3})^{-1}\big ]^{\textbf{T}},~~~~if~~~~\vartheta _{3}=0,~\vartheta _{4}=0,\\ ({\amalg _{14}}{\amalg _{33}}\vartheta _{3}\wp _{4\jmath -2})^{2}(\mathcal {V}_{5}\textbf{K}_{3})^{-1}\mho _{1}\big [(\mathcal {V}_{5}\textbf{K}_{3})^{-1}\big ]^{\textbf{T}},~~~~if~~~~\vartheta _{3}\ne 0,~\vartheta _{4}=0,\\ ({\amalg _{13}}{\amalg _{42}}\vartheta _{4}\wp _{4\jmath -2})^{2}(\mathcal {V}_{6}\textbf{K}_{4}\textbf{K}_{3})^{-1}\mho _{1}\big [(\mathcal {V}_{6}\textbf{K}_{4}\textbf{K}_{3})^{-1}\big ]^{\textbf{T}},~~~~if~~~~\vartheta _{3}=0,~\vartheta _{4}\ne 0, ~\vartheta _{3}\ne 0,~\vartheta _{4}=0,\\ ({\amalg _{12}}\vartheta _{4}\wp _{4\jmath -2})^{2}(\mathcal {V}_{7}\textbf{K}_{5}\textbf{K}_{3})^{-1}\mho _{4}\big [(\mathcal {V}_{7}\textbf{K}_{5}\textbf{K}_{3})^{-1}\big ]^{\textbf{T}},~~~~if~~~~\vartheta _{3}\ne 0,~\vartheta _{4}\ne 0,\vartheta _{5}=0,\\ ({\amalg _{12}}\vartheta _{4}\vartheta _{5}\wp _{4\jmath -2})^{2}(\mathcal {V}_{8}\textbf{K}_{5}\textbf{K}_{3})^{-1}\mho _{1}\big [(\mathcal {V}_{8}\textbf{K}_{5}\textbf{K}_{3})^{-1}\big ]^{\textbf{T}},~~~~if~~~~\vartheta _{3}\ne 0,~\vartheta _{4}\ne 0,\vartheta _{5}\ne 0,\\ \end{array}\right. }\nonumber \\{} & {} \Pi _{3}={\left\{ \begin{array}{ll} (\amalg _{13}\wp _{4\jmath -1})^{2}(\mathcal {V}_{9}\textbf{K}_{6})^{-1}\mho _{5}\big [(\mathcal {V}_{9}\textbf{K}_{6})^{-1}\big ]^{\textbf{T}}~~~~if~~~\vartheta _{6}=0,\\ (\amalg _{13}{\amalg _{42}}\vartheta _{6}\wp _{4\jmath -1})^{2}(\mathcal {V}_{10}\textbf{K}_{6})^{-1}\mho _{1}\big [(\mathcal {V}_{10}\textbf{K}_{6})^{-1}\big ]^{\textbf{T}}~~~~if~~~\vartheta _{6}\ne 0,\\ \end{array}\right. }\nonumber \\{} & {} \Pi _{4}=(\amalg _{14}{\amalg _{22}}{\amalg _{33}}\wp _{4\jmath })^{2}(\mathcal {V}_{11}\textbf{K}_{7})^{-1}\mho _{1}\big [(\mathcal {V}_{11}\textbf{K}_{7})^{-1}\big ]^{\textbf{T}}, \end{aligned}$$

where

83 $$\begin{aligned} \vartheta _{1}{} & {} =({\amalg _{22}}-{\amalg _{41}})(\amalg _{41}+{\amalg _{42}})/{\amalg _{22}},\nonumber \\\vartheta _{2}{} & {} =\vartheta _{1}-{\amalg _{41}-(\amalg _{41}+{\amalg _{42}})}\vartheta _{1}/{\amalg _{33}},\nonumber \\ \vartheta _{3}{} & {} =\big ({\amalg _{41}}{\amalg _{12}}^{2}-{\amalg _{14}}{\amalg _{42}}^{2}+{\amalg _{11}}{\amalg _{12}{\amalg _{42}}}+{\amalg _{31}}{\amalg _{33}}{\amalg _{42}}-{\amalg _{12}}{\amalg _{42}}(\amalg _{41}+{\amalg _{42}})\big )/{\amalg _{12}^{2}},\nonumber \\\vartheta _{4}{} & {} ={\amalg _{33}}({\amalg _{11}}{\amalg _{12}}-{\amalg _{12}}{\amalg _{33}}+{\amalg _{13}}{\amalg _{33}}-{\amalg _{14}}{\amalg _{42}})/{\amalg _{12}}^{2},\nonumber \\ \vartheta _{5}{} & {} =-{\amalg _{14}}{\amalg _{33}}/{\amalg _{12}}+\vartheta _{4}\chi _{3}/\vartheta _{3}-{\amalg _{13}}{\amalg _{42}}\vartheta _{4}^{2}/{\amalg _{12}}\vartheta _{3}^{2},\nonumber \\ \vartheta _{6}{} & {} =({\amalg _{13}}^{2}-{\amalg _{12}}{\amalg _{22}}-{\amalg _{11}}{\amalg _{13}}+{\amalg _{13}}{\amalg _{22}}){\amalg _{22}}/{\amalg _{13}}^{2},\nonumber \\\chi _{3}{} & {} =({\amalg _{13}}-{\amalg _{12}}){\amalg _{22}}{\amalg _{33}}-{\amalg _{14}}{\amalg _{33}}{\amalg _{41}}+{\amalg _{14}}({{\amalg _{41}}}-{\amalg _{22}})({\amalg _{41}}+{\amalg _{42}})\end{aligned}$$

and the matrices $\mathcal {V}_{\varsigma _{1}},~(\varsigma _{1}-1,...,11),~\textbf{K}_{\varsigma _{2}},~(\varsigma _{2}=1,...,7)$ and $\vartheta _{\textbf{s}},~(\textbf{s}=1,...,5)$ are illustrated in the subsequent result.

#### Proof

Case A:  Considering

84 $$\begin{aligned} \digamma _{1}^{2}+\textbf{W}\Pi _{1}+\Pi _{1}\textbf{W}^{\textbf{T}}=0. \end{aligned}$$

In view of the elimination matrix $\textbf{K}_{1}$ as

$$\begin{aligned} \textbf{K}_{1}= \begin{pmatrix} 1&{}00&{}0\\ 0&{}1&{}0&{}0\\ 0&{}0&{}1&{}0\\ 0&{}-{\amalg _{41}}/{\amalg _{22}}&{}0&{}1 \end{pmatrix} \end{aligned}$$

Consequently, we get

$$\begin{aligned} \textbf{W}_{1}=\textbf{K}_{1}\textbf{W}\textbf{K}_{1}^{-1}= \begin{pmatrix} -{\amalg _{11}}&{}{\amalg _{12}}{\amalg _{22}}+{\amalg _{41}}{\amalg _{41}}/{\amalg _{22}}&{}-{\amalg _{13}}&{}{\amalg _{14}}\\ {\amalg _{22}}&{}-{\amalg _{22}}&{}{\amalg _{22}}&{}0\\ 0&{}{\amalg _{33}}&{}-{\amalg _{33}}&{}0\\ 0&{}\vartheta _{1}&{}-{\amalg _{41}}&{}-({\amalg _{41}+{\amalg _{42}}}) \end{pmatrix}, \end{aligned}$$

where $$\vartheta _{1}=({\amalg _{22}}-{\amalg _{41}})({\amalg _{41}}+{\amalg _{42}})/{\amalg _{22}}.$$

The subsequent sub-stages are then taken out of the appropriate evaluation. 

Subphase AI: Choose $\vartheta _{1}=1$ and $\Theta =(0,0,0,1),$ then there is $\mathcal {V}_{1}\textbf{W}_{1}\mathcal {V}_{1}^{-1}=\textbf{W}_{1},$ where $\mathcal {V}_{1}=\big (\Theta \textbf{W}_{1}^{3},\Theta \textbf{W}_{1}^{2},\Theta \textbf{W}_{1},\Theta \big )^{\textbf{T}}$ and

$$\begin{aligned} \textbf{W}_{1}= \begin{pmatrix} -\tilde{a_{1}}&{}-\tilde{a_{2}}&{}-\tilde{a_{3}}&{}-\tilde{a_{4}}\\ 1&{}0&{}0&{}0\\ 0&{}1&{}0&{}0\\ 0&{}0&{}1&{}0 \end{pmatrix}. \end{aligned}$$

Consequently, it is possible to write the appropriate formula of (84) as

$$\begin{aligned} (\mathcal {V}_{1}\textbf{K}_{1})\digamma _{1}^{2}(\mathcal {V}_{1}\textbf{K}_{1})^{\textbf{T}}+\textbf{W}_{1}\big ((\mathcal {V}_{1}\textbf{K}_{1})\Pi _{1}(\mathcal {V}_{1}\textbf{K}_{1})^{\textbf{T}}\big )+\big ((\mathcal {V}_{1}\textbf{K}_{1})\Pi _{1}(\mathcal {V}_{1}\textbf{K}_{1})^{\textbf{T}}\big )\textbf{W}_{1}^{\textbf{T}}=0. \end{aligned}$$

By making the use of Lemma 4.2, we determine $(\mathcal {V}_{1}\textbf{K}_{1})\Pi _{1}(\mathcal {V}_{1}\textbf{K}_{1})^{\textbf{T}}=({\amalg _{22}}{\amalg _{33}}{\amalg _{41}}\wp _{4\jmath -3})^{2}\mho _{1},$ where

$$\begin{aligned} \mho _{1}= \begin{pmatrix} \frac{\tilde{a}_{2}\tilde{a}_{3}-\tilde{a}_{1}\tilde{a}_{4}}{2(\tilde{a}_{1}\tilde{a}_{2}\tilde{a}_{3}-\tilde{a}_{3}^{2}-\tilde{a}_{1}^{2}\tilde{a}_{4})}&{}0&{}-\frac{\tilde{a}_{3}}{2(\tilde{a}_{1}\tilde{a}_{2}\tilde{a}_{3}-\tilde{a}_{3}^{2}-\tilde{a}_{1}^{2}\tilde{a}_{4})}&{}0\\ 0&{}\frac{\tilde{a}_{3}}{2(\tilde{a}_{1}\tilde{a}_{2}\tilde{a}_{3}-\tilde{a}_{3}^{2}-\tilde{a}_{1}^{2}\tilde{a}_{4})}&{}0&{}-\frac{\tilde{a}_{1}}{2(\tilde{a}_{1}\tilde{a}_{2}\tilde{a}_{3}-\tilde{a}_{3}^{2}-\tilde{a}_{1}^{2}\tilde{a}_{4})}\\ -\frac{\tilde{a}_{3}}{2(\tilde{a}_{1}\tilde{a}_{2}\tilde{a}_{3}-\tilde{a}_{3}^{2}-\tilde{a}_{1}^{2}\tilde{a}_{4})}&{}0&{}\frac{\tilde{a}_{31}}{2(\tilde{a}_{1}\tilde{a}_{2}\tilde{a}_{3}-\tilde{a}_{3}^{2}-\tilde{a}_{1}^{2}\tilde{a}_{4})}&{}0\\ 0&{}-\frac{\tilde{a}_{1}}{2(\tilde{a}_{1}\tilde{a}_{2}\tilde{a}_{3}-\tilde{a}_{3}^{2}-\tilde{a}_{1}^{2}\tilde{a}_{4})}&{}0&{}-\frac{\tilde{a}_{1}\tilde{a}_{2}-\tilde{a}_{3}}{2(\tilde{a}_{1}\tilde{a}_{2}\tilde{a}_{3}-\tilde{a}_{3}^{2}-\tilde{a}_{1}^{2}\tilde{a}_{4})} \end{pmatrix} \end{aligned}$$

is a P-D symmetric matrix. Therefore, $\Pi _{1}=({\amalg _{22}}{\amalg _{33}}{\amalg _{41}}\wp _{4\jmath -3})^{2}(\mathcal {V}_{1}\textbf{K}_{1})^{-1}\mho _{1}\big ((\mathcal {V}_{1}\textbf{K}_{1})^{-1}\big )^{\textbf{T}}$ is also a P-D matrix.

Subcase AII: Taking $\vartheta _{1}\ne 0$ and also suppose that $\textbf{W}_{2}=\textbf{K}_{2}\textbf{W}_{1}\textbf{K}_{2}^{-1},$ where

$$\begin{aligned} \textbf{K}_{2}= \begin{pmatrix} 1&{}0&{}0&{}0\\ 0&{}1&{}0&{}0\\ 0&{}0&{}1&{}0\\ 0&{}0&{}-\frac{\vartheta _{1}}{{\amalg _{33}}}&{}1 \end{pmatrix}~~and~~~\textbf{W}_{2}=\textbf{K}_{2}\textbf{W}_{1}\textbf{K}_{2}^{-1}=\begin{pmatrix} -{\amalg _{11}}&{}\frac{{\amalg _{12}}{\amalg _{22}}+{\amalg _{14}}{\amalg _{41}}}{{\amalg _{22}}}&{}\frac{{\amalg _{14}}{\amalg _{41}}-{\amalg _{13}}{\amalg _{22}}{\amalg _{33}}}{{\amalg _{22}}{\amalg _{33}}}&{}{\amalg _{14}}\\ {\amalg _{22}}&{}-{\amalg _{22}}&{}{\amalg _{22}}&{}0\\ 0&{}{\amalg _{33}}&{}-{\amalg _{33}}&{}0\\ 0&{}0&{}\vartheta _{2}&{}-({\amalg _{41}}+\amalg _{42}) \end{pmatrix}, \end{aligned}$$

containing $$\vartheta _{2}=\vartheta _{1}-{\amalg _{41}}-({\amalg _{41}}+{\amalg _{42}})\vartheta _{1}/{\amalg _{33}}.$$

Subcase AIII: Taking $\vartheta _{1}\ne 0$ and $\vartheta _{2}=0.$ Moreover, suppose that $\textbf{W}_{2}=\mathcal {V}_{2}\textbf{W}_{2}\mathcal {V}_{2}^{-1},$ where

$$\begin{aligned} \mathcal {V}_{2}= \begin{pmatrix} {\amalg _{22}}{\amalg _{33}}&{}-{\amalg _{33}}({\amalg _{22}}+{\amalg _{33}})&{}{\amalg _{33}}^{2}+{\amalg _{22}}{\amalg _{33}}&{}0\\ 0&{}{\amalg _{33}}&{}-{\amalg _{33}}&{}0\\ 0&{}0&{}1&{}0\\ 0&{}0&{}0&{}1 \end{pmatrix}~~and~~~\textbf{W}_{2}=\begin{pmatrix} -\chi _{1}&{}-\chi _{2}&{}-\chi _{3}&{}{\amalg _{14}}{\amalg _{22}}{\amalg _{33}}\\ 1&{}0&{}0&{}0\\ 0&{}1&{}0&{}0\\ 0&{}0&{}0&{}-({\amalg _{41}}+\amalg _{42}) \end{pmatrix}, \end{aligned}$$

containing $\chi _{2}={\amalg _{11}}+{\amalg _{22}}+{\amalg _{33}}>0,~\chi _{2}=({\amalg _{11}}-{\amalg _{12}}){\amalg _{22}}+{\amalg _{11}}{\amalg _{33}}-{\amalg _{14}}{\amalg _{41}}>0$ and $\chi _{3}=({\amalg _{13}}-{\amalg _{12}}){\amalg _{22}}{\amalg _{33}}-{\amalg _{14}}{\amalg _{33}}{\amalg _{41}}+{\amalg _{14}}({\amalg _{41}}-{\amalg _{22}})({\amalg _{41}}+{\amalg _{42}})>0.$ Hence, we get

$$\begin{aligned} (\mathcal {V}_{2}\textbf{K}_{2}\textbf{K}_{1})\digamma _{1}^{2}(\mathcal {V}_{2}\textbf{K}_{2}\textbf{K}_{1})^{\textbf{T}}+\textbf{W}_{2}\big ((\mathcal {V}_{2}\textbf{K}_{2}\textbf{K}_{1})\Pi _{1}(\mathcal {V}_{2}\textbf{K}_{2}\textbf{K}_{1})^{\textbf{T}}\big )+\big ((\mathcal {V}_{2}\textbf{K}_{2}\textbf{K}_{1})\Pi _{1}(\mathcal {V}_{2}\textbf{K}_{2}\textbf{K}_{1})^{\textbf{T}}\big )\textbf{W}_{2}^{\textbf{T}}=0. \end{aligned}$$

By making the use of Lemma 4.2, we determine $(\mathcal {V}_{2}\textbf{K}_{2}\textbf{K}_{1})\Pi _{1}(\mathcal {V}_{2}\textbf{K}_{2}\textbf{K}_{1})^{\textbf{T}}=({\amalg _{22}}{\amalg _{33}}{\amalg _{41}}\wp _{4\jmath -3})^{2}\mho _{2},$ where

$$\begin{aligned} \mho _{2}=\begin{pmatrix} \frac{\chi _{2}}{2(\chi _{1}\chi _{2}-\chi _{3})}&{}0&{}-\frac{1}{2(\chi _{1}\chi _{2}-\chi _{3})}&{}0\\ 0&{}\frac{1}{2(\chi _{1}\chi _{2}-\chi _{3})}&{}0&{}0\\ -\frac{1}{2(\chi _{1}\chi _{2}-\chi _{3})}&{}0&{}\frac{\chi _{1}}{2(\chi _{1}\chi _{2}-\chi _{3})}&{}0\\ 0&{}0&{}0&{}0 \end{pmatrix} \end{aligned}$$

is a symmetric, semi P-D matrix. Thus, $\Pi _{1}=({\amalg _{22}}{\amalg _{33}}\wp _{4\jmath -3})^{2}(\mathcal {V}_{2}\textbf{K}_{2}\textbf{K}_{1})^{-1}\mho _{2}\big ((\mathcal {V}_{2}\textbf{K}_{2}\textbf{K}_{1})^{-1}\big )^{\textbf{T}}.$

Subcase AIV: Taking $\vartheta _{1}\ne 0$ and $\vartheta _{2}\ne 0,$ employing the analogous technique as we did in Subcase AI. Suppose that $\mathcal {V}_{3}=(\Theta \textbf{W}_{2}^{3},\Theta \textbf{W}_{2}^{2},\Theta \textbf{W}_{2},\Theta )^{\textbf{T}}$ so that $\mathcal {V}_{3}\textbf{W}_{2}\mathcal {V}_{3}^{-1}=\textbf{W}_{1}.$ Hence, we have $(\mathcal {V}_{3}\textbf{K}_{2}\textbf{K}_{1})\digamma _{1}^{2}(\mathcal {V}_{3}\textbf{K}_{2}\textbf{K}_{1})^{\textbf{T}}+\textbf{W}_{2}\big ((\mathcal {V}_{3}\textbf{K}_{2}\textbf{K}_{1})\Pi _{1}(\mathcal {V}_{3}\textbf{K}_{2}\textbf{K}_{1})^{\textbf{T}}\big )+\big ((\mathcal {V}_{3}\textbf{K}_{2}\textbf{K}_{1})\Pi _{1}(\mathcal {V}_{3}\textbf{K}_{2}\textbf{K}_{1})^{\textbf{T}}\big )\textbf{W}_{3}^{\textbf{T}}=0,$ where $(\mathcal {V}_{3}\textbf{K}_{2}\textbf{K}_{1})\Pi _{1}(\mathcal {V}_{3}\textbf{K}_{2}\textbf{K}_{1})^{\textbf{T}}=({\amalg _{22}}{\amalg _{33}}\vartheta _{2}\wp _{4\jmath -3})^{2}\mho _{1}.$ Thus, we conclude that $\Pi _{1}=({\amalg _{22}}{\amalg _{33}}\vartheta _{2}\wp _{4\jmath -3})^{2}(\mathcal {V}_{3}\textbf{K}_{2}\textbf{K}_{1})^{-1}\mho _{1}\big ((\mathcal {V}_{3}\textbf{K}_{2}\textbf{K}_{1})\big )^{\textbf{T}}$ is a P-D matrix.

Case B: Considering

$$\begin{aligned} \digamma _{2}^{2}+\textbf{W}\Pi _{2}+\Pi _{2}\textbf{W}^{\textbf{T}}=0. \end{aligned}$$

Assume that $\textbf{K}_{3}\textbf{W}\textbf{K}_{3}=\textbf{W}_{3},$ where

$$\begin{aligned}{} & {} \textbf{K}_{3}= \begin{pmatrix} 0&{}1&{}0&{}0\\ 1&{}0&{}0&{}0\\ -{\amalg _{42}}/{\amalg _{12}}&{}0&{}0&{}1\\ -{\amalg _{33}}/{\amalg _{12}}&{}0&{}1&{}0 \end{pmatrix}, \nonumber \\{} & {} \textbf{W}_{3}=\begin{pmatrix} -{\amalg _{22}}&{}{{\amalg _{12}}{\amalg _{22}}+{\amalg _{22}}{\amalg _{33}}}/{{\amalg _{12}}}&{}0&{}{\amalg _{22}}\\ {\amalg _{12}}&{}-\big ({\amalg _{11}}{\amalg _{12}}+{\amalg _{13}}{\amalg _{33}}+{\amalg _{14}}{\amalg _{42}}/{\amalg _{12}}\big )&{}{\amalg _{14}}&{}-{\amalg _{13}}\\ 0&{}\vartheta _{3}&{}-\big ({\amalg _{12}}{\amalg _{41}}+{\amalg _{12}}{\amalg _{42}}+{\amalg _{14}}{\amalg _{42}}/{\amalg _{12}}\big )&{}{\amalg _{13}}{\amalg _{42}}/{\amalg _{12}}\\ 0&{}\vartheta _{4}&{}-{\amalg _{14}}{\amalg _{23}}/{\amalg _{12}}&{}{\amalg _{13}}{\amalg _{33}}-{\amalg _{33}}{\amalg _{12}}/{\amalg _{12}}, \end{pmatrix}, \end{aligned}$$

where $$\vartheta _{3}=\big ({\amalg _{41}}{\amalg _{12}}^{2}-{\amalg _{14}}{\amalg _{42}}^{2}+{\amalg _{11}}{\amalg _{12}}{\amalg _{42}}+{\amalg _{13}}{\amalg _{33}}{\amalg _{42}}-{\amalg _{12}}{\amalg _{42}}({\amalg _{41}}+{\amalg _{42}})/{\amalg _{12}}^{2}\big )$$ and $$\vartheta _{4}={\amalg _{33}}\big ({\amalg _{11}}{\amalg _{12}}-{\amalg _{12}}{\amalg _{33}}+{\amalg _{13}}{\amalg _{33}}-{\amalg _{14}}{\amalg _{42}}/{\amalg _{12}}^{2}\big ).$$

Subcase BI: When $\vartheta _{3}=0=\vartheta _{4}$ and suppose that $\textbf{W}_{3}=\mathcal {V}_{4}\textbf{W}_{3}\mathcal {V}_{4}^{-1},$ where

$$\begin{aligned}{} & {} \mathcal {V}_{4}= \begin{pmatrix} {\amalg _{12}}&{}-\big ({\amalg _{11}}{\amalg _{12}}+{\amalg _{13}}{\amalg _{33}}+{\amalg _{14}}{\amalg _{42}}/{\amalg _{12}}\big )&{}{\amalg _{14}}&{}-{\amalg _{13}}\\ 0&{}1&{}0&{}0\\ 0&{}0&{}1&{}0\\ 0&{}0&{}0&{}1 \end{pmatrix},\nonumber \\ {}{} & {} \textbf{W}_{3}=\begin{pmatrix} -\chi _{4}&{}-\chi _{5}&{}-\chi _{6}&{}-\chi _{7}\\ 1&{}0&{}0&{}0\\ 0&{}0&{}-\big ({\amalg _{12}}({\amalg _{41}}+{\amalg _{42}})+{\amalg _{14}}{\amalg _{42}}/{\amalg _{12}}\big )&{}{\amalg _{13}}{\amalg _{42}}/{\amalg _{12}}\\ 0&{}0&{}-{\amalg _{14}}{\amalg _{33}}/{\amalg _{12}}&{}{\amalg _{13}}{\amalg _{33}}-{\amalg _{33}}{\amalg _{12}}/{\amalg _{12}} \end{pmatrix} \end{aligned}$$

containing $$\chi _{4}=\big ({\amalg _{11}}{\amalg _{12}}+{\amalg _{12}}{\amalg _{22}}+{\amalg _{13}}{\amalg _{33}}-{\amalg _{14}}{\amalg _{42}}/{\amalg _{12}}\big )>0,$$ $$\chi _{5}=\big (({\amalg _{11}}-{\amalg _{12}}){\amalg _{12}}{\amalg _{22}}+({\amalg _{13}}-{\amalg _{12}}){\amalg _{22}}{\amalg _{33}}-{\amalg _{14}}{\amalg _{42}}{\amalg _{22}}/{\amalg _{12}}\big )>0,$$ $$\chi _{6}=\big (({\amalg _{13}}-{\amalg _{12}}){\amalg _{12}}{\amalg _{23}}+({\amalg _{23}}-{\amalg _{12}}){\amalg _{22}}{\amalg _{31}}-{\amalg _{14}}{\amalg _{42}}{\amalg _{23}}/{\amalg _{12}}\big )>0,$$ and $$\chi _{7}=\big (({\amalg _{33}}-{\amalg _{12}}){\amalg _{12}}{\amalg _{13}}+({\amalg _{42}}-{\amalg _{12}}){\amalg _{22}}{\amalg _{13}}-{\amalg _{14}}{\amalg _{42}}{\amalg _{14}}/{\amalg _{12}}\big )>0.$$

In this way, we have

$$\begin{aligned} (\mathcal {V}_{4}\textbf{K}_{3})\digamma _{2}^{2}(\mathcal {V}_{4}\textbf{K}_{3})^{\textbf{T}}+\textbf{W}_{3}\big ((\mathcal {V}_{4}\textbf{K}_{3})\Pi _{2}(\mathcal {V}_{4}\textbf{K}_{3})^{\textbf{T}}\big )+\big ((\mathcal {V}_{4}\textbf{K}_{3})\Pi _{2}(\mathcal {V}_{4}\textbf{K}_{3})^{\textbf{T}}\big )\textbf{W}_{3}^{\textbf{T}}. \end{aligned}$$

Taking into account Lemma 4.2, we have $(\mathcal {V}_{4}\textbf{K}_{3})\Pi _{2}(\mathcal {V}_{4}\textbf{K}_{3})^{\textbf{T}}$ is a semi P-D matrix and

$$\begin{aligned} (\mathcal {V}_{4}\textbf{K}_{3})\Pi _{2}(\mathcal {V}_{4}\textbf{K}_{3})^{\textbf{T}}=({\amalg _{12}}\wp _{4\jmath -2})^{2}\mho _{3} \\ \mho _{3}=\begin{pmatrix} (2\chi _{4})^{-1}&{}0&{}0&{}0\\ 0&{}(2\chi _{4}\chi _{5})^{-1}&{}0&{}0\\ 0&{}0&{}0&{}0\\ 0&{}0&{}0&{}0 \end{pmatrix}. \end{aligned}$$

Finally, $$\Pi _{2}=({\amalg _{12}}\wp _{4\jmath -2})^{2}(\mathcal {V}_{4}\textbf{K}_{3})^{-1}\mho _{3}\big ((\mathcal {V}_{4}\textbf{K}_{3})^{-1}\big )^{\textbf{T}}.$$

Subcase BII: When $\vartheta _{3}\ne 0$ and $\vartheta _{4}=0,$ applying the analogous approach as we did in the Subcase AI, then we attain $\mathcal {V}_{5}=(\Theta \textbf{W}_{3}^{3},\Theta \textbf{W}_{3}^{2},\Theta \textbf{W}_{3},\Theta )^{\textbf{T}}$ so that $\mathcal {V}_{5}\textbf{W}_{3}\mathcal {V}_{5}^{-1}=\textbf{W}_{1}.$ hence, we get

$$\begin{aligned} (\mathcal {V}_{5}\textbf{K}_{3})\digamma _{2}^{2}(\mathcal {V}_{5}\textbf{K}_{3})^{\textbf{T}}+\textbf{W}_{1}\big ((\mathcal {V}_{5}\textbf{K}_{3})\Pi _{2}(\mathcal {V}_{5}\textbf{K}_{3})^{\textbf{T}}\big )+\big ((\mathcal {V}_{5}\textbf{K}_{3})\Pi _{2}(\mathcal {V}_{5}\textbf{K}_{3})^{\textbf{T}}\big )\textbf{W}_{1}^{\textbf{T}}, \end{aligned}$$

where $$(\mathcal {V}_{5}\textbf{K}_{3})\Pi _{2}(\mathcal {V}_{5}\textbf{K}_{3})^{\textbf{T}}=({\amalg _{14}}{\amalg _{33}}\vartheta _{3}\wp _{4\jmath -2})^{2}\mho _{1}.$$ Thus,

85 $$\begin{aligned} \Pi _{2}=({\amalg _{14}}{\amalg _{33}}\vartheta _{3}\wp _{4\jmath -2})^{2}(\mathcal {V}_{5}\textbf{K}_{3})^{-1}\mho _{1}\big ((\mathcal {V}_{5}\textbf{K}_{3})^{-1}\big )^{\textbf{T}} \end{aligned}$$

is a P-D matrix. Subcase BIII: When $\vartheta _{3}=0$ and $\vartheta _{4}\ne 0$ and suppose that $\textbf{W}_{4}=\textbf{K}_{4}\textbf{W}_{3}\textbf{K}_{4}^{-1},$ where

$$\begin{aligned}{} & {} \textbf{K}_{4}= \begin{pmatrix} 1&{}0&{}0&{}0\\ 0&{}1&{}0&{}0\\ 0&{}0&{}0&{}1\\ 0&{}0&{}1&{}0 \end{pmatrix}\nonumber \\{} & {} \textbf{W}_{4}= \begin{pmatrix} -{\amalg _{22}}&{}{\amalg _{12}}{\amalg _{22}}+{\amalg _{22}}{\amalg _{33}}/{\amalg _{12}}&{}{\amalg _{22}}&{}0\\ {\amalg _{12}}&{}-\big ({\amalg _{11}}{\amalg _{12}}+{\amalg _{13}}{\amalg _{33}}+{\amalg _{14}}{\amalg _{42}}/{\amalg _{12}}\big )&{}-{\amalg _{13}}&{}{\amalg _{14}}\\ 0&{}\vartheta _{4}&{}{\amalg _{13}}{\amalg _{33}}-{\amalg _{33}}{\amalg _{12}}/{\amalg _{12}}&{}-{\amalg _{14}}{\amalg _{33}}/{\amalg _{12}}\\ 0&{}0&{}{\amalg _{13}}{\amalg _{42}}/{\amalg _{12}}&{}-\big ({\amalg _{12}}(\amalg _{41}+{\amalg _{42}})+{\amalg _{14}}{\amalg _{42}}/{\amalg _{12}}\big ) \end{pmatrix}. \end{aligned}$$

Furthermore, we have $\mathcal {V}_{6}=(\Theta \textbf{W}_{4}^{3},\Theta \textbf{W}_{4}^{2},\Theta \textbf{W}_{4},\Theta )^{\textbf{T}}$ so that $\mathcal {V}_{6}\textbf{W}_{4}\mathcal {V}_{6}^{-1}=\textbf{W}_{1}.$ Hence, we get

$$\begin{aligned} (\mathcal {V}_{6}\textbf{K}_{4}\textbf{K}_{3})\digamma _{2}^{2}(\mathcal {V}_{6}\textbf{K}_{4}\textbf{K}_{3})^{\textbf{T}}+\textbf{W}_{1}\big ((\mathcal {V}_{6}\textbf{K}_{4}\textbf{K}_{3})\Pi _{2}(\mathcal {V}_{6}\textbf{K}_{4}\textbf{K}_{3}\big )+\big ((\mathcal {V}_{6}\textbf{K}_{4}\textbf{K}_{3})\Pi _{2}(\mathcal {V}_{6}\textbf{K}_{4}\textbf{K}_{3})^{\textbf{T}}\big )\textbf{W}_{1}^{\textbf{T}}, \end{aligned}$$

where $$(\mathcal {V}_{6}\textbf{K}_{4}\textbf{K}_{3})\Pi _{2}(\mathcal {V}_{6}\textbf{K}_{4}\textbf{K}_{3})^{\textbf{T}}=({\amalg _{13}}{\amalg _{42}}\vartheta _{4}\wp _{4\jmath -2})^{2}\mho _{1}.$$ Thus,

$$\begin{aligned} \Pi _{2}=({\amalg _{13}}{\amalg _{42}}\vartheta _{4}\wp _{4\jmath -2})^{2}(\mathcal {V}_{6}\textbf{K}_{4}\textbf{K}_{3})^{-1}\mho _{1}\big ((\mathcal {V}_{6}\textbf{K}_{4}\textbf{K}_{3})^{-1}\big )^{\textbf{T}} \end{aligned}$$

is a P-D matrix. 

Subcase BIV: When $\vartheta _{3}=\vartheta _{4}\ne 0$ and suppose that $\textbf{W}_{5}=\textbf{K}_{5}\textbf{W}_{3}\textbf{K}_{5}^{-1},$ where

$$\begin{aligned}{} & {} \textbf{K}_{5}= \begin{pmatrix} 1&{}0&{}0&{}0\\ 0&{}1&{}0&{}0\\ 0&{}0&{}1&{}0\\ 0&{}0&{}-{\vartheta _{4}}/{\vartheta _{3}}&{}1 \end{pmatrix}\nonumber \\{} & {} \textbf{W}_{5}= \begin{pmatrix} -{\amalg _{22}}&{}{\amalg _{12}}{\amalg _{22}}+{\amalg _{22}}{\amalg _{33}}/{\amalg _{12}}&{}{\amalg _{22}}{\vartheta _{4}}/{\vartheta _{3}}&{}{\amalg _{22}}\\ {\amalg _{12}}&{}-\big ({\amalg _{11}}{\amalg _{12}}+{\amalg _{13}}{\amalg _{33}}+{\amalg _{14}}{\amalg _{42}}/{\amalg _{12}}\big )&{}{\amalg _{14}}-{\amalg _{13}}{\vartheta _{4}}/{\vartheta _{3}}&{}-{\amalg _{13}}\\ 0&{}\vartheta _{4}&{}-\chi _{8}&{}{\amalg _{13}}{\amalg _{42}}/{\amalg _{12}}\\ 0&{}0&{}\vartheta _{5}&{}\frac{\vartheta _{3}(\amalg _{13}\amalg _{33}-\amalg _{33}\amalg _{12})-\amalg _{13}\amalg _{32}\vartheta _{4}}{\vartheta _{3}\amalg _{12}} \end{pmatrix}. \end{aligned}$$

Furthermore, we have $\mathcal {V}_{6}=(\Theta \textbf{W}_{4}^{3},\Theta \textbf{W}_{4}^{2},\Theta \textbf{W}_{4},\Theta )^{\textbf{T}}$ so that $\mathcal {V}_{6}\textbf{W}_{4}\mathcal {V}_{6}^{-1}=\textbf{W}_{1}.$ Hence, we get

$$\begin{aligned} (\mathcal {V}_{6}\textbf{K}_{4}\textbf{K}_{3})\digamma _{2}^{2}(\mathcal {V}_{6}\textbf{K}_{4}\textbf{K}_{3})^{\textbf{T}}+\textbf{W}_{1}\big ((\mathcal {V}_{6}\textbf{K}_{4}\textbf{K}_{3})\Pi _{2}(\mathcal {V}_{6}\textbf{K}_{4}\textbf{K}_{3}\big )+\big ((\mathcal {V}_{6}\textbf{K}_{4}\textbf{K}_{3})\Pi _{2}(\mathcal {V}_{6}\textbf{K}_{4}\textbf{K}_{3})^{\textbf{T}}\big )\textbf{W}_{1}^{\textbf{T}}, \end{aligned}$$

where $$(\mathcal {V}_{6}\textbf{K}_{4}\textbf{K}_{3})\Pi _{2}(\mathcal {V}_{6}\textbf{K}_{4}\textbf{K}_{3})^{\textbf{T}}=({\amalg _{13}}{\amalg _{42}}\vartheta _{4}\wp _{4\jmath -2})^{2}\mho _{1}.$$ Thus,

$$\begin{aligned} \Pi _{2}=({\amalg _{13}}{\amalg _{42}}\vartheta _{4}\wp _{4\jmath -2})^{2}(\mathcal {V}_{6}\textbf{K}_{4}\textbf{K}_{3})^{-1}\mho _{1}\big ((\mathcal {V}_{6}\textbf{K}_{4}\textbf{K}_{3})^{-1}\big )^{\textbf{T}} \end{aligned}$$

is a P-D matrix. 

Subcase BV: When $\vartheta _{3}=\vartheta _{4}\ne 0$ and $\vartheta _{5}=0,$ applying the identical technique from Subcase AIII), we obtain $\mathcal {V}_{7}\textbf{W}_{5}\mathcal {V}_{7}^{-1}=\textbf{W}_{4},$ where

$$\begin{aligned} \mathcal {V}_{7}=\begin{pmatrix} {\amalg _{12}}\vartheta _{4}&{}-\chi _{9}&{}\chi _{10}&{}0 \\ 0&{}\vartheta _{4}&{}-\chi _{8}&{}0 \\ 0&{}0&{}1&{}0 \\ 0&{}0&{}0&{}1 \end{pmatrix}~~\quad ~~\textbf{W}_{4}= \begin{pmatrix} -\chi _{11}&{}-\chi _{12}&{}-\chi _{13}&{}\chi _{14}\\ 1&{}0&{}0&{}\chi _{15}\\ 01&{}1&{}0&{}\chi _{16}\\ 0&{}0&{}0&{}\chi _{17} \end{pmatrix}, \end{aligned}$$

having

$$\begin{aligned}{} & {} \chi _{9}=\Big (\frac{{\amalg _{12}}{\amalg _{11}}+{\amalg _{13}}{\amalg _{33}}-{\amalg _{14}}{\amalg _{42}}}{{\amalg _{12}}}+\chi _{8}\Big ),~\chi _{10}=\chi _{8}^{2}+\Big (\frac{\vartheta _{3}{\amalg _{14}}+{\amalg _{13}}\vartheta _{4}}{\vartheta _{3}}\Big )\vartheta _{4},~~\\{} & {} \chi _{11}= {\left\{ \begin{array}{ll} \Bigg \{\big ({\amalg _{11}}{\amalg _{41}}{\amalg _{12}}^{2}+{\amalg _{12}}{\amalg _{42}}{\amalg _{11}}^{2}-{\amalg _{11}}{\amalg _{14}}{\amalg _{42}}^{2}+{\amalg _{22}}{\amalg _{41}}{\amalg _{12}}^{2}-{\amalg _{14}}{\amalg _{22}}{\amalg _{42}}^{2}\\ \quad +({\amalg _{41}}{\amalg _{12}}^{2}-{\amalg _{14}}{\amalg _{42}}^{2}-{\amalg _{12}}{\amalg _{22}}{\amalg _{42}})({\amalg _{41}}+{\amalg _{42}})-{\amalg _{12}}{\amalg _{42}}({\amalg _{41}}+{\amalg _{42}})^{2}\\ \quad +{\amalg _{11}}{\amalg _{12}}{\amalg _{22}}{\amalg _{42}}+{\amalg _{11}}{\amalg _{13}}{\amalg _{33}}{\amalg _{41}}+{\amalg _{13}}{\amalg _{22}}{\amalg _{33}}{\amalg _{42}}+{\amalg _{13}}{\amalg _{33}}^{2}{\amalg _{42}}\big ){\amalg _{12}}^{2}/\vartheta _{3}\Bigg \}>0, \end{array}\right. } \\{} & {} \chi _{12}= {\left\{ \begin{array}{ll} \Bigg \{{\amalg _{13}}^{2}{\amalg _{33}}^{2}{\amalg _{41}}-{\amalg _{12}}^{2}{\amalg _{41}}^{2}{\amalg _{14}}+{\amalg _{14}}^{2}{\amalg _{42}}^{2}{\amalg _{41}}-{\amalg _{12}}^{3}{\amalg _{22}}{\amalg _{41}}-{\amalg _{11}}{\amalg _{12}}^{2}{\amalg _{22}}{\amalg _{42}}\\ \quad +{\amalg _{11}}{\amalg _{12}}^{2}{\amalg _{22}}{\amalg _{41}}+{\amalg _{11}}^{2}{\amalg _{12}}{\amalg _{22}}{\amalg _{42}}-{\amalg _{11}}{\amalg _{14}}{\amalg _{22}}{\amalg _{42}}^{2}+{\amalg _{12}}{\amalg _{14}}{\amalg _{22}}{\amalg _{42}}^{2}\\ \quad +{\amalg _{11}}{\amalg _{12}}^{2}{\amalg _{41}}({\amalg _{41}}+{\amalg _{42}})-{\amalg _{12}}^{2}{\amalg _{22}}{\amalg _{33}}{\amalg _{41}}-{\amalg _{11}}{\amalg _{12}}{\amalg _{42}}({\amalg _{41}}+{\amalg _{42}})^{2}\\ \quad +{\amalg _{11}}^{2}{\amalg _{12}}{\amalg _{42}}({\amalg _{41}}+{\amalg _{42}})-{\amalg _{13}}{\amalg _{22}}{\amalg _{42}}{\amalg _{33}}^{2}-{\amalg _{11}}{\amalg _{14}}{\amalg _{42}}^{2}(\amalg _{41}+{\amalg _{42}})\\ \quad +{\amalg _{14}}{\amalg _{22}}{\amalg _{33}}{\amalg _{42}}^{2}+{\amalg _{12}}^{2}{\amalg _{22}}{\amalg _{42}}({\amalg _{41}}+{\amalg _{42}})+{\amalg _{12}}^{2}{\amalg _{22}}{\amalg _{41}}(\amalg _{41}+{\amalg _{42}})\\ \quad -{\amalg _{12}}{\amalg _{22}}{\amalg _{42}}(\amalg _{41}+{\amalg _{42}})^{2}-{\amalg _{14}}{\amalg _{22}}{\amalg _{42}}^{2}(\amalg _{41}+{\amalg _{42}})-{\amalg _{13}}{\amalg _{33}}{\amalg _{42}}(\amalg _{41}+{\amalg _{42}})^{2}\\ \quad +{\amalg _{11}}{\amalg _{12}}{\amalg _{13}}{\amalg _{33}}{\amalg _{41}}-{\amalg _{11}}{\amalg _{12}}{\amalg _{14}}{\amalg _{42}}{\amalg _{41}}-{\amalg _{11}}{\amalg _{12}}{\amalg _{22}}{\amalg _{33}}{\amalg _{42}}+{\amalg _{11}}{\amalg _{13}}{\amalg _{22}}{\amalg _{33}}{\amalg _{42}}\\ \quad -{\amalg _{12}}{\amalg _{13}}{\amalg _{22}}{\amalg _{33}}{\amalg _{42}}+{\amalg _{12}}{\amalg _{13}}{\amalg _{22}}{\amalg _{33}}{\amalg _{41}}-{\amalg _{12}}{\amalg _{13}}{\amalg _{33}}{\amalg _{33}}{\amalg _{41}}+{\amalg _{11}}{\amalg _{13}}{\amalg _{33}}{\amalg _{42}}({\amalg _{41}}+{\amalg _{42}})\\ \quad +{\amalg _{12}}{\amalg _{13}}{\amalg _{33}}{\amalg _{41}}({\amalg _{41}}+{\amalg _{42}})-2{\amalg _{13}}{\amalg _{14}}{\amalg _{33}}{\amalg _{41}}{\amalg _{42}}+{\amalg _{13}}{\amalg _{22}}{\amalg _{33}}{\amalg _{33}}{\amalg _{42}}+{\amalg _{12}}{\amalg _{14}}{\amalg _{41}}{\amalg _{42}}({\amalg _{41}}+{\amalg _{42}})\\ \quad +{\amalg _{12}}{\amalg _{22}}{\amalg _{33}}{\amalg _{42}}({\amalg _{41}}+{\amalg _{42}})+{\amalg _{13}}{\amalg _{33}}{\amalg _{33}}{\amalg _{42}}({\amalg _{41}}+{\amalg _{42}}){\amalg _{12}}^{2}/\vartheta _{3}\Bigg \}>0, \end{array}\right. }\\{} & {} \chi _{13}= {\left\{ \begin{array}{ll}-\Bigg \{\big ({\amalg _{11}}^{2}{\amalg _{22}}{\amalg _{12}}{\amalg _{33}}{\amalg _{42}}-{\amalg _{11}}^{2}{\amalg _{22}}{\amalg _{12}}{\amalg _{42}}({\amalg _{41}}+{\amalg _{42}})+{\amalg _{12}}^{2}{\amalg _{11}}{\amalg _{22}}{\amalg _{33}}{\amalg _{41}}-{\amalg _{12}}^{2}{\amalg _{11}}{\amalg _{22}}{\amalg _{41}}({\amalg _{41}}+{\amalg _{42}})\\ \quad +{\amalg _{12}}^{2}{\amalg _{11}}{\amalg _{22}}{\amalg _{42}}({\amalg _{41}}+{\amalg _{42}})-{\amalg _{22}}{\amalg _{11}}{\amalg _{12}}{\amalg _{13}}{\amalg _{33}}{\amalg _{41}}-{\amalg _{22}}{\amalg _{11}}{\amalg _{12}}{\amalg _{13}}{\amalg _{33}}{\amalg _{42}}+{\amalg _{22}}{\amalg _{11}}{\amalg _{12}}{\amalg _{14}}{\amalg _{41}}{\amalg _{42}}\\ \quad +{\amalg _{22}}{\amalg _{11}}{\amalg _{12}}{\amalg _{14}}{\amalg _{42}}^{2}-{\amalg _{22}}{\amalg _{33}}{\amalg _{11}}{\amalg _{12}}{\amalg _{33}}{\amalg _{42}}+{\amalg _{22}}{\amalg _{11}}{\amalg _{12}}{\amalg _{13}}{\amalg _{42}}({\amalg _{41}}+{\amalg _{42}})^{2}+{\amalg _{22}}{\amalg _{11}}{\amalg _{13}}{\amalg _{33}}^{2}{\amalg _{42}}\\ \quad -{\amalg _{22}}{\amalg _{11}}{\amalg _{13}}{\amalg _{33}}{\amalg _{42}}({\amalg _{41}}+{\amalg _{42}})-{\amalg _{22}}{\amalg _{11}}{\amalg _{14}}{\amalg _{33}}{\amalg _{42}}^{2}+{\amalg _{22}}{\amalg _{11}}{\amalg _{14}}{\amalg _{42}}^{2}({\amalg _{41}}+{\amalg _{42}})+{\amalg _{22}}{\amalg _{12}}^{3}{\amalg _{41}}({\amalg _{41}}\\ \quad +{\amalg _{42}})+{\amalg _{22}}{\amalg _{12}}^{2}{\amalg _{14}}{\amalg _{41}}^{2}+{\amalg _{22}}{\amalg _{14}}{\amalg _{41}}{\amalg _{42}}{\amalg _{12}}^{2}+{\amalg _{22}}{\amalg _{12}}^{2}{\amalg _{33}}{\amalg _{41}}({\amalg _{41}}+{\amalg _{42}})-{\amalg _{22}}{\amalg _{33}}{\amalg _{12}}^{2}{\amalg _{33}}{\amalg _{41}}\\ \quad -{\amalg _{22}}{\amalg _{42}}{\amalg _{12}}^{2}({\amalg _{41}}+{\amalg _{42}})^{2}+{\amalg _{22}}{\amalg _{12}}{\amalg _{13}}{\amalg _{41}}{\amalg _{33}}^{2}-{\amalg _{22}}{\amalg _{12}}{\amalg _{13}}{\amalg _{33}}{\amalg _{41}}({\amalg _{41}}+{\amalg _{42}})+{\amalg _{22}}{\amalg _{33}}{\amalg _{12}}{\amalg _{13}}{\amalg _{33}}{\amalg _{41}}\\ \quad +{\amalg _{22}}{\amalg _{12}}{\amalg _{13}}{\amalg _{33}}{\amalg _{42}}({\amalg _{41}}+{\amalg _{42}})+{\amalg _{22}}{\amalg _{33}}{\amalg _{12}}{\amalg _{13}}{\amalg _{33}}{\amalg _{42}}-{\amalg _{22}}{\amalg _{12}}{\amalg _{14}}{\amalg _{41}}{\amalg _{42}}({\amalg _{41}}+{\amalg _{42}})\\ \quad -2{\amalg _{22}}{\amalg _{12}}{\amalg _{14}}{\amalg _{42}}^{2}({\amalg _{41}}+{\amalg _{42}})-{\amalg _{22}}{\amalg _{12}}{\amalg _{33}}{\amalg _{42}}({\amalg _{41}}+{\amalg _{42}})^{2}+{\amalg _{22}}{\amalg _{13}}{\amalg _{33}}{\amalg _{42}}({\amalg _{41}}+{\amalg _{42}})^{2}\\ \quad -{\amalg _{22}}{\amalg _{33}}{\amalg _{13}}{\amalg _{33}}{\amalg _{42}}({\amalg _{41}}+{\amalg _{42}})-{\amalg _{22}}{\amalg _{41}}{\amalg _{22}}{\amalg _{14}}^{4}{\amalg _{42}}^{2}{\amalg _{42}}^{3}-{\amalg _{22}}{\amalg _{14}}{\amalg _{33}}{\amalg _{42}}^{2}({\amalg _{41}}\\ \quad +{\amalg _{42}})+{\amalg _{22}}{\amalg _{33}}{\amalg _{14}}{\amalg _{33}}{\amalg _{42}}^{2}\big ){\amalg _{12}}^{2}/\vartheta _{3} \Bigg \}>0. \end{array}\right. } \end{aligned}$$

Moreover, $\chi 13-\chi _{11}\chi _{12}<0,~\chi _{14},~\chi _{15},\chi _{16}$ and $\chi _{17}$ will be determined later.

 Then, we obtain

$$\begin{aligned} (\mathcal {V}_{7}\textbf{K}_{5}\textbf{K}_{3})\digamma _{2}^{2}(\mathcal {V}_{7}\textbf{K}_{5}\textbf{K}_{3})^{\textbf{T}}+\textbf{W}_{4}\big ((\mathcal {V}_{7}\textbf{K}_{5}\textbf{K}_{3})\Pi _{2}(\mathcal {V}_{7}\textbf{K}_{5}\textbf{K}_{3}\big )+\big ((\mathcal {V}_{7}\textbf{K}_{5}\textbf{K}_{3})\Pi _{2}(\mathcal {V}_{7}\textbf{K}_{5}\textbf{K}_{3})^{\textbf{T}}\big )\textbf{W}_{4}^{\textbf{T}}=0, \end{aligned}$$

where $$(\mathcal {V}_{7}\textbf{K}_{5}\textbf{K}_{3})\Pi _{2}(\mathcal {V}_{7}\textbf{K}_{5}\textbf{K}_{3})^{\textbf{T}}=({\amalg _{12}}\vartheta _{4}\wp _{4\jmath -2})^{2}\mho _{4}$$ and

$$\begin{aligned} \mho _{4}= \begin{pmatrix} \big (\chi _{12}/2(\chi _{11}\chi _{12}-\chi _{13})\big )&{}0&{}-\big (1/2(\chi _{11}\chi _{12}-\chi _{13})\big )&{}0\\ 0&{}\big (1/2(\chi _{11}\chi _{12}-\chi _{13})\big )&{}0&{}0\\ -\big (1/2(\chi _{11}\chi _{12}-\chi _{13})\big )&{}0&{}\big (\chi _{11}/2(\chi _{11}\chi _{12}-\chi _{13})\big )&{}0\\ 0&{}0&{}0&{}0 \end{pmatrix}. \end{aligned}$$

Therefore, $$\Pi _{2}=({\amalg _{12}}\vartheta _{4}\wp _{4\jmath -2})^{2}(\mathcal {V}_{7}\textbf{K}_{5}\textbf{K}_{3})^{-1}\mho _{4}\big ((\mathcal {V}_{7}\textbf{K}_{5}\textbf{K}_{3})^{-1}\big )^{\textbf{T}}.$$

Subcase BVI: If $\vartheta _{3}=\vartheta _{4}=\vartheta _{5}\ne 0$ and applying the analogous approach as we did in Subcase AIV. Assume that $\mathcal {V}_{8}=\big (\Theta \textbf{W}_{5}^{3},\Theta \textbf{W}_{5}^{2},\Theta \textbf{W}_{5},\Theta \big )^{\textbf{T}}$ so that $\mathcal {V}_{8}\textbf{W}_{5}\textbf{W}_{8}^{-1}=\textbf{W}_{1}.$ Hence, we have

$$\begin{aligned} (\mathcal {V}_{8}\textbf{K}_{5}\textbf{K}_{3})\digamma _{2}^{2}(\mathcal {V}_{8}\textbf{K}_{5}\textbf{K}_{3})^{\textbf{T}}+\textbf{W}_{1}\big ((\mathcal {V}_{8}\textbf{K}_{5}\textbf{K}_{3})\Pi _{2}(\mathcal {V}_{8}\textbf{K}_{5}\textbf{K}_{3}\big )+\big ((\mathcal {V}_{8}\textbf{K}_{5}\textbf{K}_{3})\Pi _{2}(\mathcal {V}_{8}\textbf{K}_{5}\textbf{K}_{3})^{\textbf{T}}\big )\textbf{W}_{1}^{\textbf{T}}=0, \end{aligned}$$

where $(\mathcal {V}_{8}\textbf{K}_{5}\textbf{K}_{3})\Pi _{2}(\mathcal {V}_{8}\textbf{K}_{5}\textbf{K}_{3})^{\textbf{T}}=({\amalg _{12}}\vartheta _{4}\vartheta _{5}\wp _{4\jmath -2})^{2}\mho _{1}=0.$Therefore, $\Pi _{2}=({\amalg _{12}}\vartheta _{4}\vartheta _{5}\wp _{4\jmath -2})^{2}(\mathcal {V}_{8}\textbf{K}_{5}\textbf{K}_{3})^{-1}\mho _{1}\big ((\mathcal {V}_{8}\textbf{K}_{5}\textbf{K}_{3})^{-1}\big )^{\textbf{T}}$ is a P-D matrix.

Case C:  Surmise that $\digamma _{3}^{2}+\textbf{W}\Pi _{3}+\Pi _{3}\textbf{W}^{\textbf{T}}=0.$

Assume that $\textbf{W}_{6}=\textbf{K}_{6}\textbf{W}\textbf{K}_{6}^{-1},$ where

$$\begin{aligned} \textbf{K}_{6}= \begin{pmatrix} 0&{}0&{}1&{}0\\ 1&{}0&{}0&{}0\\ {{\amalg _{22}}}/{\amalg _{13}}&{}1&{}0&{}0\\ 0&{}0&{}0&{}1 \end{pmatrix} \end{aligned}$$

and

$$\begin{aligned} \textbf{W}_{6}=\begin{pmatrix} -{\amalg _{33}}&{}-{\amalg _{22}}{\amalg _{33}}/{{\amalg _{13}}}&{}{\amalg _{33}}&{}0\\ -{\amalg _{13}}&{}-\big ({\amalg _{11}}{\amalg _{13}}+{\amalg _{12}}{\amalg _{22}}/{\amalg _{13}}\big )&{}{\amalg _{12}}&{}{\amalg _{14}}\\ 0&{}\vartheta _{6}&{}\big ({\amalg _{12}}{\amalg _{22}}-{\amalg _{22}}{\amalg _{12}}/{\amalg _{13}}\big )&{}{\amalg _{14}}{\amalg _{22}}/{\amalg _{13}}\\ 0&{}0&{}{\amalg _{42}}&{}-({\amalg _{41}}+{\amalg _{42}}) \end{pmatrix} \end{aligned}$$

having $$\vartheta _{6}=\big ({\amalg _{13}}^{2}-{\amalg _{12}}{\amalg _{22}}-{\amalg _{11}}{\amalg _{13}}+{\amalg _{13}}{\amalg _{22}}\big ){\amalg _{22}}/{\amalg _{13}}^ {2}.$$

Subcase CI: When $\vartheta _{6}=0,$ employing the identical approach as we applied in Subcase AIII and consider that $\textbf{W}_{5}=\mathcal {V}_{9}\textbf{W}_{6}\mathcal {V}_{9}^{-1},$ where

86 $$\begin{aligned} \mathcal {V}_{9}=\begin{pmatrix} -{\amalg _{13}}&{}-\big ({\amalg _{11}}{\amalg _{13}}+{\amalg _{12}}{\amalg _{22}}/{\amalg _{13}}\big )&{}{\amalg _{12}}&{}{\amalg _{14}}\\ 0&{}1&{}0&{}0\\ 0&{}0&{}1&{}0\\ 0&{}0&{}0&{}1 \end{pmatrix} \end{aligned}$$

and

87 $$\begin{aligned} \textbf{W}_{5}=\begin{pmatrix} -\chi _{18}&{}-\chi _{19}&{}-\chi _{20}&{}-\chi _{22}\\ 1&{}0&{}0&{}0\\ 0&{}0&{}\big ({\amalg _{12}}{\amalg _{22}}-{\amalg _{22}}{\amalg _{13}}/{\amalg _{13}}\big )&{}{\amalg _{14}}{\amalg _{22}}/{\amalg _{13}}\\ 0&{}0&{}{\amalg _{42}}&{}-({\amalg _{41}}+{\amalg _{42}}) \end{pmatrix} \end{aligned}$$

containing $\chi _{18}=({\amalg _{11}}{\amalg _{13}}+{\amalg _{12}}{\amalg _{22}}+{\amalg _{13}}{\amalg _{33}})/{\amalg _{13}},~~\chi _{19}=({\amalg _{11}}{\amalg _{13}}{\amalg _{33}}+{\amalg _{12}}{\amalg _{22}}{\amalg _{33}}-{\amalg _{13}}{\amalg _{33}}{\amalg _{22}})/{\amalg _{13}},~~\chi _{20}$ and $\chi _{22}$ will be computed later.

Thus, we find

$$\begin{aligned} (\mathcal {V}_{9}\textbf{K}_{6})\digamma _{3}^{2}(\mathcal {V}_{9}\textbf{K}_{6})^{\textbf{T}}+\textbf{W}_{5}\big ((\mathcal {V}_{9}\textbf{K}_{6})\Pi _{3}(\mathcal {V}_{9}\textbf{K}_{6})^{\textbf{T}}\big )+\big ((\mathcal {V}_{9}\textbf{K}_{6})\Pi _{3}(\mathcal {V}_{9}\textbf{K}_{6})^{\textbf{T}}\big )\textbf{W}_{5}^{\textbf{T}}=0. \end{aligned}$$

Using the fact of Lemma 4.2, we have $(\mathcal {V}_{9}\textbf{K}_{6})\Pi _{3}(\mathcal {V}_{9}\textbf{K}_{6})^{\textbf{T}}=({\amalg _{13}}\wp _{4\jmath -1})^{2}\mho _{5},$ where

$$\begin{aligned} \mho _{5}=\begin{pmatrix}(2\chi _{18})^{-1}&{}0&{}0&{}0\\ 0&{}(2\chi _{18}\chi _{19})^{-1}&{}0&{}0\\ 0&{}0&{}0&{}0\\ 0&{}0&{}0&{}0 \end{pmatrix}. \end{aligned}$$

Consequently, $$\Pi _{3}=({\amalg _{13}}\wp _{4\jmath -1})^{2}(\mathcal {V}_{9}\textbf{K}_{6})^{-1}\mho _{5}\big ((\mathcal {V}_{9}\textbf{K}_{6})^{-1}\big )^{\textbf{T}}.$$

Subcase CII: When $\vartheta _{6}\ne 0,$ then applying Subcase AI with a similar technique, resulting in $\mathcal {V}_{10}=\big (\Theta \textbf{W}_{6}^{3},\Theta \textbf{W}_{6}^{2},\Theta \textbf{W}_{6},\Theta \big )^{\textbf{T}}$ so that $\mathcal {V}_{10}\textbf{W}_{6}\mathcal {V}_{10}^{-1}=\textbf{W}_{1},$ which leads to

$$\begin{aligned} (\mathcal {V}_{10}\textbf{K}_{6})\digamma _{3}^{2}(\mathcal {V}_{10}\textbf{K}_{6})^{\textbf{T}}+\textbf{W}_{1}\big ((\mathcal {V}_{10}\textbf{K}_{6})\Pi _{3}(\mathcal {V}_{10}\textbf{K}_{6})^{\textbf{T}}\big )+\big ((\mathcal {V}_{10}\textbf{K}_{6})\Pi _{3}(\mathcal {V}_{10}\textbf{K}_{6})^{\textbf{T}}\big )\textbf{W}_{1}^{\textbf{T}}=0, \end{aligned}$$

where

$$\begin{aligned} \big ((\mathcal {V}_{10}\textbf{K}_{6})\Pi _{3}(\mathcal {V}_{10}\textbf{K}_{6})^{\textbf{T}}\big )\Pi _{3}\big ((\mathcal {V}_{10}\textbf{K}_{6})\Pi _{3}(\mathcal {V}_{10}\textbf{K}_{6})^{\textbf{T}}\big )^{\textbf{T}}=({\amalg _{13}}{\amalg _{42}}\vartheta _{6}\wp _{4\jmath -1})^{2}\mho _{1}. \end{aligned}$$

Hence, we conclude that $\Pi _{3}=({\amalg _{13}}{\amalg _{42}}\vartheta _{6}\wp _{4\jmath -1})^{2}(\mathcal {V}_{10}\textbf{K}_{6})^{-1}\mho _{1}\big ((\mathcal {V}_{10}\textbf{K}_{6})^{-1}\big )^{\textbf{T}}$ is a P-D matrix. Case D: Considering $\digamma _{4}^{2}+\textbf{W}\Pi _{4}+\Pi _{4}\textbf{W}^{\textbf{T}}=0,$ and also, we have $\textbf{W}_{7}=\textbf{K}_{7}\textbf{W}\textbf{K}_{7}^{-1},$ where

$$\begin{aligned} \textbf{K}_{7}= \begin{pmatrix} 0&{}0&{}0&{}1\\ 1&{}0&{}0&{}0\\ 0&{}1&{}0&{}0\\ 0&{}0&{}1&{}0 \end{pmatrix}~~\textbf{W}_{7}= \begin{pmatrix} -(\amalg _{41}+{\amalg _{42}})&{}{\amalg _{41}}&{}{\amalg _{42}}&{}0\\ {\amalg _{41}}&{}-{\amalg _{11}}&{}{\amalg _{12}}&{}-{\amalg _{13}}\\ 0&{}{\amalg _{22}}&{}-{\amalg _{22}}&{}{\amalg _{22}}\\ 0&{}0&{}{\amalg _{33}}&{}-{\amalg _{33}} \end{pmatrix}. \end{aligned}$$

Indicate $\mathcal {V}_{11}=\big (\Theta \textbf{W}_{7}^{3},\Theta \textbf{W}_{7}^{2},\Theta \textbf{W}_{7},\Theta \big )^{\textbf{T}}$ so that $\mathcal {V}_{11}\textbf{W}_{7}\mathcal {V}_{11}^{-1}=\textbf{W}_{1}.$ Thus, we get

$$\begin{aligned} (\mathcal {V}_{11}\textbf{K}_{7})\digamma _{4}^{2}(\mathcal {V}_{11}\textbf{K}_{7})^{\textbf{T}}+\mathcal {\mathbb {W}_{1}}\big ((\mathcal {V}_{11}\textbf{K}_{7})\Pi _{4}(\mathcal {V}_{11}\textbf{K}_{7})^{\textbf{T}}\big )+\big ((\mathcal {V}_{11}\textbf{K}_{7})\Pi _{4}(\mathcal {V}_{11}\textbf{K}_{7})^{\textbf{T}}\big )\textbf{W}_{1}^{\textbf{T}}=0, \end{aligned}$$

where

$$\begin{aligned} (\mathcal {V}_{11}\textbf{K}_{7})\Pi _{4}\big ((\mathcal {V}_{11}\textbf{K}_{7})\big )^{\textbf{T}}=({\amalg _{14}}{\amalg _{22}}{\amalg _{33}}\wp _{4\jmath })^{2}\mho _{1}. \end{aligned}$$

This concludes that $\Pi _{4}=({\amalg _{14}}{\amalg _{22}}{\amalg _{33}}\wp _{4\jmath })^{2}(\mathcal {V}_{11}\textbf{K}_{7})^{-1}\mho _{1}\big ((\mathcal {V}_{11}\textbf{K}_{7})^{-1}\big )^{\textbf{T}}$ is a P-D matrix. Finally, the expression $\Pi =\Pi _{\ell },~(\ell =1,...,4)$ is a P-D matrix. So, the solution $\big ({\textbf{S}}(\tau ),{\textbf{E}}_{\textbf{C}}(\tau ),{\textbf{I}}_{\textbf{C}}(\tau ),{\textbf{R}}(\tau )\big )$ of model (68) possess a log-normal P.D.F $\mathcal {Q}(\tilde{\Phi })$ about $\mathcal {V}_{\jmath }^{*}$ as

$$\begin{aligned} \mathcal {Q}(\tilde{\Phi })=\frac{1}{4\varpi _{2}^{2}}\vert \Pi \vert ^{-1/2}\exp \Big (\frac{-1}{2}\tilde{\Phi }\Pi ^{-1}\tilde{\Phi }^{\textbf{T}}\Big ). \end{aligned}$$

This yields the intended result. $\square$

## Numerical procedure for H/A-P model using random perturbations

The computation methods of stochastic perturbations influence whenever differentiating expressions involve fractional differential compositions involving singular or nonsingular kernels, and classical prescriptions include this component. The fractional notions have an order corresponding to 0 and 1.

### Caputo fractional derivative operator

The main objective of this study is to investigate the co-infection of the H/A-P models involving integer-order (4), power-law (5) and stochastic strategy for (16). This scheme incorporates substantial HIV-positive individuals; *Pseudomonas aeruginosa* and *Staphylococcus* aureus are increasingly identified as pneumonia caused by community-based pathogens. Recurrent HIV/AIDS, concomitant pulmonary illness, neutropenia, corticosteroid medication, and serious food insecurity are warning signs for *Staphylococcus*. In the situation where $\intercal$ is chosen as the final propagation period, the mathematical framework will be built using the classical-order expression in the beginning, the power-law memory considered in the next step, and the stochastic configuration in the stages that follow. After the fact that the subsequent number pattern is provided to explain the incidence.

 Specifically, we analyze the sectionally divided frameworks (4), (5) and (16) quantitatively by using the procedure given in^35^ in the context of CFD. In order to outline the procedure, we conducted what follows:

$$\begin{aligned} {\left\{ \begin{array}{ll} \frac{d\textbf{U}_{\jmath }(\textbf{t})}{d\textbf{t}}=\Psi (\textbf{t},\textbf{U}_{\jmath }).~\textbf{U}_{\jmath }(0)=\textbf{U}_{\jmath ,0},~\jmath =1,2,...,\mathfrak {n}~if~\textbf{t}\in [0,\intercal _{1}],\\ \,_{\intercal _{1}}^{c}\textbf{D}_{\textbf{t}}^{\nu }\textbf{U}_{\jmath }(\textbf{t})=\Psi (\textbf{t},\textbf{U}_{\jmath }),~\textbf{U}_{\jmath }(\intercal _{1})=\textbf{U}_{\jmath ,1},~if~\textbf{t}\in [\intercal _{1},\intercal _{2}],\\ d\textbf{U}_{\jmath }(\textbf{t})=\Psi (\textbf{t},\textbf{U}_{\jmath })d\textbf{t}+\wp _{\jmath }\textbf{U}_{\jmath }d\mathbb {W}_{\jmath }(\textbf{t}),~\textbf{U}_{\jmath }(\intercal _{2})=\textbf{U}_{\jmath ,2},~if~\textbf{t}\in [\intercal _{2},\intercal ]. \end{array}\right. } \end{aligned}$$

Thus, it implies that

$$\begin{aligned}{} & {} \textbf{U}_{\jmath }^{\textbf{w}}= {\left\{ \begin{array}{ll}\textbf{U}_{\jmath }(0)+\sum \limits _{\iota =2}^{\textbf{w}}\Big \{\frac{23}{12}\Psi ({\textbf{t}}_{\iota },\textbf{U}^{\iota })\Delta \textbf{t}-\frac{4}{3}\Psi ({\textbf{t}}_{\iota -1},\textbf{U}^{\iota -1})\Delta \textbf{t}+\frac{7}{12}\Psi ({\textbf{t}}_{\iota -2},\textbf{U}^{\iota -2})\Delta \textbf{t}\Big \},~~\textbf{t}\in [0,\intercal _{1}].\\ \textbf{U}_{\jmath }(\intercal _{1})+\frac{(\Delta \textbf{t})^{\nu -1}}{\Gamma (\nu +1)}\sum \limits _{\iota =2}^{\textbf{w}}\Psi ({\textbf{t}}_{\iota -2},\textbf{U}^{\iota -2})\widetilde{\mathfrak {I}_{1}}\\ \quad + \frac{(\Delta \textbf{t})^{\nu -1}}{\Gamma (\nu +2)}\sum \limits _{\iota =2}^{\textbf{w}}\Big \{\Psi ({\textbf{t}}_{\iota -1},\textbf{U}^{\iota -1})-\Psi ({\textbf{t}}_{\iota -2},\textbf{U}^{\iota -2})\Big \}\widetilde{\mathfrak {I}_{2}}\\ \quad +\frac{\nu (\Delta \textbf{t})^{\nu -1}}{2\Gamma (\nu +3)}\sum \limits _{\iota =2}^{\textbf{w}}\Big \{\Psi ({\textbf{t}}_{\iota },\textbf{U}^{\iota })-2\Psi ({\textbf{t}}_{\iota -1},\textbf{U}^{\iota -1})+\Psi ({\textbf{t}}_{\iota -2},\textbf{U}^{\iota -2})\Big \}\widetilde{\mathfrak {I}_{3}},~~\textbf{t}\in [\intercal _{1},\intercal _{2}],\\ \textbf{U}_{\jmath }(\intercal _{2})+\sum \limits _{\iota =\textbf{w}+3}^{\mathfrak {n}}\Big \{\frac{7}{12}\Psi ({\textbf{t}}_{\iota -2},\textbf{U}^{\iota -2})\Delta \textbf{t}-\frac{4}{3}\Psi ({\textbf{t}}_{\iota -1},\textbf{U}^{\iota -1})\Delta \textbf{t}+\frac{23}{12}\Psi ({\textbf{t}}_{\iota },\textbf{U}^{\iota })\Delta \textbf{t}\Big \} \\ \quad +\sum \limits _{\iota =\textbf{w}+3}^{\mathfrak {n}}\Big \{\frac{7}{12}\big (\mathbb {W}({\textbf{t}}_{\iota -1})-\mathbb {W}({\textbf{t}}_{\iota -2})\big )\wp \textbf{U}^{\iota -2}- \frac{4}{3}\big (\mathbb {W}({\textbf{t}}_{\iota })-\mathbb {W}({\textbf{t}}_{\iota -1})\big )\wp \textbf{U}^{\iota -1}\\ \quad +\frac{23}{12}\big (\mathbb {W}({\textbf{t}}_{\iota +1})-\mathbb {W}({\textbf{t}}_{\iota })\big )\wp \textbf{U}^{\iota }\Big \},~~\textbf{t}\in [\intercal _{2},\intercal ],\end{array}\right. } \end{aligned}$$

where

88 $$\begin{aligned}{} & {} \widetilde{\mathfrak {I}_{1}}:=(\textbf{w}-\iota -1)^{\nu }-(\textbf{w}-\iota )^{\nu }, \end{aligned}$$

89 $$\begin{aligned}{} & {} \widetilde{\mathfrak {I}_{2}}:=(\textbf{w}-\iota +1)^{\nu }(\textbf{w}-\iota +2\nu +3)-(\textbf{w}-\iota )^{\nu }(\textbf{w}-\iota +3\nu +3) \end{aligned}$$

and

90 $$\begin{aligned} \widetilde{\mathfrak {I}_{3}}:= {\left\{ \begin{array}{ll}(\textbf{w}-\iota +1)^{\nu }\Big (2(\textbf{w}-\iota )^{2}+ (3\nu +10)(\textbf{w}-\iota )+2\nu ^{2}+9\nu +12\Big )\\ \quad + (\textbf{w}-\iota )^{\nu }\Big (2(\textbf{w}-\iota )^{2}+(5\nu +10)(\textbf{w}-\iota )+6\nu ^{2}+18\nu +12\Big ). \end{array}\right. } \end{aligned}$$

### Caputo-Fabrizio fractional derivative operator

The aim of this research is to examine the co-infection of the H/A-P models using integer-order (4), exponential decay kernel (6) and the ensuing stochastic scheme (16). Prominent individuals with HIV are included in this approach; pneumonia triggered by founded infections is typically associated with *Pseudomonas aeruginosa* and *Staphylococcus* infections. Alarm signals for *Staphylococcus* include persistent HIV/AIDS, concurrent pulmonary illnesses, neutropenia, corticosteroid medication, and toxic consumption. The mathematical structure will be constructed using the classical-order formulation at first, the exponential decay memory at a later stage, and the stochastic setting in the phases that proceed in the case when $\intercal$ is selected as the ultimate dissemination time. Following this, the following numerical pattern is given to clarify this occurrence.

At this point, we examine the sequential configurations (4), (6) and (16) analytically by using the method outlined in^35^ in the context of the CFFD. In order to lay out the procedure, we did what follows:

91 $$\begin{aligned} {\left\{ \begin{array}{ll} \frac{d\textbf{U}_{\jmath }(\textbf{t})}{d\textbf{t}}=\Psi (\textbf{t},\textbf{U}_{\jmath }).~\textbf{U}_{\jmath }(0)=\textbf{U}_{\jmath ,0},~\jmath =1,2,...,\mathfrak {n}~if~\textbf{t}\in [0,\intercal _{1}],\\ \,_{\intercal _{1}}^{CF}\textbf{D}_{\textbf{t}}^{\nu }\textbf{U}_{\jmath }(\textbf{t})=\Psi (\textbf{t},\textbf{U}_{\jmath }),~\textbf{U}_{\jmath }(\intercal _{1})=\textbf{U}_{\jmath ,1},~if~\textbf{t}\in [\intercal _{1},\intercal _{2}],\\ d\textbf{U}_{\jmath }(\textbf{t})=\Psi (\textbf{t},\textbf{U}_{\jmath })d\textbf{t}+\wp _{\jmath }\textbf{U}_{\jmath }d\mathbb {W}_{\jmath }(\textbf{t}),~\textbf{U}_{\jmath }(\intercal _{2})=\textbf{U}_{\jmath ,2},~if~\textbf{t}\in [\intercal _{2},\intercal ]. \end{array}\right. } \end{aligned}$$

Thus, it implies that

92 $$\begin{aligned} \textbf{U}_{\jmath }^{\textbf{w}}= {\left\{ \begin{array}{ll} \textbf{U}_{\jmath }(0)+\sum \limits _{\iota =2}^{\textbf{w}}\Big \{\frac{23}{12}\Psi ({\textbf{t}}_{\iota },\textbf{U}^{\iota })\Delta \textbf{t}-\frac{4}{3}\Psi ({\textbf{t}}_{\iota -1},\textbf{U}^{\iota -1})\Delta \textbf{t}+\frac{7}{12}\Psi ({\textbf{t}}_{\iota -2},\textbf{U}^{\iota -2})\Delta \textbf{t}\Big \},~~\textbf{t}\in [0,\intercal _{1}].\\ \textbf{U}_{\jmath }(\intercal _{1})+\frac{1-\nu }{\mathbb {M}(\nu )}\Psi ({\textbf{t}}_{\mathfrak {n}},\textbf{U}^{\mathfrak {n}})+\frac{\nu }{\mathbb {M}(\nu )}\sum \limits _{\iota =2}^{\textbf{w}}\Big \{\frac{7}{12}\Psi ({\textbf{t}}_{\iota -2},\textbf{U}^{\iota -2})\Delta \textbf{t}-\frac{4}{3}\Psi ({\textbf{t}}_{\iota -1},\textbf{U}^{\iota -1})\Delta \textbf{t}\\ \quad +\frac{23}{12}\Psi ({\textbf{t}}_{\iota },\textbf{U}^{\iota })\Delta \textbf{t}\Big \},~~\textbf{t}\in [\intercal _{1},\intercal _{2}],\\ \textbf{U}_{\jmath }(\intercal _{2})+\sum \limits _{\iota =\textbf{w}+3}^{\mathfrak {n}}\Big \{\frac{7}{12}\Psi ({\textbf{t}}_{\iota -2},\textbf{U}^{\iota -2})\Delta \textbf{t}-\frac{4}{3}\Psi ({\textbf{t}}_{\iota -1},\textbf{U}^{\iota -1})\Delta \textbf{t}+\frac{23}{12}\Psi ({\textbf{t}}_{\iota },\textbf{U}^{\iota })\Delta \textbf{t}\Big \} \\ \quad +\sum \limits _{\iota =\textbf{w}+3}^{\mathfrak {n}}\Big \{\frac{7}{12}\big (\mathbb {W}({\textbf{t}}_{\iota -1})-\mathbb {W}({\textbf{t}}_{\iota -2})\big )\wp \textbf{U}^{\iota -2}- \frac{4}{3}\big (\mathbb {W}({\textbf{t}}_{\iota })-\mathbb {W}({\textbf{t}}_{\iota -1})\big )\wp \textbf{U}^{\iota -1}\\ \quad +\frac{23}{12}\big (\mathbb {W}({\textbf{t}}_{\iota +1})-\mathbb {W}({\textbf{t}}_{\iota })\big )\wp \textbf{U}^{\iota }\Big \},~~\textbf{t}\in [\intercal _{2},\intercal ].\end{array}\right. } \end{aligned}$$

### Atangana–Baleanu–Caputo fractional derivative operator

The current research aims to investigate the co-infection of the stochastic technique (16) and the integer-order model (4) and the GML kernel H/A-P models (7). This plan includes significant HIV-positive individuals; *Pseudomonas aeruginosa* and *Staphylococcus* aureus are usually identified as pneumonia caused by community-based pathogens. Recurrent HIV/AIDS, concomitant pulmonary illness, neutropenia, corticosteroid medication, and hazardous intakes are warning signs for *Staphylococcus*. Initially, the classical-order interpretation will be used to build the computational framework; afterwards, the GML function will be implemented; and in the phases that follow, the stochastic configuration will be used in the scenario where $\intercal$ is chosen as the eventual propagation time. After that, the subsequent numerical structure is provided to explain these instances. In particular, we analyze the sequential configurations (4), (7) and (16) numerically employing the algorithm defined in^35^ in the framework of the ABCFD. In order to lay out the procedure, we did what follows:

$$\begin{aligned} {\left\{ \begin{array}{ll} \frac{d\textbf{U}_{\jmath }(\textbf{t})}{d\textbf{t}}=\Psi (\textbf{t},\textbf{U}_{\jmath }).~\textbf{U}_{\jmath }(0)=\textbf{U}_{\jmath ,0},~\jmath =1,2,...,\mathfrak {n}~if~\textbf{t}\in [0,\intercal _{1}],\\ \,_{\intercal _{1}}^{ABC}\textbf{D}_{\textbf{t}}^{\nu }\textbf{U}_{\jmath }(\textbf{t})=\Psi (\textbf{t},\textbf{U}_{\jmath }),~\textbf{U}_{\jmath }(\intercal _{1})=\textbf{U}_{\jmath ,1},~if~\textbf{t}\in [\intercal _{1},\intercal _{2}],\\ d\textbf{U}_{\jmath }(\textbf{t})=\Psi (\textbf{t},\textbf{U}_{\jmath })d\textbf{t}+\wp _{\jmath }\textbf{U}_{\jmath }d\mathbb {W}_{\jmath }(\textbf{t}),~\textbf{U}_{\jmath }(\intercal _{2})=\textbf{U}_{\jmath ,2},~if~\textbf{t}\in [\intercal _{2},\intercal ]. \end{array}\right. } \end{aligned}$$

Thus, it implies that

$$\begin{aligned} \textbf{U}_{\jmath }^{\textbf{w}}={\left\{ \begin{array}{ll}\textbf{U}_{\jmath }(0)+\sum \limits _{\iota =2}^{\textbf{w}}\Big \{\frac{23}{12}\Psi ({\textbf{t}}_{\iota },\textbf{U}^{\iota })\Delta \textbf{t}-\frac{4}{3}\Psi ({\textbf{t}}_{\iota -1},\textbf{U}^{\iota -1})\Delta \textbf{t}+\frac{7}{12}\Psi ({\textbf{t}}_{\iota -2},\textbf{U}^{\iota -2})\Delta \textbf{t}\Big \},~~\textbf{t}\in [0,\intercal _{1}].\\ \textbf{U}_{\jmath }(\intercal _{1})+\frac{1-\nu }{ABC(\nu )}\Psi ({\textbf{t}}_{\mathfrak {n}},\textbf{U}^{\mathfrak {n}})+\frac{\nu (\Delta \textbf{t})^{\nu -1}}{ABC(\nu )\Gamma (\nu +1)}\sum \limits _{\iota =2}^{\textbf{w}}\Psi ({\textbf{t}}_{\iota -2},\textbf{U}^{\iota -2})\widetilde{\mathfrak {I}_{1}}\\ \quad + \frac{\nu (\Delta \textbf{t})^{\nu -1}}{ABC(\nu )\Gamma (\nu +2)}\sum \limits _{\iota =2}^{\textbf{w}}\Big \{\Psi ({\textbf{t}}_{\iota -1},\textbf{U}^{\iota -1})-\Psi ({\textbf{t}}_{\iota -2},\textbf{U}^{\iota -2})\Big \}\widetilde{\mathfrak {I}_{2}}\\ \quad +\frac{\nu (\Delta \textbf{t})^{\nu -1}}{2ABC(\nu )\Gamma (\nu +3)}\sum \limits _{\iota =2}^{\textbf{w}}\Big \{\Psi ({\textbf{t}}_{\iota },\textbf{U}^{\iota })-2\Psi ({\textbf{t}}_{\iota -1},\textbf{U}^{\iota -1})+\Psi ({\textbf{t}}_{\iota -2},\textbf{U}^{\iota -2})\Big \}\widetilde{\mathfrak {I}_{3}},~~\textbf{t}\in [\intercal _{1},\intercal _{2}],\\ \textbf{U}_{\jmath }(\intercal _{2})+\sum \limits _{\iota =\textbf{w}+3}^{\mathfrak {n}}\Big \{\frac{7}{12}\Psi ({\textbf{t}}_{\iota -2},\textbf{U}^{\iota -2})\Delta \textbf{t}-\frac{4}{3}\Psi ({\textbf{t}}_{\iota -1},\textbf{U}^{\iota -1})\Delta \textbf{t}+\frac{23}{12}\Psi ({\textbf{t}}_{\iota },\textbf{U}^{\iota })\Delta \textbf{t}\Big \} \\ \quad +\sum \limits _{\iota =\textbf{w}+3}^{\mathfrak {n}}\Big \{\frac{7}{12}\big (\mathbb {W}({\textbf{t}}_{\iota -1})-\mathbb {W}({\textbf{t}}_{\iota -2})\big )\wp \textbf{U}^{\iota -2}- \frac{4}{3}\big (\mathbb {W}({\textbf{t}}_{\iota })-\mathbb {W}({\textbf{t}}_{\iota -1})\big )\wp \textbf{U}^{\iota -1}\\ \quad +\frac{23}{12}\big (\mathbb {W}({\textbf{t}}_{\iota +1})-\mathbb {W}({\textbf{t}}_{\iota })\big )\wp \textbf{U}^{\iota }\Big \},~~\textbf{t}\in [\intercal _{2},\intercal ],\end{array}\right. } \end{aligned}$$

where the previous values of $\widetilde{\mathfrak {I}_{1}}, ~\widetilde{\mathfrak {I}_{2}}$, and $\widetilde{\mathfrak {I}_{3}}$ are found in (88)-(90).

### Experimental outcomes and discussion

In order to support research ideas, we will demonstrate mathematical simulation techniques in the next part that make leverage of the Atangana and Araz approaches formerly mentioned in^35^. The appropriateness and usefulness of the planned H/A-P are demonstrated through a number of concrete instances, such as manpower reductions, delays in test result transformation, and limitations of analytical equipment. The accessibility and promptness of HIV/AIDS examinations have been severely impacted by these interruptions in the deterministic-probabilistic situation. Utilizing MATLAB 21, all quantitative and symbolic computations were performed. 

**Figure 2:**
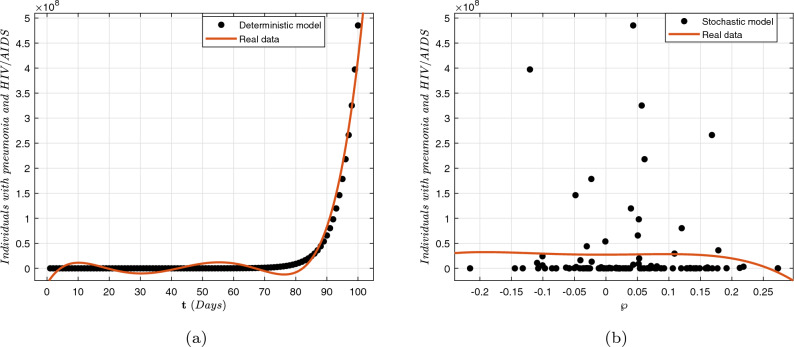
**(a)** Fitting of the proposed deterministic model to real data, **(b)** Fitting of the proposed stochastic model to real data.

Researchers are at present demonstrating a great deal of enthusiasm in the estimation of modeling characteristics from provided statistical information, and it is thought to be an essential component of quantitative disease investigations. The aforementioned section was added to the current investigation employing the popular nonlinear least squares method. Applying the previously described method, the settings were determined, and the structure was calibrated to actual codynamic situations found in Later research from the Philippines and South Africa revealed that, for a specific duration, HIV/AIDS patients having pneumonia had a 2.17^57^ and 2.7^28^ worse probability of death, respectively, than pneumonia individuals lacking HIV/AIDS^59^. For the fitting of the model to data, available records for daily reported HIV/AIDS cases in^59^ and pneumonia^57^ between March 2021 (the initial incidence had been identified on March 18, 2021) and July 2022 are used. The fitting shown in Fig. 2a, b reveals that our perturbed models behaves very well with the data via deterministic and stochastic case.

Especially, the entire number of documented infections and fatalities over the time span between March 2021 (the initial incidence had been identified on March 18, 2021) and July 2022 were used to determine the characteristics of the model. Considering the implementation of (93), the Ordinary Least Square solution was employed to reduce the inaccuracy concepts, and the associated relative deviation is incorporated in assessing the quality of fit as

93 $$\begin{aligned} \min \bigg \{\frac{\sum \limits _{\Bbbk =1}^{\mathfrak {n}}({\Im _{1}}_{\Bbbk }-\hat{{\Im _{1}}}_{\Bbbk })^{2}}{\sum \limits _{\Bbbk =1}^{\mathfrak {n}}{\Im _{1}}^{2}_{\Bbbk }}\bigg \}. \end{aligned}$$

The documented accumulative infection rates are denoted by ${\Im _{1}}_{\Bbbk }$ in this particular instance, while the total number of contaminated occurrences determined by modeling execution is denoted by $\hat{{\Im _{1}}}_{\Bbbk }$. The people who are moved daily from the contaminated compartment to the confined compartment are added together to determine the estimated levels of progressive transmission. With the exception of $\sigma =0.05$, which is envisioned, all the parameters are estimates. The parameters’ projected estimates are displayed in Table 2.

**Table 2:** Details on the system’s characteristic.

Notations	Values	References
$\nabla$	1000 person/day	^57^
$\eta$	4.247637e$^{-5}$	^58^
$\varphi _{1}$	0.005/day	^59^
$\varphi _{2}$	0.0004/day	^59^
$\eta _{1}$	0.00034/day	^59^
$\eta _{2}$	0.057/day	^60^
$\eta _{3}$	0.15/day	Supposed
${\Im _{2}}$	0.0021/day	Supposed
$\varphi _{5}$	1/no unit	Supposed
$\varphi _{6}$	1/no unit	Supposed
$\omega _{1}$	1/no unit	Supposed
$\varphi _{3}$	1/no unit	Supposed
$\varphi _{4}$	0.023/day	^61^
$\varphi _{7}$	0.13/day	^60^
$\zeta$	0.048	Estimated
${\Im _{1}}$	0.1/day	^58^
$\delta _{1}$	0.343/day	^60^
$\delta _{2}$	0.0115/day	^59^
$\textbf{m}_{1}$	0.597/day	^59^
$\textbf{m}_{2}$	0.006/day	^59^
$\delta _{3}$	0.2/day	^60^
$\beta$	0.46	Estimated
$\omega$	0.75	Estimated

#### Example 5.1

To illustrate our results, we quantitatively generate the paths for the probabilistic sickness structures (4), (5) and (16) and their corresponding deterministic components. The starting points are $({\textbf{S}}(0),{\textbf{P}}_{\textbf{m}_{1}}(0),{\textbf{H}}_{\textbf{m}_{1}}(0),{\textbf{H}}(0),{\textbf{H}}_{\textbf{X}}(0),{\textbf{P}}(0),{\textbf{Y}}(0),{\textbf{P}}_{\textbf{T}}(0),{\textbf{T}}_{\textbf{H}\textbf{X}}(0))=(1500,350,250,150,100,200,90,85,70)$ and the time range is [0, 200] units. Table 2 allows us to re-select the parameters to represent the piecewise methodology assessment of the naturally occurring factor process for (4), (5) and (16), respectively. Here, we calculate the fundamental reproductive quantity $\mathbb {R}_{0}=1.8945>1$ for the deterministic framework (4), which suggests that co-infection of H/A-P will continue to exist in the average situation in both submodels. To observe how noise concentration affects the behavior of the probabilistic framework (16), we select random perturbations $\wp _{\jmath }=0.07~(\jmath =1,...,4).$ This yields $\mathbb {R}_{0}^{\mathbb {S}}=\frac{\chi _{\textbf{H}}\widetilde{\textbf{S}}+\varphi _{3}\chi _{\textbf{H}}\widetilde{{\textbf{P}}_{\textbf{m}_{1}}}}{\eta +\tau +\varphi _{4}+\varphi _{5}\chi _{\textbf{q}}+\frac{\wp _{4}^{2}}{2}}=2.1045>1.$ The existence of an ESD for the probabilistic model (16) is demonstrated by Theorem 3.2.

**Figure 3:**
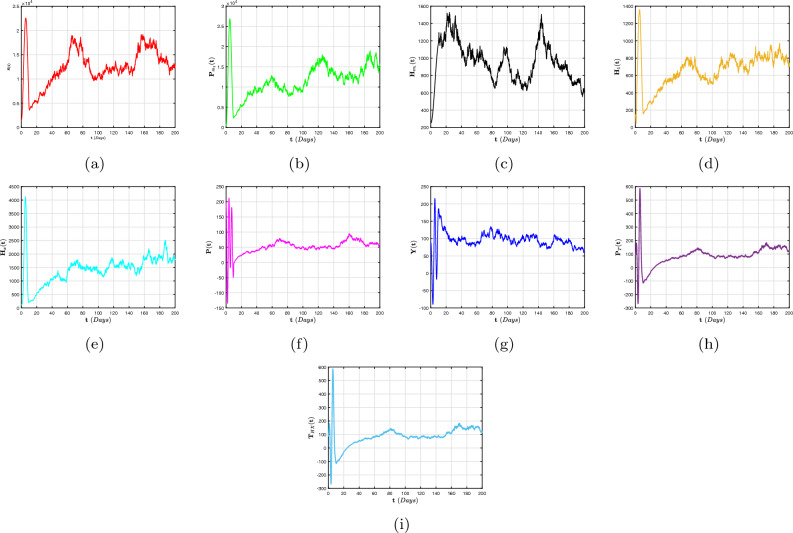
Time evaluation plots for deterministic-probabilistic co-infection H/A-P models (4), (5) and (16) with the impacts antiretroviral therapy and post-exposure prophylaxis using CFD operator having FO $\omega =0.96,$ low intensities and ICs (1500, 350, 250, 150, 100, 200, 90, 85, 70).

**Figure 4:**
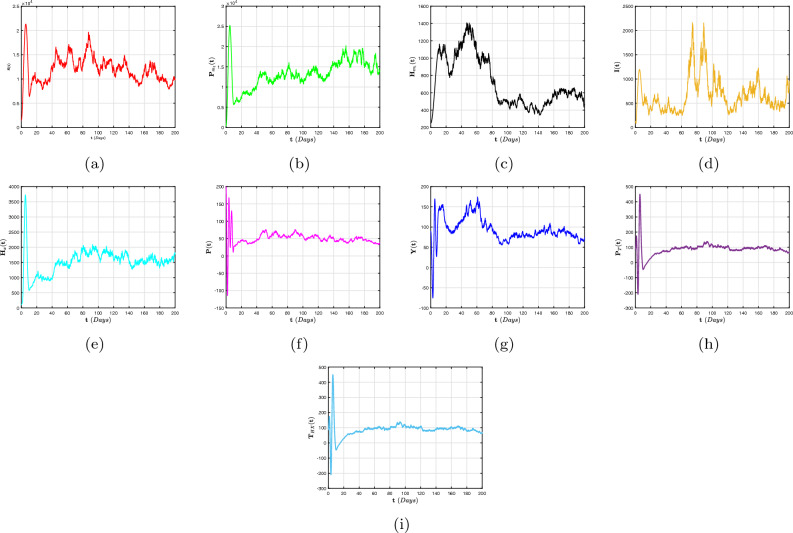
Time evaluation plots for deterministic-probabilistic co-infection H/A-P models (4), (5) and (16) with the impacts antiretroviral therapy and post-exposure prophylaxis using CFFD operator having FO $\omega =0.96,$ low intensities and ICs (1500, 350, 250, 150, 100, 200, 90, 85, 70).

**Figure 5:**
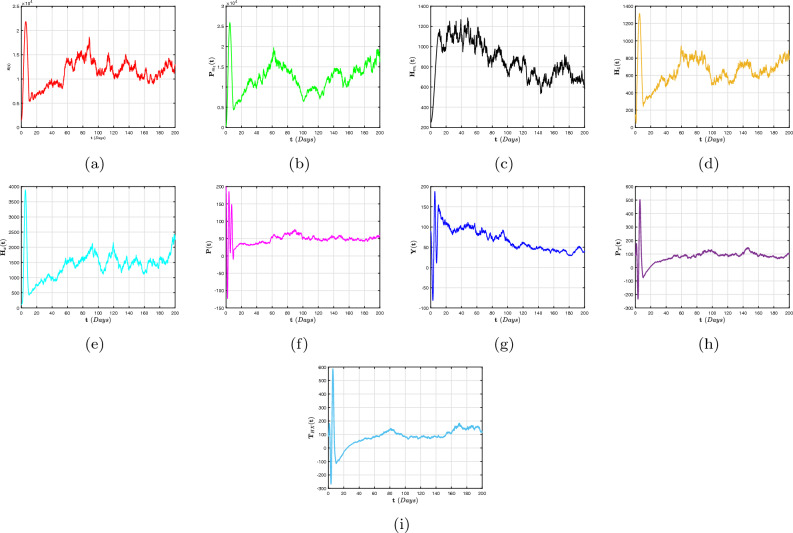
Time evaluation plots for deterministic-probabilistic co-infection H/A-P models (4), (5) and (16) with the impacts antiretroviral therapy and post-exposure prophylaxis using ABCFD operator having FO $\omega =0.96,$ low intensities and ICs (1500, 350, 250, 150, 100, 200, 90, 85, 70).

**Figure 6:**
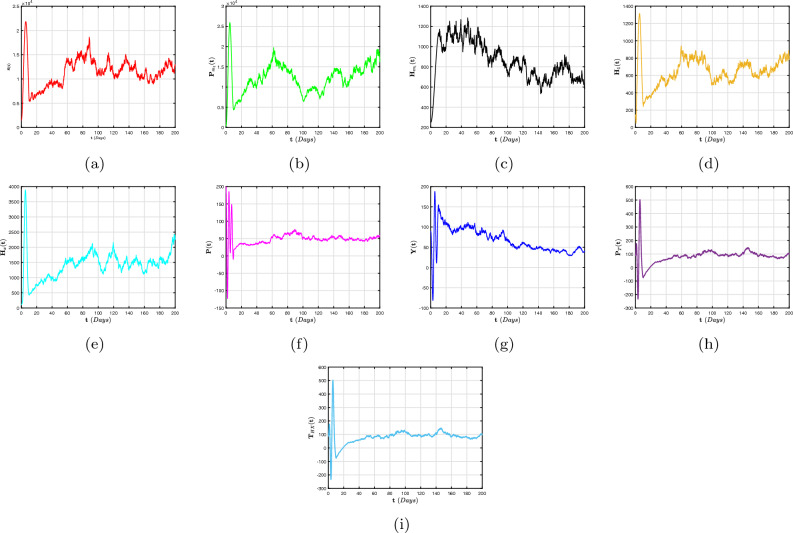
Time evaluation plots for deterministic-probabilistic co-infection H/A-P models (4), (5) and (16) with the impacts antiretroviral therapy and post-exposure prophylaxis using CFD operator having FO $\omega =0.98,$ low intensities and ICs (1000, 500, 150, 100, 150, 75, 85, 70, 60).

**Figure 7:**
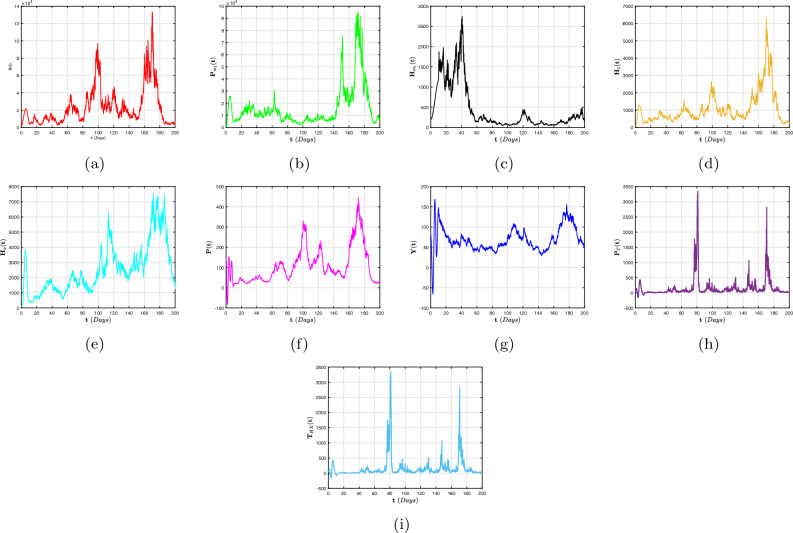
Time evaluation plots for deterministic-probabilistic co-infection H/A-P models (4), (5) and (16) with the impacts antiretroviral therapy and post-exposure prophylaxis using CFFD operator having FO $\omega =0.98,$ low intensities and ICs (1000, 500, 150, 100, 150, 75, 85, 70, 60).

**Figure 8:**
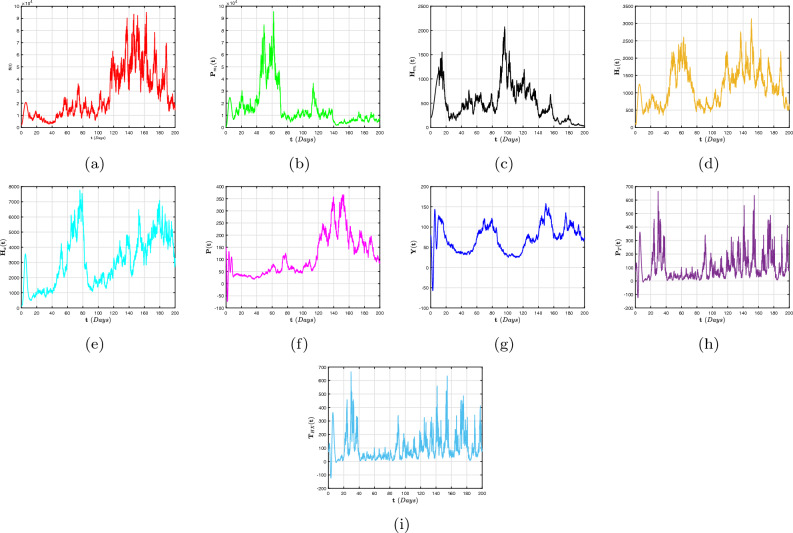
Time evaluation plots for deterministic-probabilistic co-infection H/A-P models (4), (5) and (16) with the impacts antiretroviral therapy and post-exposure prophylaxis using ABCFD operator having FO $\omega =0.98,$ low intensities and ICs (1000, 500, 150, 100, 150, 75, 85, 70, 60).

When a power-law-type kernel with a FO $\omega =0.96$ is employed on (5)-(7), Figs. 3, 4, 5a–i illustrates how incorporating two propagation interprets enhances the occurrence of ailments in comparison to a single procedure. In view of the CFD, CFFD, ABCFD operators and biological-nature strategy, we also find that certain combinations prove more deadly than others. A transmission surge is produced by all interactions comprising the effective collaboration rate connecting ${\textbf{S}}$ and ${\textbf{P}}_{\textbf{m}_{1}}$. This is followed by any coupling via the effective interface rate between $\textbf{S}$ and ${\textbf{P}}_{\textbf{m}_{1}}$, and finally other procedures. We employ the identical factors as in (5)-(7), when implementing the identical methodology to the fractional derivative operators of DEs (4), (6) and (16), respectively. The respective two ICs $({\textbf{S}}(0),{\textbf{P}}_{\textbf{m}_{1}}(0), {\textbf{H}}_{\textbf{m}_{1}}(0),{\textbf{H}}(0),{\textbf{H}}_{\textbf{X}}(0),{\textbf{P}}(0),{\textbf{Y}}(0),{\textbf{P}}_{\textbf{T}}(0),{\textbf{T}}_{\textbf{H}\textbf{X}}(0))=(1000,500,150,100,150,75,85,70,60)$ and $({\textbf{S}}(0),{\textbf{P}}_{\textbf{m}_{1}} (0),{\textbf{H}}_{\textbf{m}_{1}}(0),{\textbf{H}}(0),{\textbf{H}}_{\textbf{X}}(0),{\textbf{P}}(0),{\textbf{Y}}(0),{\textbf{P}}_{\textbf{T}}(0),{\textbf{T}}_{\textbf{H}\textbf{X}}(0))=(1000,600,250,150,90,130,80,75,65)$ are depicted in Figs. 6a–i and 7a–i, with various population schemes. It is simple to compute the threshold factors $\mathbb {R}_{0}^{\mathbb {S}}>1$ and $\mathbb {R}_{{\textbf{H}\textbf{P}}}^{0}<1.$ As seen in Figures 8 (**a**-**i**), the co-infections are expected to continue in a typical way, supporting the result of Theorem 3.2 (see Figs. 7a–i and 8a–i). According to this research, co-infection will spread throughout the body and develop ineffective causative agents, whereas prospective antiretroviral therapy and preventive medication regimens may be altered by the development of additional HIV medications and short-term therapies for opportunistic illnesses such as cryptococcal meningitis, which may include intravenous compositions.

**Figure 9:**
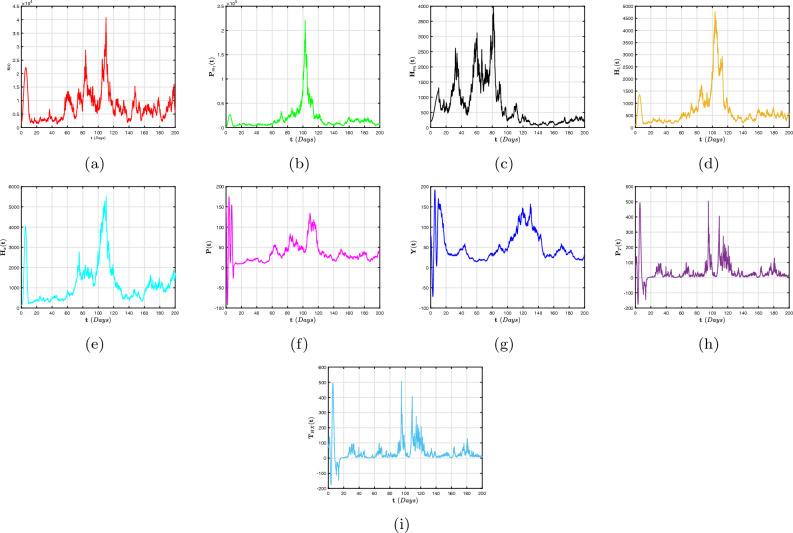
Time evaluation plots for deterministic-probabilistic co-infection H/A-P models (4), (5) and (16) with the impacts antiretroviral therapy and post-exposure prophylaxis using CFD operator having FO $\omega =0.98,$ low intensities and ICs (1000, 600, 250, 150, 90, 130, 80, 75, 65).

**Figure 10:**
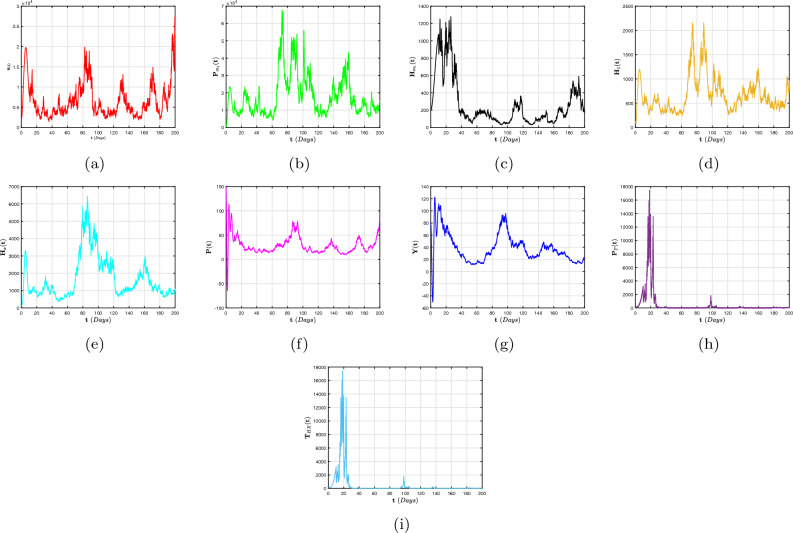
Time evaluation plots for deterministic-probabilistic co-infection H/A-P models (4), (5) and (16) with the impacts antiretroviral therapy and post-exposure prophylaxis using CFFD operator having FO $\omega =0.98,$ low intensities and ICs (1000, 600, 250, 150, 90, 130, 80, 75, 65).

**Figure 11:**
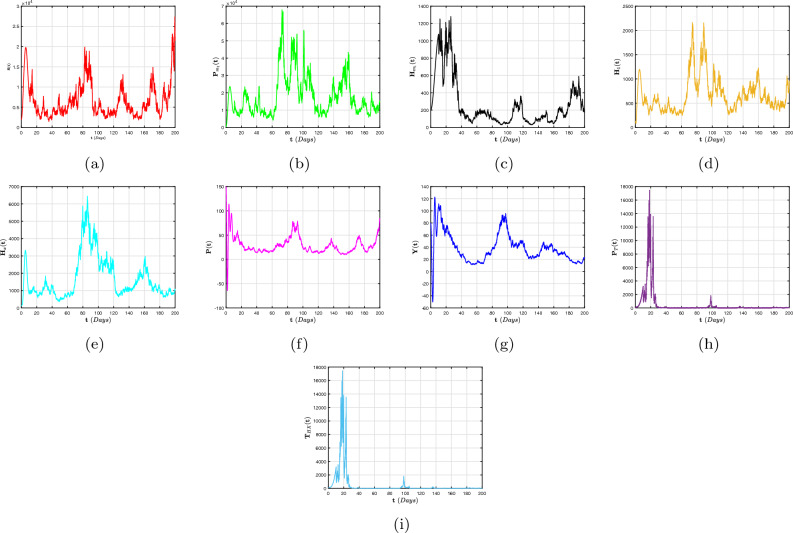
Time evaluation plots for deterministic-probabilistic co-infection H/A-P models (4), (5) and (16) with the impacts antiretroviral therapy and post-exposure prophylaxis using ABCFD operator having FO $\omega =0.98,$ low intensities and ICs (1000, 600, 250, 150, 90, 130, 80, 75, 65).

In light of the prevalent concentrations and parameterization fluctuations discussed above, an intervention plan based on the computational findings for (4), (7) and (16) seems to be effective. There is an ESD of a probabilistic framework (16), as shown by Theorems 3.2. These results suggest that co-infection will become increasingly permanent while pneumonia will go extinct in CFFD and ABCFD cases. These are corroborated by Figs. 9a–i, 10a–i and 11a–i, respectively.

#### Example 5.2

For probabilistic co-infection systems (16) involving community propagation, it is challenging to define appropriate criteria for virus extermination considering the limits of statistical approaches. Nonetheless, we provide a numerical model of the disappearance of illnesses where the noise is high for a thorough explanation. For instance, in the actual environment, individuals haphazardly raise vaccination or exterminating rates to stop co-infection from spreading. This successfully removes contamination.

**Figure 12:**
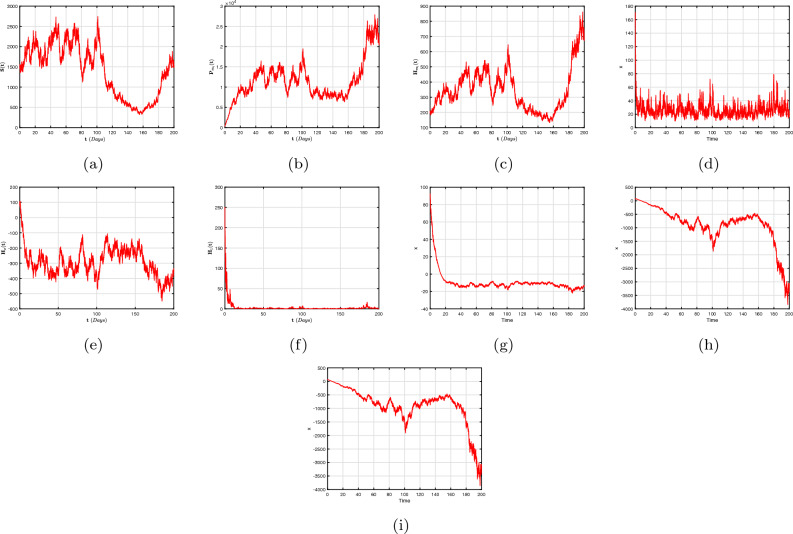
Time evaluation plots for deterministic-probabilistic co-infection H/A-P models (4), (5) and (16) with the impacts antiretroviral therapy and post-exposure prophylaxis using ABCFD operator having FO $\omega =0.98,$ low intensities and ICs (1500, 350, 250, 150, 100, 200, 90, 85, 70).

To illustrate that high levels of environmental disturbance will eventually cause pneumonia to disappear, we set $\wp _{1}=\wp _{2}=0.41,$ $\wp _{\kappa }=0.01,~\kappa =3,4,5,6,7,8,9$ with the identical setting off rate as well as additional factors as in the aforesaid discussion. Following this, as Fig. 12a–i illustrates, co-infection will become extinct.

#### Example 5.3

For the probabilistic pneumonia model in the absence of HIV/AIDS (68), the white noise $\wp _{\jmath }=0.01,~(\jmath =1,...,9)$ and the IC and the remaining arguments are the similar as in Example 5.1. Thus, we determine $\mathbb {R}_{0}^{\kappa }=1.8943>1.$ and the quasi-equilibrium $({\textbf{S}}_{\jmath }^{*},{{\textbf{P}}_{\textbf{m}_{1}}}_{\jmath }^{*},{\textbf{P}}_{\jmath }^{*},{{\textbf{P}}_{\textbf{T}}}_{\jmath }^{*})=(901670.79, 702301.67, 2034892.65, 1568923.23).$ In view of Theorem 4.3.

94 $$\begin{aligned} \Pi =\begin{pmatrix} 0.0041&{}0.0002&{}-0.00078&{}0.0002\\ 0.0002&{}0.0078&{}0.005&{}0.0088\\ -0.00023&{}0.00065&{}0.0098&{}0.0045\\ 0.0023&{}0.0089&{}0.0023&{}0.0019 \end{pmatrix}. \end{aligned}$$

As a result, the following is the relevant P.D.F $\mathcal {U}(\tilde{\Psi })=\frac{1}{4\zeta _{2}^{2}}\vert \Pi \vert ^{-1/2}\exp \Big (\frac{-1}{2}\tilde{\Psi }\Pi ^{-1}\tilde{\Psi }^{\textbf{T}}\Big ).$ where

$$\begin{aligned} \tilde{\Psi }=\Big (\ln \frac{{\textbf{S}}_{\jmath }}{901670.79},\ln \frac{{{\textbf{P}}_{\textbf{m}_{1}}}_{\jmath }}{702301.67},\ln \frac{{{\textbf{P}}_{\jmath }}}{2034892.65},\ln \frac{{{{\textbf{P}}_{\textbf{T}}}}_{\jmath }}{1568923.23}\Big ). \end{aligned}$$

Consequently, the four marginal D. Fs of $\tilde{\Psi }$ are as follows:

$$\begin{aligned}{} & {} \frac{\partial \mathcal {U}}{\partial {\textbf{S}}_{\jmath }}=11.98231\exp \big (-139.6712(\ln {\textbf{S}}_{\jmath }-10.6740)\big ),~~~~\frac{\partial \mathcal {U}}{\partial {{\textbf{P}}_{\textbf{m}_{1}}}_{\jmath }}=2.0092\exp \big (-54.1299(\ln {{\textbf{P}}_{\textbf{m}_{1}}}_{\jmath }-6.5512)\big ), \nonumber \\{} & {} \frac{\partial \mathcal {U}}{\partial {{\textbf{P}}_{\jmath }}}=2.1200\exp \big (-67.0012(\ln {{\textbf{P}}_{\jmath }}-10.4501)\big ),~~~~\frac{\partial \mathcal {U}}{\partial {{\textbf{P}}_{\textbf{T}}}_{\jmath }}=7.0013\exp \big (-301.1034(\ln {{\textbf{P}}_{\textbf{T}}}_{\jmath }-7.8850)\big ). \end{aligned}$$

**Figure 13:**
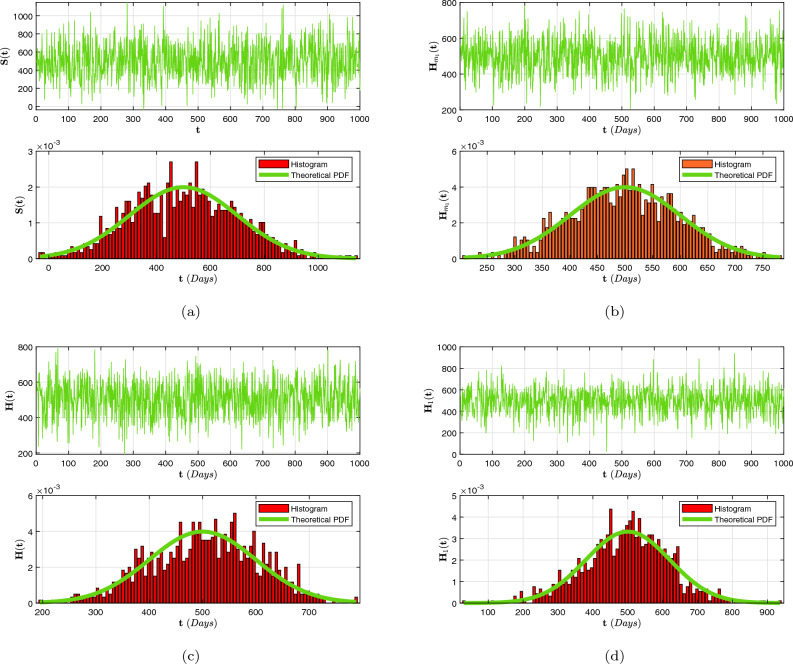
Time evaluation plots for deterministic-probabilistic co-infection H/A-P models (4), (5) and (16) with the impacts of antiretroviral therapy and post-exposure prophylaxis using CFD operator having FO $\omega =0.98,$ low intensities and ICs (1000, 250, 150, 90).

Finally, population concentrations oscillate according to the quasi-stable equilibrium $\textbf{U}^{*}$, as seen in Figure 13 (**a**-**d**).

## Conclusions

In this article, a deterministic-stochastic model is being suggested to investigate the potential transmission of the co-dynamics of H/A-P. Taking into account the deterministic fractional model and stochastic approach, we have provided the qualitative characteristics such as positivity and boundedness, reproduction number and their allied outcomes for co-infection model (4), global positive solution and unique erogdicity for the co-dynamics of (16). Besides that, applying the Khasminskii notion and a suitable Lyapunov function, the existence of a stationary distribution in model (16) was analytically verified. Additionally, an accurate representation of the P.D.F regarding a quasi-equilibrium point of the random-perturbed pneumonia model constitutes one of this research’s particularly noteworthy discoveries. In fact, it has been determined that the validity and strength of our numerical outcomes and modeled estimates have been provided in a piecewise fractional DEs context. Furthermore, the outcomes of this study shed a spotlight on the P.D.F and stationary distribution of the probabilistic multidimensional framework at its quasi-equilibrium point. Although the ABCFD, CFFD and CFD have been demonstrated to be efficient in documenting various interaction practices, we contend that their ability to accomplish this effectively may be hindered by the vastness of biological systems. It follows that oscillation might eliminate signals that are widely dispersed, despite leaving infectious diseases uncontrolled. According to the statistical correspondence, the stochastic criterion has been determined from $\mathbb {R}_{0}^{\mathbb {S}}=2.1045>1$. To demonstrate the effects of saturated incidence patterns and multiple interventions on the progression of both ailments, numerical evaluations are applied. More emphasis is placed on the impact of random white noise concentrations. Key findings from the modified model’s numerical assessment are outlined below:

1. The modified structure was fitted using a stochastic threshold calculated at $\mathbb {R}_{0}^{\mathbb {S}}=2.1045>1$ to the actual H/A-P data (shown in Fig. 2).
2.  When the pneumonia intervention percentage of individuals contaminated with H/A-P increased to $\Im _{2}=0.0021$/day and the mortality rate increased to $\eta _{1}=0.00034$/day, as shown in Fig. 12a–i, minimal instances of infestations were determined for all afflicted factors, which included individuals with co-infections H/A-P infections. Predicting how pneumonia is propagated by population mobility and random disturbances was challenging until this research was conducted. The research advances our knowledge of why pneumonia still exists around the globe. Regarding stochastic pneumonia systems, including community propagation, it is challenging to define adequate requirements for infection eradication considering the restrictive nature of computational approaches. On the other hand, we also offer a simulation of the disease’s disappearance. To determine the necessary requirements for pneumonia’s endurance and extermination, additional investigation needs to be performed. However, further studies to enhance the current work with fewer constraints may open up certain novel avenues for study. It is possible to construct more effective procedures for the proposed approach by making certain additional grounded hypotheses. Time-dependent interface intensities and time delays can additionally be taken into account by the algorithm. For both viral infections, asymptomatic classifications can also be taken into account, while data from both illnesses can be utilized to fit the models more precisely. We will examine the effects of varied diffusion rates and nonlinear Lévy noise on the dynamics of both illnesses in the future.

## Data Availability

The datasets used and/or analyzed during the current study available from the corresponding author on reasonable request.
